# From CNNs to SAM: A Survey of Deep Learning Techniques for Liver Tumor Segmentation in CT Images

**DOI:** 10.1109/access.2025.3631322

**Published:** 2025-11-10

**Authors:** NEMAN ABDOLI, YOUKABED SADRI, PATRIK GILLEY, KE ZHANG, YONG CHEN, YUCHEN QIU

**Affiliations:** 1 School of Electrical and Computer Engineering, University of Oklahoma, Norman, OK 73019, USA; 2 Stephenson School of Biomedical Engineering, University of Oklahoma, Norman, OK 73019, USA; 3 Department of Radiation Oncology, University of Oklahoma Health Sciences Center, Oklahoma City, OK 73104, USA

**Keywords:** Artificial intelligence, CT imaging, deep learning, liver tumor segmentation

## Abstract

Accurate liver tumor segmentation is a critical component of clinical assessment, forming the basis for treatment planning, therapy response monitoring, prognostic assessment, and the delivery of precision medicine. However, in real-world clinical practice, this task remains particularly challenging. The intrinsic diversity of liver tumors —manifested in variations of shape, texture, size, and location—combined with the similarity of neighboring organs, indistinct tumor boundaries, and inconsistencies in image acquisition conditions, makes accurate liver lesion segmentation particularly difficult. In clinical practice, traditional segmentation methods are often used due to their interpretability and lower computational requirements. These approaches are labor-intensive and time-consuming, especially when dealing with 3D medical images, making them impractical for large-scale or real-time applications. In recent years, deep learning (DL) models have gained considerable attention for automating liver lesion segmentation. In this comprehensive review, we analyze over 100 research papers focused on DL-based segmentation of liver tumors from computed tomography (CT) images. This survey examines these studies across multiple dimensions, including input data, model architecture, and evaluation metrics. By exploring both pioneering contributions and emerging trends, we highlight the impact of various methodological choices and address the associated limitations of current approaches.

## I. INTRODUCTION

Liver cancer is a highly lethal disease for both men and women in the United States. It is estimated that 30,090 cancer-related deaths nationwide will occur in 2025 [1]. Medical imaging is one of the critical tools used to assist oncologists in the diagnosis and treatment of liver cancer patients [2]. Various imaging modalities, including computed tomography (CT), contrast-enhanced CT, magnetic resonance imaging (MRI), positron emission tomography (PET), and ultrasound, are commonly used for liver cancer diagnosis [3]. CT remains the primary diagnostic tool due to its affordability, widespread availability, and fast image acquisition speed [4], [5], [6], [7]. In clinical practice, precise tumor segmentation is essential. It supports treatment response evaluation using RECIST, with assessments relying on accurate measurements of the longest tumor diameter to determine whether therapy should be continued, adjusted, or discontinued [8]. Accurate segmentation is also critical for surgical resection planning and for delivering radiation therapy, ensuring that doses are directed to the tumor while sparing adjacent healthy tissues [9], [10]. Prior studies have demonstrated that excessive radiation to surrounding liver tissue increases the risk of radiation-induced liver disease and recurrence in those areas [11], [12]. Moreover, numerous investigations have shown that changes in tumor size and volume are strongly associated with improved survival outcomes in liver cancer patients [13], [14], [15], [16]. However, achieving precise segmentation remains challenging due to the heterogeneity of liver tumors—characterized by irregularities in density, shape, and unclear tumor boundaries [17], [18], [19]. Despite advancements, liver tumor segmentation still faces significant challenges and requires continual improvement through advanced techniques and clinical experience [20].

Manual CT image segmentation is a labor-intensive and time-consuming task, as radiologists must visually inspect numerous slices and delineate tumor regions [21]. To address this limitation, various semi-automatic and automatic segmentation methods have been developed to reduce clinicians’ workload and improve segmentation accuracy [22], [23], [24], [25]. However, these methods still require time and substantial user expertise [26].

With the emergence of deep learning (DL), researchers quickly began to explore its potential to address challenges in medical image segmentation, with the first application involving convolutional neural networks (CNNs) in microscopy images [27]. Compared to traditional segmentation methods, DL-based approaches offer superior accuracy and faster processing times [28], [29].

**FIGURE 1.**
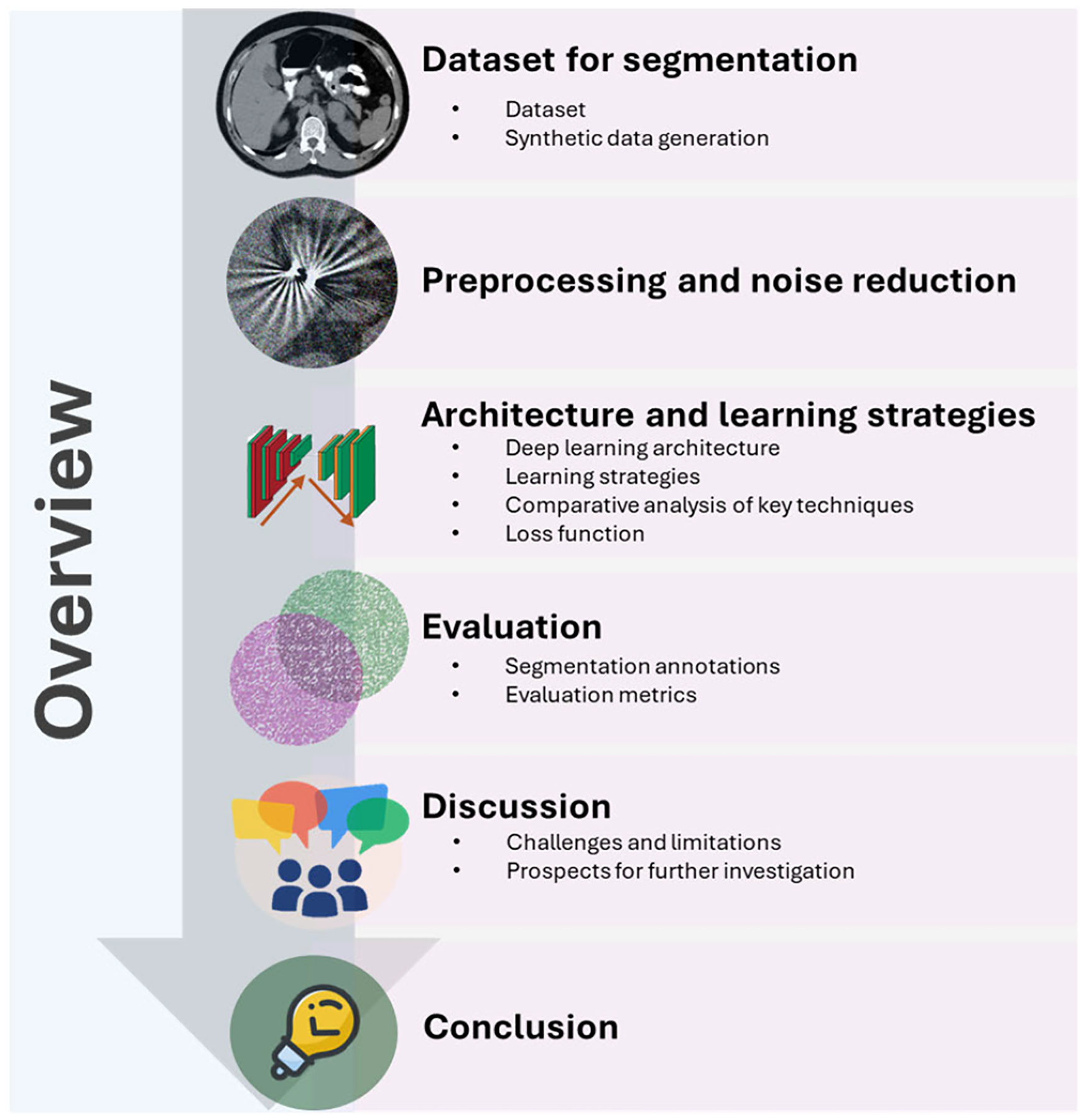
Overview of the various components of this review.

This review explores state-of-the-art DL approaches for liver tumor segmentation in CT images. It provides a comprehensive overview of advancements in the field, highlighting key contributions, existing limitations, and ongoing challenges. The review is organized as follows: Section II introduces available datasets and synthetic data generation methods. Section III discusses various DL-based network architectures, learning strategies, and loss functions designed for liver lesion segmentation. Section IV presents segmentation evaluation techniques and metrics. Section V examines current limitations in DL-based liver lesion segmentation and outlines future research directions. Figure 1 provides a visual overview of the survey’s structure, and frequently used abbreviations are summarized in Table 1.

### SEARCH STRATEGY

**FIGURE 2.**
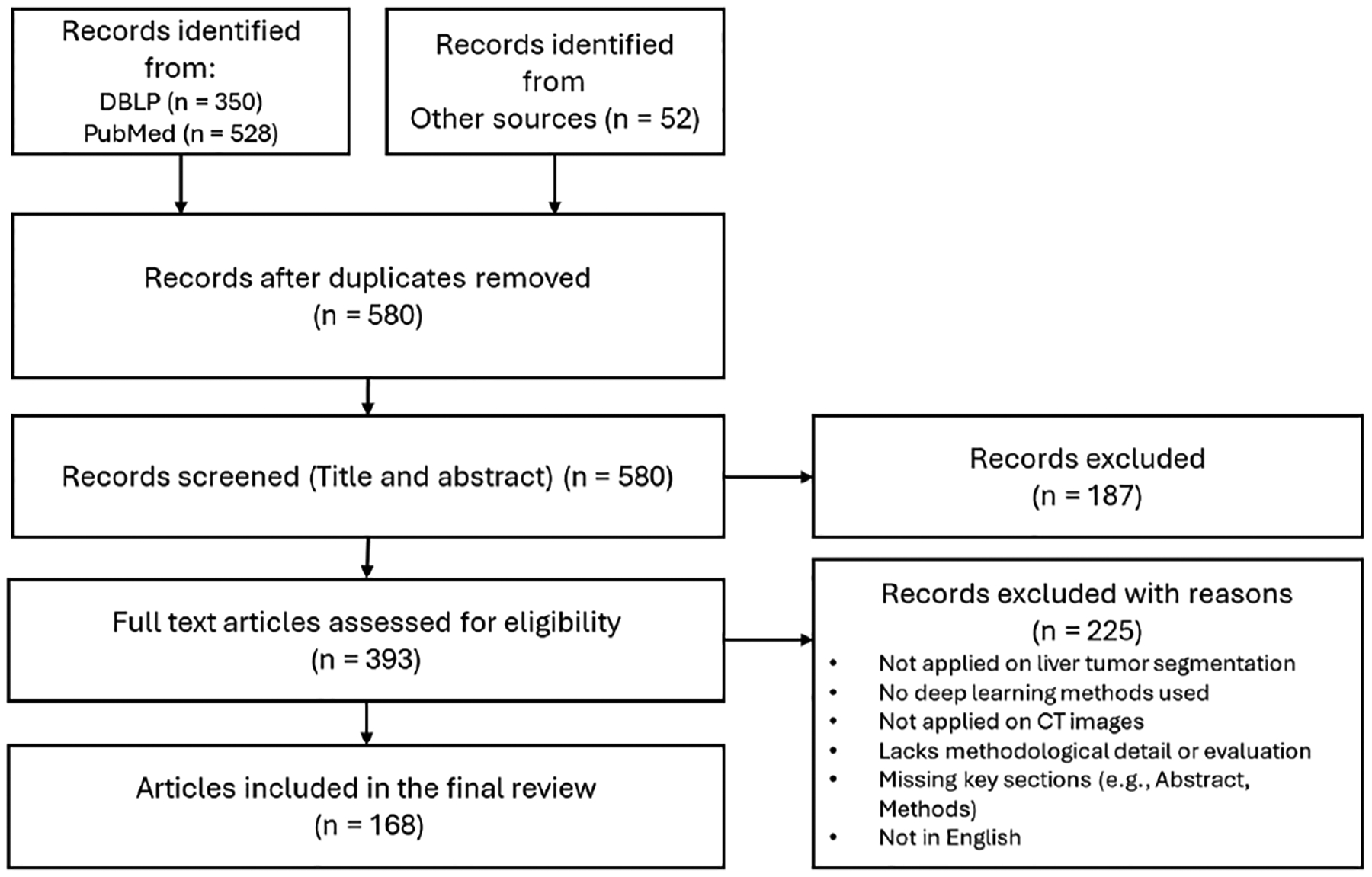
Preferred reporting items for systematic reviews and meta-analyses (PRISMA) flowchart showing study identification, screening, and selection process used in this review.

We conducted a systematic literature search in DBLP and PubMed to identify relevant studies published between 2014 and 2025. The search included peer-reviewed journal articles, conference proceedings, and non-peer-reviewed preprints. Only studies published in English were considered. DBLP and PubMed were selected as the primary databases because they support advanced search queries across both engineering and clinical domains. A summary of the search strategy is presented in Figure 2.

The search query used for DBLP was: ”(Liver | Hepatic) (Tumor | Cancer | Lesion | Pathology | Carcinoma | Neoplasm | Metastasis | Malignancy) (segment* | delineat* | extract* | localiz*)”. For PubMed, the following query was used to restrict results to relevant titles: ”((liver[Title] OR hepatic[Title]) AND (tumor*[Title] OR cancer[Title] OR lesion*[Title] OR pathology[Title] OR carcinoma[Title] OR neoplasm*[Title] OR metastas*[Title] OR malignancy[Title])) AND (segment*[Title])”. In addition to the database results, we included 52 additional studies from other sources. These included key general-purpose or foundation segmentation models. Although these were not originally developed for liver lesion segmentation, they were either applied to liver CT datasets or frequently referenced as comparative models in the literature.

After removing duplicates, 580 unique records remained. We then screened the titles and abstracts of these studies to assess their relevance. During this phase, we applied our inclusion and exclusion criteria to filter out unrelated or insufficiently detailed studies. A total of 187 records were excluded at this step for reasons such as lack of focus on liver tumor segmentation, absence of deep learning methods, or use of imaging modalities other than CT.

The remaining 393 articles were retrieved for full-text review. At this stage, we again applied the same eligibility criteria to confirm inclusion. Studies were eligible if they: (1) were published in English, (2) focused on liver tumor segmentation, (3) used deep learning models for automatic segmentation, (4) applied segmentation techniques to CT images, and (5) presented either a novel methodology, comparative analysis, or a significant improvement over prior approaches. We also required that full-text articles provide sufficient methodological detail and include essential sections such as the abstract and methods. Based on these criteria, 225 full-text articles were excluded. Ultimately, 168 studies met all eligibility requirements and were included in the final review. These comprised both liver-specific segmentation models and more general segmentation frameworks applied within the context of liver imaging.

## II. DATASET FOR SEGMENTATION

Deep learning-based medical image segmentation models require large, well-labeled datasets for effective training [30]. However, acquiring such datasets is challenging due to privacy restrictions, high annotation costs, and a lack of standardization [31], [32]. In the case of liver tumor segmentation, the limited availability of public datasets further hinders model development [33].

### A. DATASETS

**FIGURE 3.**
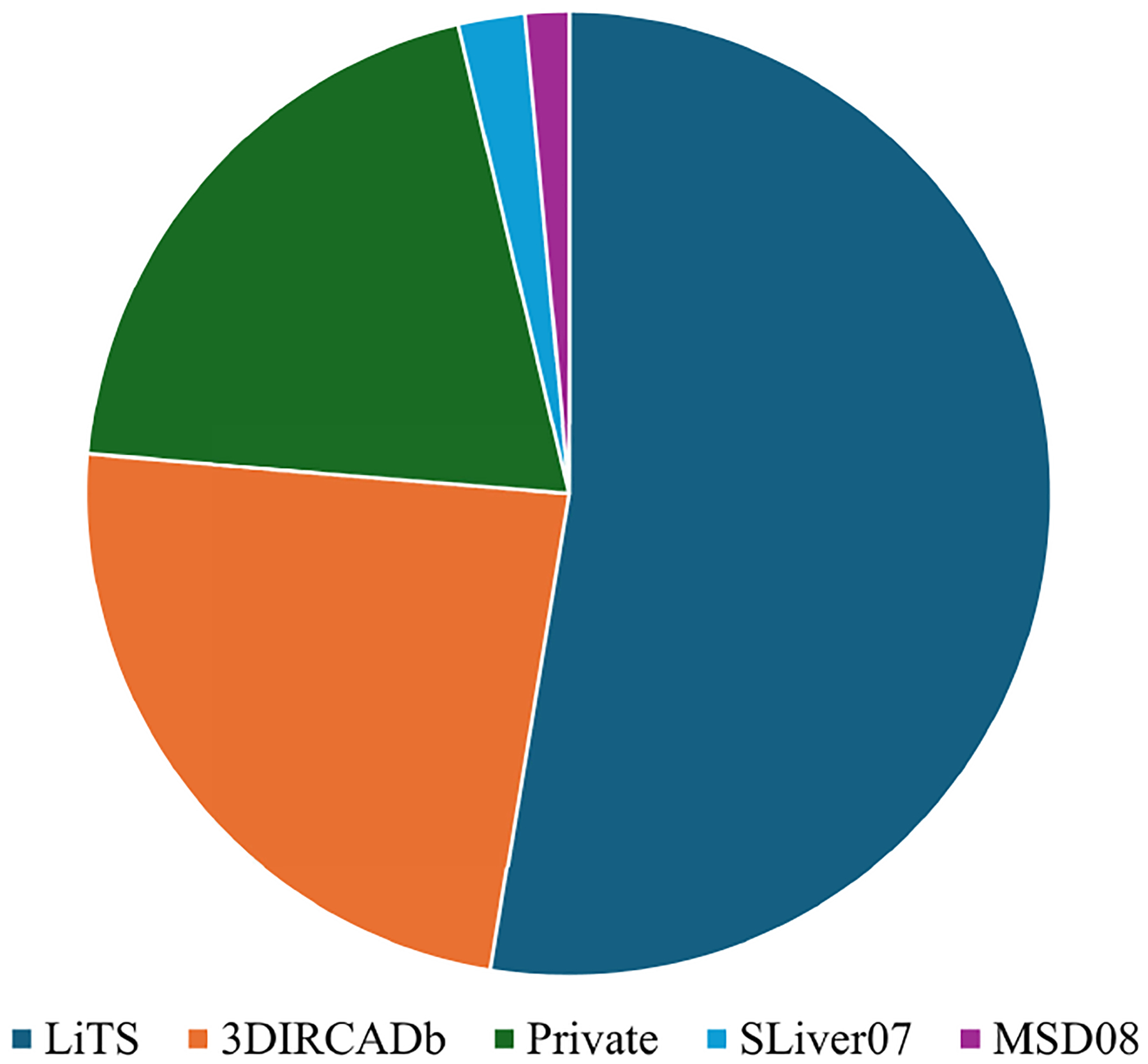
The usage frequency of different segmentation datasets in the reviewed works.

Publicly available datasets are essential for liver tumor segmentation research. To support this need, several datasets have been introduced, sourced from medical imaging competitions and academic institutions. In liver tumor segmentation studies, it is common to segment the liver first before identifying tumor regions [34]. As a result, datasets can be classified into two categories: (1) general liver segmentation datasets, which contain liver images without specific tumor annotations, and (2) liver tumor segmentation datasets, which provide detailed tumor annotations. Table 2 summarizes the key specifications of these publicly available datasets. Figure 3 illustrates the frequency of dataset usage in the literature reviewed in this paper.

General liver segmentation datasets provide CT or MRI images of the liver but do not include specific tumor annotations. The SLiver07 dataset [35], introduced during the MICCAI 2007 conference, was one of the first challenges dedicated to automated liver segmentation, featuring 30 contrast-enhanced CT scans. The CLEF 2015 dataset was developed as part of a liver CT benchmark, providing 60 CT volumes for structured reporting rather than segmentation tasks. The BTCV multi-atlas labeling challenge [36], hosted by MICCAI, was designed to extend segmentation beyond the skull vault to include abdominal regions such as the liver, kidneys, gallbladder, esophagus, and stomach, with 50 venous-phase contrast-enhanced CT scans specifically for liver segmentation. The VISCERAL dataset [37], developed in collaboration with the IEEE international symposium on biomedical imaging (ISBI), contains 120 whole-body MRI and CT scans, made available on Microsoft Azure for automated anatomy localization and segmentation. More recently, the CHAOS challenge [38] introduced 40 CT volumes and 120 MRI volumes for healthy abdominal organ segmentation, with liver annotations included.

Although these datasets contribute significantly to liver segmentation research, they do not include well-defined patient cohorts with lesions or provide comprehensive segmentation of both the liver and its tumors. To address this, more specialized datasets with detailed liver tumor annotations have been developed. The LTSC08 dataset [39], was introduced during MICCAI 2008 and contains 30 CT scans specifically focused on liver tumor segmentation. Another notable dataset, MIDAS-LT, was part of the MIDAS initiative funded by the national library of medicine (NLM) in the USA under the imaging methods assessment and reporting (IMAR) project. MIDAS-LT consists of four CT scans annotated by up to three radiologists for liver tumor segmentation, although it does not include a liver mask. The 3D-IRCADb dataset [40], compiled by the IRCAD institute in France, includes 22 anonymized venous-phase CE-CT scans, with tumor annotations available in a subset of the cases. This dataset is divided into two parts: 3D-IRCADb01, which includes 15 cases with hepatic tumors, and 3D-IRCADb02, which consists of two additional CT scans with segmentation of other abdominal organs. The cancer genome atlas liver hepatocellular carcinoma (TCGA-LIHC) dataset [41] is part of a larger initiative linking cancer phenotypes to genotypes using clinical images from the cancer genome atlas (TCGA). This dataset includes 1,688 multi-modal images from 97 subjects, with 75 CT scans specifically focused on the liver.

Challenges designed for liver tumor segmentation have further contributed to the development of benchmark datasets. The liver tumor segmentation (LiTS) challenge [33], held at both ISBI and MICCAI in 2017, provided ground-truth labels for both liver and tumor segmentation. This challenge aimed to automate tumor detection in CT volumes and estimate tumor burden, in addition to standard liver segmentation. The medical segmentation decathlon (MSD) challenge at MICCAI 2018 further emphasized model generalization across ten different biomedical segmentation tasks, including liver segmentation [42]. Two tasks within this challenge, MSDC-T3 and MSDC-T8, are particularly relevant to liver tumor segmentation. MSDC-T3 is closely related to the LiTS dataset, while MSDC-T8 consists of 443 portal venous-phase CE-CT scans with segmentation annotations for tumors and vessels. The Barts CRL dataset [43] provides contrast-enhanced staging CT scans from colorectal cancer patients across three London hospitals annotated for liver metastases segmentation and classification of benign versus malignant lesions.

These datasets collectively provide a strong foundation for training and evaluating liver tumor segmentation models, addressing the need for both general liver segmentation and tumor-specific annotation. However, limitations such as small dataset size, annotation variability, and limited generalization to unseen clinical data remain significant challenges in developing robust deep learning-based segmentation methods [32]. Low generalizability suggests that the model may be overfitted to the training data [44]. To mitigate this and improve model performance, more specialized methods have been adopted in liver tumor segmentation [45].

### B. SYNTHETIC DATA GENERATION

Synthetic data generation has emerged as a promising solution to alleviate the data scarcity and the constraints of patient privacy. This is a popular method to enhance model training and improve generalization [46]. In this review, we categorize the explored augmentation methods into three classes: simple manipulations, synthetic image generation through deep learning methods, and artificial image synthesis using heuristic approaches.

#### 1) BASIC IMAGE MANIPULATIONS

Traditional data augmentation techniques involve simple image transformations, including cropping, mirroring, translation (shifting), scaling, rotation, adding random or zero-mean Gaussian noise, random region erasing, affine or elastic deformations, intensity adjustments, sharpening, blurring, and edge enhancement [44]. Typically, a combination of these transformations is applied to reduce overfitting, such as reflection, translation, scaling, and rotation [47], [48].

The test-time augmentation approach applies transformations during inference, creating an ensemble effect that enhances the robustness of predictions [44], [49]. Techniques such as affine, pixel-level, or elastic transformations are used to better estimate uncertainty during inference [50]. While traditional augmentation methods help improve model performance by introducing variation, they remain limited in their ability to generate entirely new training samples with diverse tumor characteristics [44], [51].

#### 2) DEEP LEARNING-BASED SYNTHETIC DATA GENERATION

Deep learning-based augmentation methods offer a more generalizable framework by generating entirely new and diverse training samples [52]. Key techniques include adversarial training, generative adversarial networks (GANs), and diffusion models.

Adversarial training enhances model robustness by leveraging networks with opposing objectives. One network generates perturbed images to mislead a competing classifier which exposes model vulnerabilities and improves feature extraction [53], [54]. This approach helps models learn invariant features, making them more resilient to variations. In liver tumor segmentation, adversarial densely connected networks (ADCNs) have improved accuracy by optimizing both cross-entropy (CE) and adversarial losses. A discriminator network is used to refine segmentation by comparing deep fully convolutional network (DC-FCN) outputs with ground truth labels [55].

**FIGURE 4.**
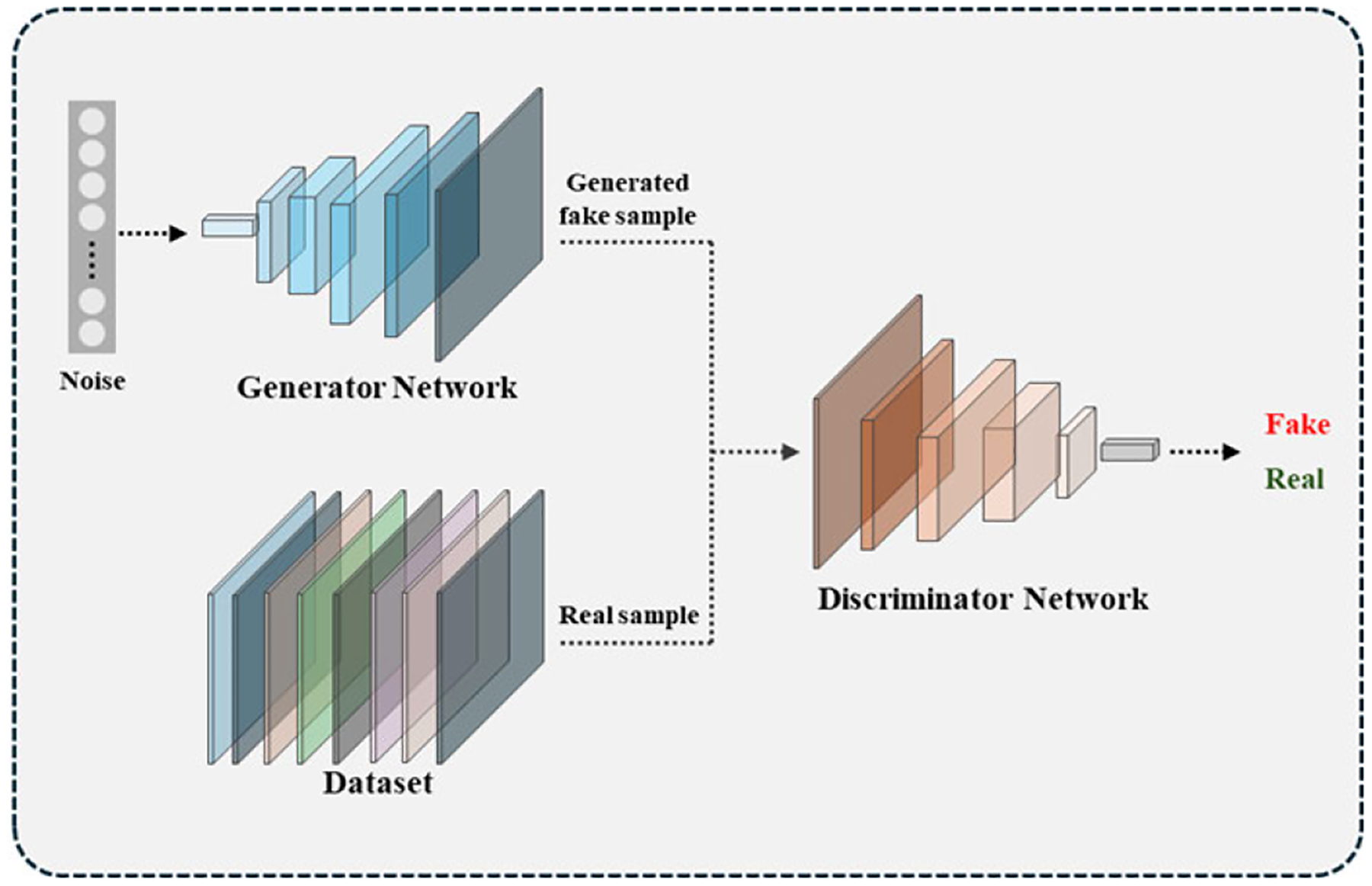
GAN architecture with a deep CNN (DCNN) generator and discriminator, used in [63].

GANs, first introduced by Goodfellow et al. [56], are widely used in medical image analysis to generate realistic synthetic data [57]. A typical GAN consists of a generator, which creates artificial samples, and a discriminator, which distinguishes the real from the fake data. The overall architecture of a typical GAN is illustrated in Figure 4. These models have been successfully applied to liver segmentation [58], [59], [60], liver tumor segmentation [61], [62], and liver lesion classification [63]. In one study, separate GAN models were used for each lesion class to synthesize 2D liver lesion Regions of Interest (ROIs) [64]. Another study introduced a GAN for free-form 3D lesion synthesis in CT images, allowing users to define the region, shape, and size of the tumor using a mask [65]. A similar study employed a GAN-based liver lesion synthesis network to generate lesion textures, where the shape, size, and rotation of the synthetic lesions were controlled using a method based on principal component analysis (PCA) [66]. Synthetic images often lack realistic lesion details, which can negatively impact AI model performance on real CT images. To address this issue, researchers trained GANs using gray-level co-occurrence matrix (GLCM) features. By focusing on liver parenchyma and tumor regions, this approach emphasized texture, shape, and grayscale attributes, resulting in more realistic synthetic liver tumor CT images [67].

Diffusion models offer a promising alternative to GANs for medical image synthesis, addressing challenges such as mode collapse and training instability [68]. These models generate high-quality, diverse synthetic images through a two-step process: forward diffusion (adding noise), and reverse diffusion (removing it to reconstruct the original input) [69]. Diffusion probabilistic models have found successful applications in various domains, including image generation tasks [69], [70]. For instance, DiffTumor [71] introduced a diffusion-based framework designed to generate realistic tumors that generalizes across different organs. Leveraging diffusion models for synthetic liver tumor generation provides a promising avenue for enhancing segmentation accuracy [72].

#### 3) HEURISTIC-BASED SYNTHETIC DATA GENERATION

Deep learning-based generative models still require pixel-wise tumor masks to learn pathological patterns from annotated tumor areas [57]. However, obtaining accurate annotations is challenging, and these models are prone to both inter-annotator and intra-annotator variability, even when provided by medical experts [73]. To reduce the cost and effort of manual annotation, researchers have explored heuristic methods for generating synthetic tumors as an alternative. Realistic tumor synthesis requires careful consideration of factors such as shape, intensity, size, location, and texture. For instance, in a study [74], liver and brain tumors were synthesized for pre-training, enabling the model to adapt to tumor segmentation within the same organ under low-annotation conditions. By integrating artificial anomalies into normal organ images, shape and texture were effectively captured. Another investigation [75] examined the impact of using handcrafted tumors for training deep learning models for liver tumor segmentation. The findings indicated that incorporating synthetic tumor data into training improved segmentation performance, demonstrating its potential as a viable augmentation technique. However, early heuristic methods did not account for spatial awareness, occasionally resulting in synthetic tumors overlapping with vascular structures. To address this, Hu et al. [76] proposed a strategy for synthesizing realistic liver tumors of varying sizes based on clinical knowledge. Evaluating the generalizability of these methods across tumors in different organs remains an intriguing direction for future research. Overall, the discussed methods vary in complexity and realism. Table 3 consolidates these approaches, highlighting their relative strengths and weaknesses.

## III. PREPROCESSING AND NOISE REDUCTION

Preprocessing of CT images is a crucial step before training deep learning models, as it enhances quality and ensures consistency across datasets. CT scans inherently suffer from low contrast and multiple noise types introduced during image acquisition and reconstruction. These include additive Gaussian noise (random pixel fluctuations), quantum noise (graininess from limited X-ray photons), impulse or salt-and-pepper noise (isolated extreme values), speckle noise, and structured artifacts such as streaks, ring or blockiness [77], [78]. Such distortions obscure fine anatomical details and textures, complicating segmentation tasks for deep learning models that depend on clear boundaries.

To address these challenges, preprocessing pipelines commonly integrate denoising alongside standard steps such as HU adjustments, intensity normalization, and resampling. Traditional denoising techniques include spatial-domain filters (median, bilateral, non-local means) and transform-domain approaches [79]. For instance, in liver segmentation studies, 7×7×7 median and bilateral filters have been applied to suppress noise [80], while more advanced approaches like anisotropic diffusion and adaptive likelihood estimation have shown promise in improving contrast between tumors and surrounding tissue [81].

In recent years, DL-based preprocessing has become increasingly effective at suppressing CT noise while preserving anatomical structures [82]. CNNs [83], GANs [84], deep residual networks [85], and transformers [86] have been applied to low-dose CT denoising. For example, DnCNN-SR improved liver margin segmentation [87], and RED-CNN or MAP-NN significantly improved liver vessel segmentation accuracy with nnU-Net [88]. However, while these approaches reduce noise more effectively than traditional filters, they may also over-smooth fine structures or introduce artificial features, underscoring the need for careful validation in clinical applications.

## IV. ARCHITECTURE AND LEARNING STRATEGIES

### A. DEEP LEARNING ARCHITECTURE

#### 1) CNN AND FCN

CNNs were introduced to overcome the limitations of traditional machine learning (ML) methods by enabling automatic feature extraction through convolutional operations. They learn spatial hierarchies of features, improving performance in medical image segmentation, including liver tumor segmentation [89]. Several studies have demonstrated the effectiveness of CNNs in liver tumor segmentation. For instance, Li et al. [17] employed a patch-based CNN approach to segment liver tumors, assessing the impact of different patch sizes on 2D CT images. They found that a 17×17 patch size effectively focused on tumor regions, outperforming traditional ML methods such as AdaBoost, Random Forest, and support vector machines (SVMs). Another study [90] proposed a three-step approach to segment the liver coarsely and finely before isolating tumors using CNNs. Similarly, [91] implemented a 3D patch-based CNN for fine liver segmentation following an initial coarse segmentation. In another study [92], researchers combined 2D CNNs with patient-specific 3D CNNs to further enhance segmentation of follow-up CT scans. Additionally, a two-path CNN using original, normalized, and texture-encoded images was developed to optimize patch sizes for more effective segmentation [93]. Despite their effectiveness, CNNs struggle to capture long-range dependencies, maintain structural integrity, and efficiently handle pixel- or voxel-level loss computation, especially for large anatomical structures [94].

Fully convolutional networks (FCNs) extend CNN-based segmentation by replacing fully connected layers with convolutional layers. This reduces the number of parameters and enables the model to process entire images at once, improving contextual understanding [24]. The first application of FCNs for semantic segmentation showed promising results [95], leading to their adoption in liver tumor segmentation. Several studies have since employed FCNs in liver tumor segmentation. For example, one study [96] combined a 2D FCN with 3D deformable model optimization [97], based on local cumulative spectral histograms and non-negative matrix factorization (NMF), to improve segmentation accuracy. Another study used multi-phase CT data (arterial, portal venous, delayed) within a multi-channel FCN (MC-FCN) to fuse phase-specific high-level features [98]. One approach implemented 3D convolutions in an FCN architecture for coarse-to-fine liver segmentation, effectively masking non-liver tissues and initializing a level-set method to refine tumor boundaries [99]. This method also enhanced edge detection by incorporating a fuzzy c-means (FCM) probabilistic mask. To address the complexity of 3D convolutions, a lightweight hybrid 2D/3D model employed separable factorization to reduce the number of parameters while maintaining high performance [100]. Cascaded frameworks have also been explored. A two-stage FCN was used to first identify the liver as a ROI, then segment lesions within that region [101]. Sequential FCNs combined with conditional random fields (CRFs) have also been proposed to improve segmentation quality [102], [103]. Comparative studies have confirmed the superiority of FCNs over patch-based CNNs in liver segmentation and metastasis detection, highlighting their ability to leverage full-image context [104]. However, traditional FCNs suffer from a loss of spatial detail due to pooling operations, resulting in imprecise or blurred segmentation maps [105]. Introducing connections between early and late convolutional layers can help recover fine spatial details and improve overall segmentation precision [98].

#### 2) U-NET AND ITS VARIANTS

**FIGURE 5.**
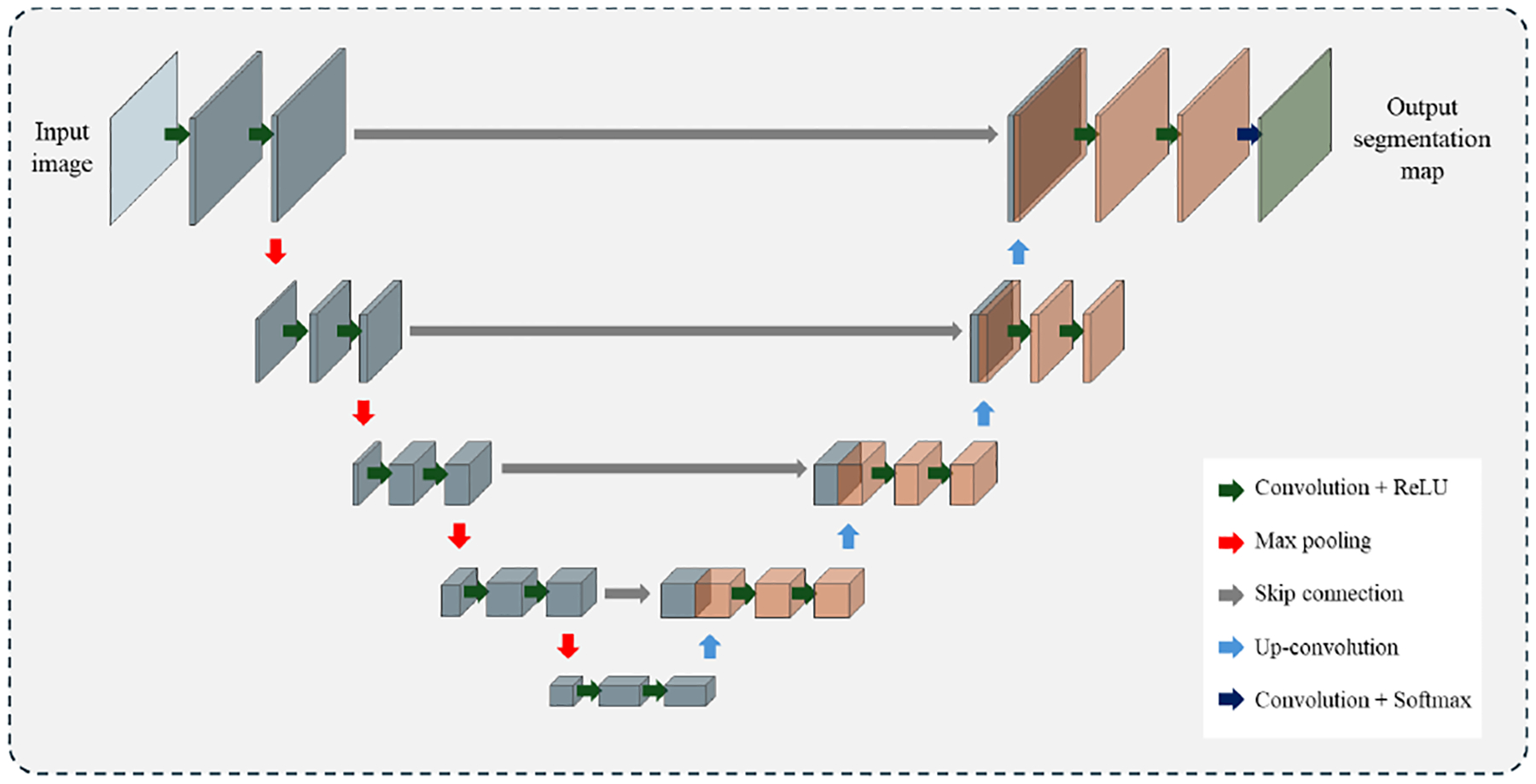
The U-Net architecture.

The U-Net architecture, initially developed for 2D electron microscopy image segmentation, has gained significant popularity in medical image research due to its effectiveness in semantic segmentation tasks [106]. It features a U-shaped symmetric encoder-decoder structure composed of convolutional and up-convolutional layers, as illustrated in Figure 5. Skip connections between corresponding encoder and decoder blocks help preserve low-level information that may be lost during pooling operations [106]. Numerous studies have investigated both the original U-Net and its modified architectures to improve liver tumor segmentation performance [48], [107], [108], [109], [110], [111], [112], [113], [114], [115]. For example, SegNet [116], a U-Net variant, replaces full feature maps in skip connections with max-pooling indices to improve efficiency while retaining essential spatial information. A modified version of SegNet was fine-tuned for liver segmentation, using a K-Means algorithm to isolate tumors within the segmented liver [51]. Various adaptations of SegNet-based models have also improved segmentation accuracy in liver tumor detection [117], [118]. CompNet, a notable U-Net variant, employs dual pathways to extract features from both the target tissue and the complementary background [119]. Based on this architecture, [120] proposed a framework for liver and lesion segmentation in CT images: liver regions were first segmented slice by slice using a 2D CompNet, the slices were then stacked into a 3D volume, and lesion detection was performed using a combination of a 2D CompNet for large lesions and a 3D CompNet for smaller ones.

To address the continuity of 3D medical images, V-Net extended U-Net into 3D. However, its relatively shallow architecture—with only a single max-pooling layer following initial convolutions—limited its capacity for multi-scale analysis [121]. This shortcoming was addressed by 3D U-Net, introduced by Çiçek et al., which has since become a widely adopted architecture for volumetric medical image segmentation [122]. Further improvements were proposed in [123], where a single 3D U-Net was trained using a three-stage curriculum learning strategy: starting with full 3D volumes, progressing to 3D tumor patches, and finally combining tumor-specific features with global context.

Given the high computational demands of 3D models, several studies have explored complexity reduction techniques, such as processing only tumor-containing slices (2.5D) or employing hybrid 2D/3D architectures. For example, UV-Net combines a 3D V-Net encoder with a 2D U-Net decoder to balance accuracy and efficiency [124], [125], [126]. Additionally, lightweight U-Net variants have emerged, utilizing depthwise separable convolutions to reduce computational overhead while preserving performance [127], [128]. The nnU-Net framework, which dynamically configures CT slice arrangements based on varying spatial adjacency, has also demonstrated enhanced segmentation precision [125].

#### 3) RESIDUAL NETWORKS (RESNET) AND THEIR EXTENSIONS

Residual networks, introduced in ResNet, enhance feature propagation and mitigate vanishing gradient issues, making them particularly valuable for deep medical image segmentation tasks [129]. Numerous studies have incorporated ResNet blocks into U-Net architectures to improve segmentation accuracy [123], [127], [130], [131], [132]. Some approaches embedded residual blocks in both the encoder and decoder stages alongside conventional convolutional layers [133]. One notable method employed pretrained ResNet50 blocks (trained on ImageNet) within the encoder, combined with feature-fusion skip connections to merge multi-scale features, thereby enhancing segmentation performance [134].

Building upon this, a cascaded U-ResNet model was proposed in [135] to simultaneously segment the liver and lesions. This architecture utilized intra-network skip connections, allowing the lesion segmentation network to refine its outputs using features extracted from liver segmentation.

Beyond standard ResNet blocks, residual connections have been extended to fractal-like structures to capture and integrate multi-scale contextual information for deep semantic segmentation. Fractal residual networks, extensively studied in liver tumor segmentation [136], [137], [138], have demonstrated robust performance. Specifically, studies in [137] and [138] reported that these networks significantly improved tumor candidate classification following initial segmentation, enabling effective handling of complex tumor morphologies.

#### 4) DENSE NETWORKS (DENSENET) AND THEIR EXTENSIONS

**FIGURE 6.**
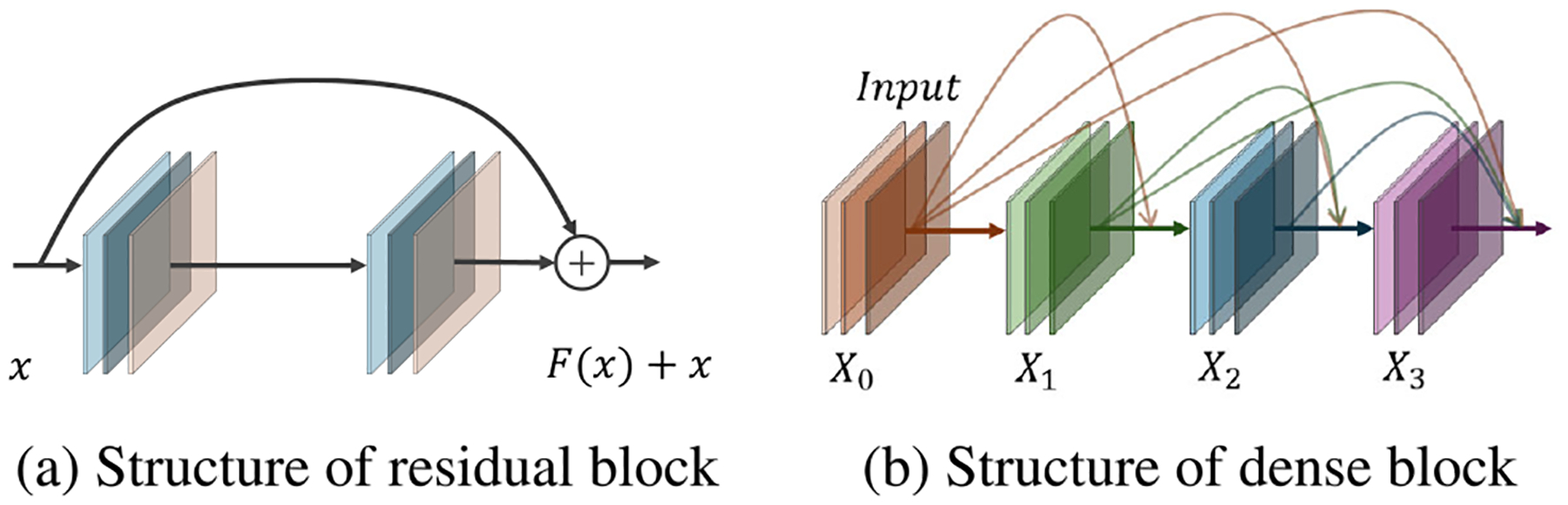
Architectural comparison: (a) A standard residual block highlighting skip connections; (b) A dense block with three layers demonstrating dense connectivity between layers.

Dense blocks, originally introduced in DenseNet [139], enhance feature propagation, gradient flow, and parameter efficiency by establishing direct connections between all layers within a network stage. Each layer in a dense block receives input from all preceding layers and passes its own feature maps forward, reducing redundancy and improving segmentation performance. Figure 6a and Figure 6b illustrate the differences between a residual block and a dense block, highlighting how dense connections promote efficient feature reuse.

To leverage these advantages in medical imaging, UNet++ [140] introduced dense skip connections to reduce the semantic gap between encoder and decoder features, thereby improving segmentation accuracy. Further utilizing dense connectivity, Kaluva et al. [123] employed dense blocks in both the encoder and decoder of a U-Net trained on CT axial slices at three different HU levels, demonstrating enhanced liver tumor segmentation performance.

Similarly, the bottleneck supervised U-Net (BS U-Net) [141] incorporated dense connections in the bottleneck layer, refining feature representations and boosting segmentation accuracy. To further optimize dense connections, H-DenseUNet proposed a two-stage hybrid approach that combined 2D DenseUNet for intra-slice feature extraction with 3D DenseUNet for volumetric feature aggregation. This design improved segmentation consistency across slices, addressing challenges associated with volumetric medical imaging [142].

While deeper networks generally improve segmentation accuracy, they also increase computational costs [128]. To address this, one study optimized a U-Net-like structure by replacing pooling layers with strided convolutions and employing depthwise separable convolutions within dense blocks. This significantly reduced the number of learnable parameters while preserving segmentation performance [143].

#### 5) MULTI-SCALE FEATURE FUSION TECHNIQUES

Deep learning-based liver tumor segmentation often employs multi-scale feature fusion schemes to improve segmentation accuracy by combining information from different spatial levels. These methods enhance performance by capturing contextual information from objects of varying sizes, reusing spatial details across network layers, and enabling deeper layers to learn more complex representations [144]. In encoder-decoder architectures, multi-scale features are typically extracted after pooling operations and fused across layers to boost liver tumor segmentation accuracy [20], [55], [134], [145], [146], [147].

Several strategies have been proposed to optimize multi-scale feature fusion. For instance, [147] introduced a multitask network that leveraged multi-scale features from both encoder and decoder stages to enhance liver lesion mask prediction and classification. Another approach utilized dense connections to propagate features across multiple scales, improving the segmentation of tumors with complex spatial structures. A study by [55] developed a segmentation framework that integrated dense feature fusion within an adversarial training setting and incorporated dilated convolutions to expand the intra-slice receptive field, thus enhancing robustness.

To further improve multi-scale learning, some models apply parallel convolutions with varying kernel sizes (e.g., 3×3, 5×5, 7×7) to effectively capture features of tumors at different scales [19], [148], [149], [150], [151]. High-resolution networks (HRNet) [149] maintain multi-resolution feature representations throughout the network, ensuring consistent segmentation performance across varying tumor sizes. Another approach, RIS-UNet [152], employs Residual–Inception–SE (RIS) blocks that integrate residual connections, parallel convolutions at multiple scales to enhance multi-scale feature fusion and improve liver tumor segmentation performance. Additionally, dilated (atrous) convolutions are widely used to increase the receptive field without significantly increasing computational cost, enabling the capture of broader spatial dependencies [153].

Atrous spatial pyramid pooling (ASPP), introduced in DeepLab, is a widely used technique that enhances segmentation by combining multiple dilation rates with global average pooling [154]. Several studies have incorporated ASPP into liver tumor segmentation frameworks [155], [156], [157], [158], [159]. For example, the TDS-U-Net network [160] integrated ASPP in the bridge section to capture multi-scale contextual features, and further extended its use to the decoder’s transition and output layers to enrich multi-scale representation. Similarly, Ma et al. [158] proposed a modified U-Net incorporating a cascaded adaptive feature extraction unit with multi-head attention blocks and ASPP modules to refine segmentation precision. Another study [159] fused ASPP features with boundary information at each layer, enabling the model to better focus on tumor structures and fine anatomical details. In addition, a comparative evaluation of U-Net, U Net++, and DeepLabV3+ on liver tumor CT datasets showed that U-Net remains robust, while newer architectures such as DeepLabV3+ can further boost performance [161].

#### 6) ATTENTION MECHANISMS

To tailor deep learning models specifically to the liver region in CT images and to limit the influence of irrelevant information, several approaches have been employed [162]. For example, one strategy uses both the CT image and a liver mask generated by a separate liver segmentation model to restrict the analysis to the liver region [102], [120]. Another approach uses liver and tumor bounding box masks as inputs to a multi-encoder-decoder architecture, thereby focusing on task-relevant features by isolating the regions of interest [163]. Attention mechanisms [164] further enhance this process by adaptively assigning weights to different regions in an image, enabling the network to focus on areas critical to the task while disregarding irrelevant ones. These mechanisms have demonstrated significant effectiveness in medical image segmentation and are widely adopted in recent research [134], [162].

Spatial attention (SA) methods refine feature selection by assigning importance scores to different regions based on mean and max pooling operations [165]. This technique enhances segmentation accuracy by prioritizing tumor-specific regions while reducing background influence [166], [167], [168]. For instance, one study used a spatial attention mechanism to merge low-level spatial information and high-level semantic information, improving fusion efficiency in liver tumor segmentation [166]. Attention gates (AGs) build on this by combining input features with a coarse-scale gate signal to generate spatial attention weight maps. Several studies have utilized AGs to improve segmentation performance [159], [160], [169], [170], [171]. Attention U-Net [172], for instance, incorporates AGs into skip connections, suppressing irrelevant features in liver and tumor segmentation tasks. Another method includes residual attention mechanisms (RA-UNet), which use a trunk branch to retain original feature information and a soft mask branch to selectively enhance or suppress specific features [168].

Unlike spatial attention, channel attention (CA) mechanisms focus on reweighting feature channels to emphasize relevant attributes of liver tumors [165]. One study incorporated CA within a novel skip connection strategy in U-Net architectures for CT liver image segmentation [173]. This approach intentionally excluded spatial attention to preserve spatial continuity, mitigate overfitting, and maintain model simplicity. Squeeze-and-excitation (SE) modules [174] exemplify this approach by applying global average pooling (GAP) to recalibrate channel weights, improving sensitivity to liver tumor-relevant features [136], [175], [176]. Studies incorporating SE modules in Res-UNet architectures [110], [134] and densely connected networks [136] have demonstrated improved segmentation accuracy by refining both low-level and high-level feature representations. MS-UNet [177] incorporates the SE module to enhance channel-wise feature recalibration and improve the network’s ability to describe high-level features. Additionally, HfRU-Net [178] modified skip pathways using SE modules and employed an ASPP module in the bottleneck to enhance segmentation performance while reducing computational complexity.

Some architectures combine spatial and channel attention to further enhance feature refinement in liver tumor segmentation [148], [179], [180]. For instance, a study [145] proposed an approach that refines spatial and channel features by highlighting the difference between the encoder and decoder through a subtractive operation. The convolutional block attention module (CBAM) [181] combines channel and spatial attention to achieve comprehensive feature refinement, inheriting the strengths of both mechanisms for medical image classification and segmentation. Researchers also introduced a context-guidance (CG) branch and a boundary-learning (BL) branch to enhance contextual feature extraction and boundary detection for tumors [155]. To improve boundary representation, CBAMs were integrated into their model. A multi-attention network (MANet) was proposed to enhance liver tumor segmentation by integrating four attention mechanisms: skip connection AGs, channel attention, spatial attention, and CBAM [34]. The skip connection attention gates capture crucial shallow features from the encoder, which are concatenated with semantically rich features from the decoder. Additionally, they incorporated both GAP and global max pooling for channel attention, demonstrating improved inter-channel relationships compared to conventional methods. Building on these hybrid attention strategies, the multi-scale feature fusion attention network (MFA-Net) leveraged a U-Net architecture and incorporated modified versions of SE and GAP modules to implement channel and spatial attention, addressing the lack of spatial awareness in FCNs [176].

While spatial and channel attention mechanisms enhance feature refinement in static images, medical imaging often involves sequential data, such as dynamic scans or time-series inputs. In such cases, temporal attention is used for temporal relation modeling [162], making it particularly useful for enhancing inter-slice consistency [182], processing multi-frame sequences or videos, such as those used in clinical settings [183], [184].

#### 7) SELF-ATTENTION AND TRANSFORMERS

**FIGURE 7.**
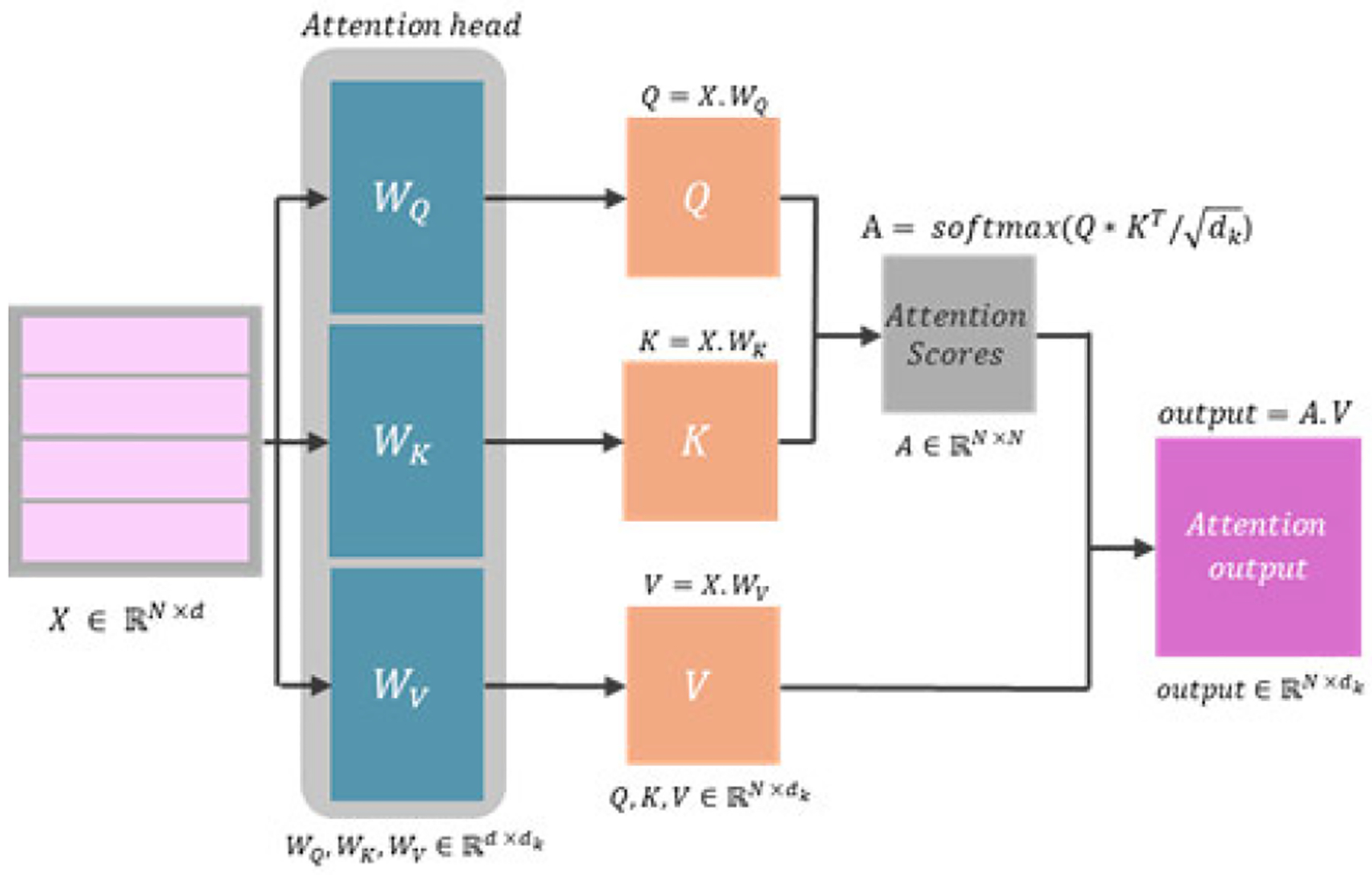
Self-attention mechanism.

Self-attention mechanisms capture long-range dependencies by computing pairwise relationships across all elements of the input, integrating both spatial and channel information [162], [164]. This mechanism is illustrated in Figure 7. Unlike conventional attention mechanisms, which primarily focus on local relationships—whether within a single slice (intra-slice) or between adjacent slices (inter-slice)—self-attention considers pairwise relationships across all input elements. Axial attention [185] and criss-cross attention [186] were introduced to improve computational efficiency by focusing on axial or cross-pattern relationships, thereby enhancing segmentation performance. Criss-cross attention has been successfully applied in the bottleneck of a U-Net-like architecture with dense blocks, improving feature propagation and segmentation precision [187]. A similar approach employed axial attention in a comparable context [185], demonstrating its ability to capture long-range dependencies with reduced computational complexity. Additionally, a study [149] utilized criss-cross attention to model long-range relationships between feature pairs in the same row and column of multi-scale encoder feature maps, significantly reducing the computational cost of traditional self-attention.

Building on these techniques, multi-head self-attention (MHSA) has been widely used in medical image segmentation. MHSA allows the model to capture a broader variety of relationships and patterns by employing multiple attention heads operating on different subspaces of the feature representations [188]. One study integrated MHSA and a series of dense connections in the bottleneck of a Res-UNet architecture to enhance the extraction and reuse of relevant features [158]. Another study applied MHSA in the spatial encoder of an XFuse network, which combines spatial and frequency features to improve liver tumor segmentation [189]. Inspired by MHSA, researchers developed a double-branched encoder with multi-axis aggregation Hadamard attention (MAHA) and group multi-head cross-attention aggregation to fuse information from both the Fourier domain and spatial features [190].

Attention mechanisms have also been applied to enhance multi-scale feature fusion by dynamically selecting the most relevant features across different scales, minimizing computational overhead, and improving segmentation accuracy across varying spatial resolutions [19], [134], [149], [157]. For instance, DPC-Net [19] integrates a pyramid structure with spatial and channel attention modules in the encoder, enabling the extraction of multi-level features and improving segmentation of tumors with diverse sizes, shapes, and locations. This network leverages attention mechanisms to exploit semantic and spatial correlations among pyramid features for more effective liver tumor segmentation. Similarly, [191] proposed T-MPEDNet, a Transformer-aware multiscale progressive encoder–decoder network that integrates feature recalibration and contextual attention to capture long-range dependencies. By refining multi-scale features and boundaries, it improves segmentation of small and indistinct liver tumors while achieving state-of-the-art performance on public datasets.

The breakthrough multi-head self-attention mechanism—originally introduced in transformer architectures for natural language processing (NLP) [164]—was quickly adopted in computer vision tasks, leading to the development of the Vision Transformer (ViT) [192]. In liver tumor segmentation, transformers have gained attention for their capability to model global relationships between image patches across various positions in encoder-decoder frameworks. Wang et al. proposed ACF-TransUNet [173], which integrates a transformer block into the bottleneck of a U-Net architecture and includes channel attention modules in skip connections to improve segmentation accuracy. Similarly, the attention connect network (AC-Net) [185] combines a transformer block in the bottleneck with axial attention in skip connections to enhance efficiency. Another hybrid model combined transformers and CNNs, placing a transformer module in the fourth skip connection to capture global context [193]. Kang et al. [159] proposed a dual-encoder pathway network that uses an attentional feature fusion block to integrate global semantic features from a transformer-based encoder with local features from a CNN-based encoder. More recent approaches have further advanced this trend.

Other innovative approaches have also emerged. One model used a multiple feature extractor and a fusion module to integrate semantic features from transformers, spatial features from CNNs, and edge-enhanced features [175]. Another employed a dynamic hierarchical transformer network based on the 3D U-Net architecture, introducing an edge aggregation block (EAB) to better capture fine-grained edge details [194]. In a separate study [195], a 3D transformer structure was adopted for multi-phase tumor segmentation. This model used a generative adversarial strategy to provide domain-adaptive features and minimize the feature gap across CT phases.

While transformers apply global self-attention by computing relationships among image patches, this results in quadratic computational complexity. To address this issue, Vaswani et al. [196] introduced shifted window-based local self-attention, which reduces complexity to linear levels while enabling cross-window connections through shifting. Building on this idea, a novel network—SWTR-UNet—was developed, integrating SWIN transformer blocks with ResNet blocks [197]. Similarly, [198] utilized Swin-neighborhood fusion transformer blocks (SFTB), which combine Swin transformer and neighborhood attention transformer modules. The Swin blocks capture global contextual information via shifted windows, and the added neighborhood attention (NA) operates at a per-pixel level within local neighborhoods (e.g., 3×3), enabling finer local context modeling. This fusion allows SFTB to jointly capture both global and local contextual information more effectively than Swin blocks alone. While transformers rely on computationally expensive self-attention, recent studies have proposed token-based multilayer perceptron (MLP) blocks as lightweight alternatives. For example, a modified tokenized MLP U-shaped network incorporated spatial-shift MLP blocks to enhance inter-token information flow, attention gates in skip connections to suppress irrelevant features, and a multiscale attention module for improved boundary delineation. This model achieved competitive performance on the LiTS dataset using limited number of parameters, highlighting its efficiency for real-time clinical tasks while maintaining high segmentation accuracy [199].

#### 8) VISION-LANGUAGE MODELS (VLMS) AND FOUNDATION MODELS

In recent years, foundation models have transformed AI technologies and reshaped our understanding across various domains [200]. Among these, large language models (LLMs) [201], characterized by their scale and complexity [200], have facilitated the development of models pretrained on both visual and linguistic data, leading to the emergence of vision-language models (VLMs) [202]. VLMs, such as contrastive language–image pre-training (CLIP) [203] and large-scale image and noisy-text embedding (ALIGN) [204], have significantly integrated textual supervision into vision models, garnering notable attention in medical imaging applications.

One of the most prominent foundation models in image segmentation is the segment anything model (SAM) [205], originally designed for natural image segmentation. SAM operates as a prompt-based segmentation framework, supporting both sparse prompts (e.g., points, boxes, text) and dense prompts (e.g., masks), and has demonstrated strong performance in interactive segmentation tasks [206]. In medical image segmentation, SAM’s zero-shot performance has been explored in several studies. However, its effectiveness is often limited when segmenting complex medical structures [207], [208], [209]. These limitations largely arise from the dominance of natural images in SAM’s training dataset and its lack of domain-specific medical knowledge, leading to suboptimal performance in clinical applications [207].

For instance, a study on liver tumor segmentation found that SAM’s accuracy was unsatisfactory with a limited number of prompts. However, its performance improved with more expert intervention and additional prompts [210], [211]. To address such limitations, several strategies have been developed to improve SAM’s effectiveness in the medical domain [212]. One common approach is fine-tuning SAM on task-specific medical images [213], [214]. For example, MedSAM [21] retrained SAM’s image encoder using 1.57 million image-mask pairs from 11 modalities and over 30 cancer types, while keeping the prompt encoder frozen and using only bounding box prompts. Nevertheless, full-scale fine-tuning for medical applications remains computationally expensive and data-intensive [206], [209].

To mitigate this, some studies have proposed retaining the frozen SAM encoder while replacing its decoder with task-specific alternatives optimized for medical image segmentation [215], [216]. While this preserves SAM’s pretrained visual knowledge, the results remain highly prompt-dependent, particularly when handling multi-modal medical images where domain shifts from natural images affect performance [217], [218]. Another efficient method integrates adapter modules within the SAM encoder, allowing for parameter-efficient fine-tuning and improved adaptability to medical tasks [206], [219], [220], [221]. For instance, SAMed [221] enhances medical image segmentation by incorporating low-rank adaptation (LoRA) modules into the pretrained SAM image encoder.

**FIGURE 8.**
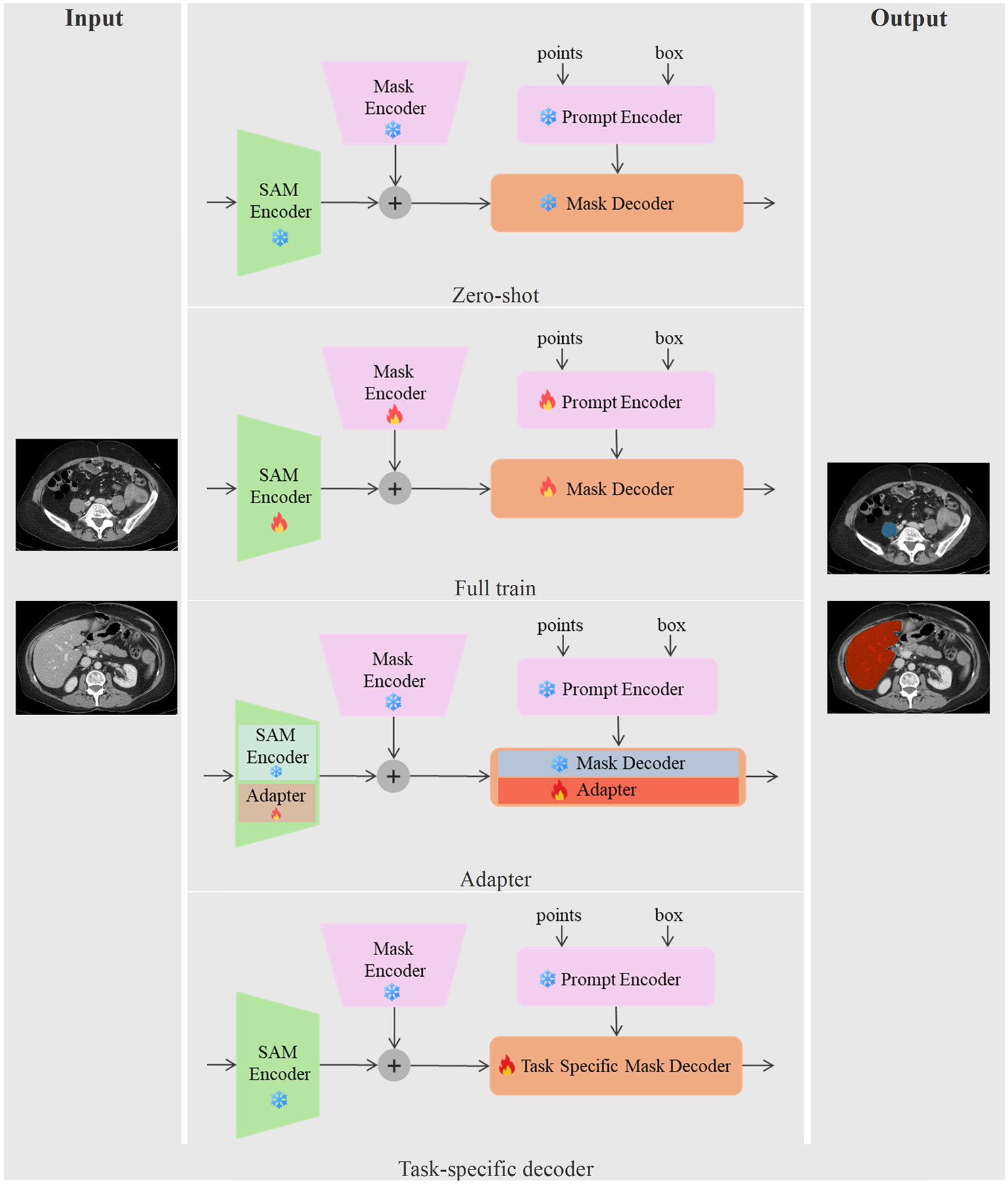
SAM adaptation methods used in medical imaging.

Despite these advancements, limited research has focused specifically on applying VLMs to liver tumor segmentation. A study by Chen et al. [222] proposed a three-stage semi-supervised framework that generates pseudo-labels, which are subsequently used as prompts to fine-tune the Med-SA model for liver tumor segmentation. Various strategies for adapting SAM to medical image segmentation are illustrated in Figure 8.

One major limitation of SAM in 3D medical image segmentation is its slice-by-slice processing of axial CT images, which ignores inter-slice spatial continuity. This limitation prevents the model from fully capturing 3D spatial relationships needed for accurate segmentation. To overcome this, researchers have explored SAM2, which applies video tracking techniques for zero-shot 3D segmentation [223], [224]. However, SAM2 has shown poor performance on CT images due to the lack of domain-specific training [225].

An alternative direction involves integrating 3D adapters into SAM, maintaining most of its pretrained parameters frozen while enriching deep features with spatial and depth information. These parameter-efficient finetuning approaches have shown promise in improving segmentation accuracy in medical imaging [226], [227], [228], [229], [230], [231]. A notable development in this field is Med-SA, which extends SAM’s capabilities to 3D medical image segmentation using trainable adapter layers [232].

Beyond architectural modifications, leveraging SAM’s predictions as prior knowledge has become a prominent trend in medical image segmentation [222], [233], [234]. For instance, [233] demonstrated that integrating SAM-generated pseudo-labels into U-Net-based segmentation models achieved performance comparable to fully supervised models.

**FIGURE 9.**
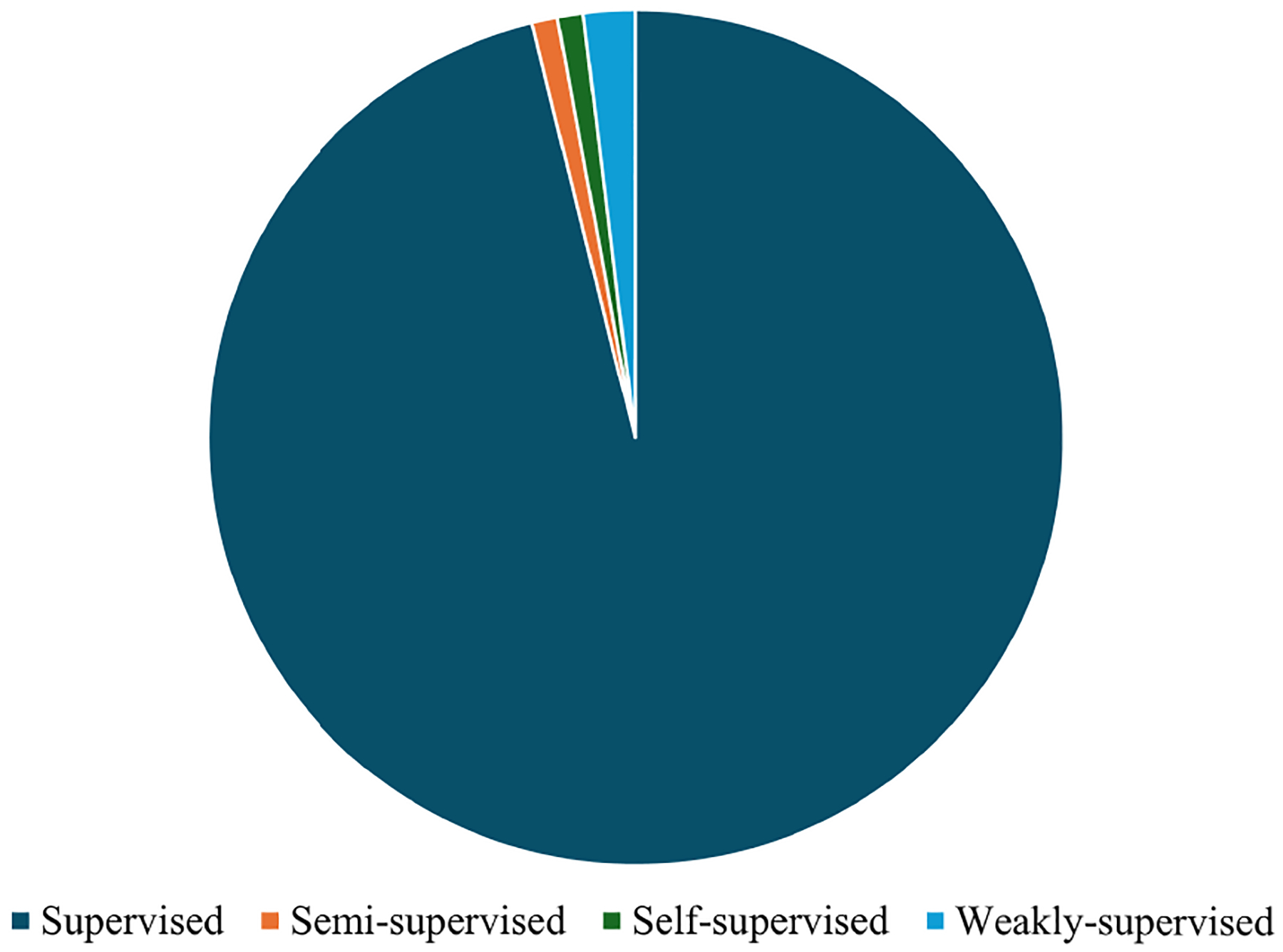
A breakdown of the various supervision paradigms used in the surveyed literature.

Due to the limited availability of public datasets [33] and challenges in acquiring high-quality annotated medical images [235], new learning strategies have been developed to improve segmentation with reduced reliance on annotations [236]. While supervised learning remains widely used for liver tumor segmentation (as shown in Figure 9) [237], it typically requires large amounts of labeled data, which may be impractical in many clinical settings [238]. To overcome these constraints, recent studies have explored novel learning paradigms that make better use of available data and improve generalizability. These include knowledge transfer, which leverages pretrained models and external datasets, and data-efficient learning, which aims to maintain strong performance with minimal annotated data [236]. A summary of key representative papers, including the segmentation methods employed, their evaluation performance on benchmark datasets, and the advantages and weaknesses of each method, is presented in Table 4.

### B. LEARNING STRATEGIES

#### 1) KNOWLEDGE TRANSFER METHODS

In medical image segmentation, prior knowledge from external datasets is often leveraged to enhance model performance. Transfer learning, for instance, involves finetuning pre-trained models on large datasets for specific downstream tasks and has been shown to improve liver lesion segmentation [29], [51], [98], [104]. In one study [185], a network was first pre-trained on the LiTS dataset and then fine-tuned on a larger private liver tumor dataset to further enhance segmentation accuracy. Some studies have adapted the VGG-16 as a transfer learning framework to improve liver tumor segmentation on small CT datasets [29], [104]. Defeudis et al. [239] compared liver lesion segmentation performance with and without transfer learning, highlighting its advantages.

Beyond transfer learning, other knowledge transfer techniques such as zero-shot, one-shot, and few-shot learning allow models to learn effectively from minimal labeled examples, thereby extending deep learning’s capabilities to smaller datasets [240], [241], [242], [243]. These strategies are particularly valuable in scenarios where annotated liver tumor datasets are limited, enabling models to generalize to new tasks with minimal supervision.

#### 2) DATA-EFFICIENT LEARNING METHODS

To minimize annotation burdens and enhance learning efficiency, various strategies have been developed [244], including active learning [245], semi-supervised learning [246], self-supervised learning [247], and weakly supervised learning [248].

Active learning optimizes the annotation process by selecting the most informative samples for manual labeling, thereby reducing the total number of labeled instances required for effective model training. A study comparing active learning strategies across MSD datasets showed that both random sampling and strided sampling serve as strong baselines, offering insights into their respective strengths and limitations [249].

Semi-supervised learning combines a small amount of labeled data with large volumes of unlabeled data, often using pseudo-labeling techniques to generate training annotations. For instance, dynamic thresholding and uncertainty rectification have been applied to create reliable pseudo-labels, improving liver tumor segmentation accuracy [189], [250]. Another study refined pseudo-labels using labeled data to enhance model robustness during training [251].

Self-supervised learning (SSL) further reduces the need for manual annotation by using unlabeled raw data to learn meaningful visual representations. One approach blends artificial tumors into normal organs and refines predictions through self-training [252]. Additionally, synthetic-to-real test-time training (SR-TTT) [73] utilizes SSL by incorporating two auxiliary networks—a generator and a reconstruction network—alongside the main segmentation model. These auxiliary networks help the model adapt to real-world data during inference by generating synthetic samples and reconstructing inputs, thereby improving segmentation performance.

Weakly supervised learning, on the other hand, trains models using noisy, partial, or imprecise labels such as image-level labels [253] or sparse annotations (e.g., bounding boxes, scribbles) [74], [145], [254], [255], [256], [257]. For example, [258] employed image-level labels to train an object presence classifier, and used a reinforcement learning–optimized policy to guide a sliding window across the 3D image volume. The classifier outputs were accumulated to construct a voxel-level probability map, which was subsequently converted into a tumor segmentation mask. In other study, Sun et al. [163] employed a teacher-student framework that utilized both a pixel-annotated (strong) dataset and a bounding box-annotated (weak) dataset. Similarly, DeepRecS [155] segmented tumor regions using response evaluation criteria in solid tumors (RECIST) measurements [259]. Additionally, Couinaud segment annotations [260], commonly used by radiologists, have been leveraged to enhance liver tumor segmentation [74]. Several studies have used weak labels to segment lesions [254], [255], [257], with one specifically focusing on liver tumor segmentation [256].

**FIGURE 10.**
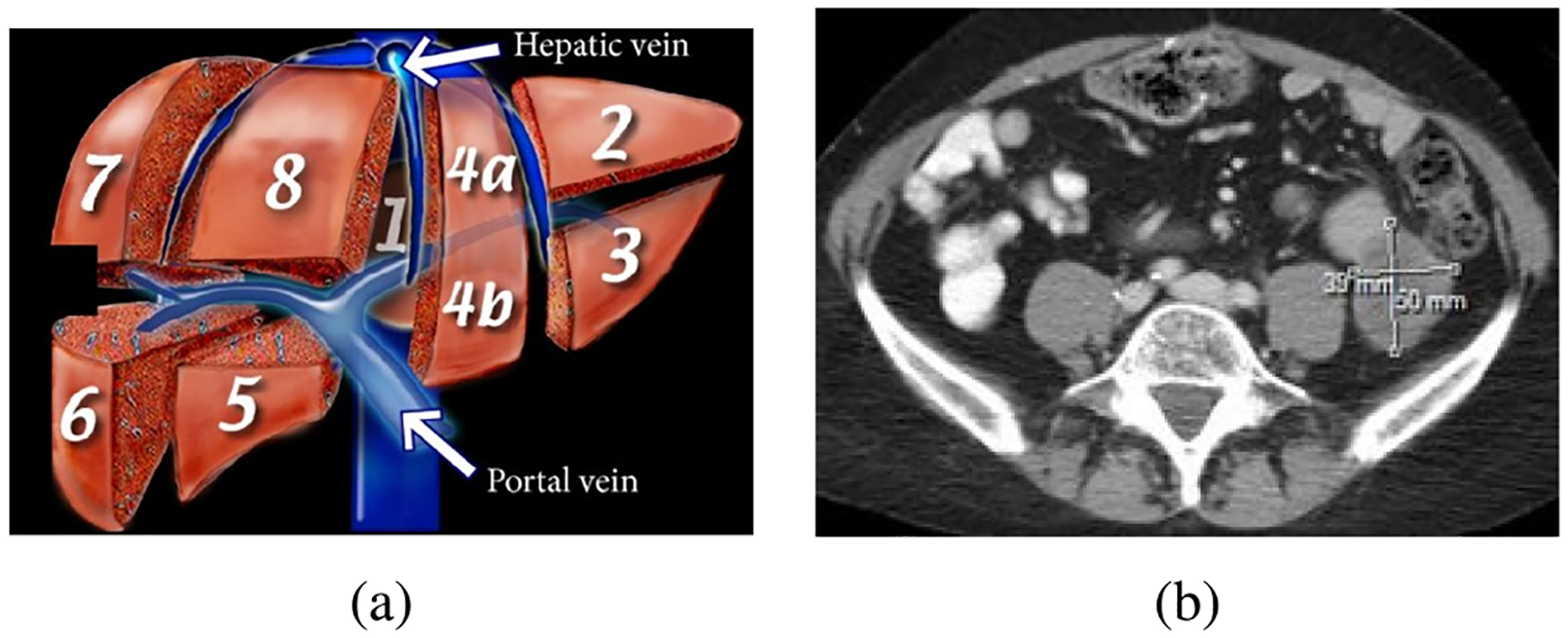
Liver anatomical reference and tumor annotation. (a) Couinaud classification of liver anatomy, which divides the liver into functional segments for localization. Figure adapted from [261] licensed under CC BY 4.0. (b) An example of a RECIST annotation indicating tumor boundaries on a liver image.

These data-efficient learning strategies help address annotation challenges while enhancing segmentation performance in liver tumor analysis. Figure 10a and Figure 10b depict the Couinaud segments and RECIST marks, respectively.

### C. COMPARATIVE ANALYSIS OF KEY TECHNIQUES

Initial deep learning models (e.g., [90], [91], [101]) eliminated the need for hand-crafted features by automatically learning local spatial dependencies. Although they performed reasonably well on small objects, they often failed to preserve structural integrity and produced imprecise segmentation maps. Architectures with shortcut connections and deeper layers, such as H-Dense U-Net [142] and the modified SegNet proposed in [115], improved feature reuse, enabled richer representations, and allowed relatively fast training; however, they still struggled to capture global context and often lacked boundary precision. Reference [155] incorporated multi-scale feature fusion, which enhanced boundary delineation by leveraging global context, though at the expense of increased architectural complexity and the risk of including irrelevant features. Lightweight designs [100], [143], further reduced the number of parameters and training time but generally sacrificed robustness when applied to heterogeneous tumors. More recent efforts have integrated attention mechanisms to focus on clinically relevant regions and features. For example, [182] introduced HANS-Net, which combines hyperbolic convolutions with adaptive temporal attention and a synaptic plasticity module. These components enhance hierarchical feature representation, enforce inter-slice consistency, and improve robustness, leading to more accurate liver and tumor segmentation, albeit with higher architectural complexity. A comparative summary of these categorized approaches is provided in Table 5.

### D. LOSS FUNCTION

A liver tumor segmentation model maps an input image x to a segmentation mask $\overset{ˆ}{y}$, which can be formalized as:

(1) $${\overset{ˆ}{y}}_{i}=f\left(x_{i}\right)$$

where $\overset{ˆ}{y}$ is a binary mask indicating the tumor region [106]. DL models adjust a large set of parameters θ during the training process to maximize the likelihood of the predicted mask $\overset{ˆ}{y}$ and the ground-truth mask y. The optimal set of parameters $\overset{‾}{\theta }$ is obtained by maximizing the log-likelihood over the training set [32]:

(2) $$\overset{‾}{\theta }=\text{arg}\;\underset{\theta }{\text{max}}\sum_{i=1}^{S}\text{log}\;P\left(y_{i}|x_{i},\theta \right)$$

where S denotes the total number of training samples. To achieve optimal segmentation, the loss between the predicted mask $\overset{ˆ}{y}$ and the ground-truth mask y must be minimized:

(3) $$\overset{‾}{\theta }=\text{arg}\;\underset{\theta }{\text{min}}\sum_{i=1}^{S}\text{loss}\left({\overset{ˆ}{y}}_{i}|y_{i}\right)=\text{arg}\;\underset{\theta }{\text{min}}\sum_{i=1}^{S}\text{loss}\left(f\left(x_{i}\right)|y_{i}\right)$$

Choosing an appropriate loss function is critical when training DL models. An effective loss function should be differentiable, handle class imbalance, be robust to outliers, and align with the task objectives [262]. In liver tumor segmentation research, various loss functions have been widely adopted to enhance segmentation accuracy and overall model performance [45], [237], [263].

#### 1) STANDARD LOSS FUNCTIONS IN TUMOR SEGMENTATION

##### a: CROSS ENTROPY (CE) LOSS

Semantic segmentation is often considered as a pixel-wise classification problem. In this context, minimizing the negative log-likelihoods of pixel-wise predictions is achieved by minimizing the CE loss [264].

(4) $${ℒ}_{CE}(y,p)=-\sum_{i=1}^{N}\sum_{p\in \Omega_{i}}\left[y_{i}\;\text{log}\left(p_{i}\right)+\left(1-y_{i}\right)\;\text{log}\left(1-p_{i}\right)\right]$$

where $\Omega_{i}$ denotes the set of all pixels in image i, and $p_{i}$ is the predicted probability for pixel i.

In liver tumor segmentation research, each pixel is typically classified as either part of a tumor or background. Binary cross-entropy (BCE) loss is widely used for this binary classification task [136]. However, due to the significant class imbalance between tumor regions and background, weighted cross-entropy (WCE) loss has been proposed to address this issue [265]. WCE applies pixel-wise or class-wise weighting schemes to assign greater importance to tumor pixels during training. For instance, some studies [47], [101] have applied foreground-background pixel weights to mitigate the effects of class imbalance.

##### b: DICE LOSS

Dice loss is derived from the Dice similarity coefficient (DSC), which is a widely used statistical measure for evaluating segmentation accuracy [121]. It measures the overlap between predicted segmentation and ground-truth mask:

(5) $${ℒ}_{\text{Dice}}(y,p)=1-\frac{1}{N}\sum_{i=1}^{N}\frac{2\sum_{p\in \Omega_{i}}y_{i}p_{i}+\varepsilon }{\sum_{p\in \Omega_{i}}y_{i}^{2}+\sum_{p\in \Omega_{i}}p_{i}^{2}+\varepsilon }$$

where N is the number of images. and ε is a small constant that prevents the denominator from dropping to zero.

##### c: INTERSECTION OVER UNION LOSS

The intersection over union (IoU) or Jaccard index, also known as the Jaccard similarity coefficient, measures the similarity between the predicted and ground truth masks [266]. The IoU loss function is formulated as:

(6) $${ℒ}_{\text{IoU}}(y,p)=1-\frac{1}{N}\sum_{i=1}^{N}\frac{\sum_{p\in \Omega_{i}}y_{i}p_{i}}{\sum_{p\in \Omega_{i}}\left(y_{i}+p_{i}-y_{i}p_{i}\right)}$$

The IoU loss function is effective for handling segmentation errors, particularly in cases where small regions like liver lesions are more likely to be misclassified.

##### d: TVERSKY LOSS

The Tversky loss, inspired by the Tversky index [267], is a generalized form of the Jaccard Index that allows for asymmetric weighting of false positives and false negatives. It is specifically designed to address class imbalance in segmentation tasks by adjusting the penalty applied to each type of error.

(7) $${ℒ}_{Tv}(y,p)=1-\frac{1}{N}\sum_{i=1}^{N}\frac{\sum_{p\in \Omega_{i}}y_{i}p_{i}}{\sum_{p\in \Omega_{i}}y_{i}p_{i}+\alpha y_{i}\left(1-p_{i}\right)+\beta \left(1-y_{i}\right)p_{i}}$$

where α and β are parameters that control the balance between false positives and false negatives. Typically, higher values of α penalize false negatives more, which is beneficial when tumor regions are small compared to the background [268].

##### e: FOCAL LOSS

Focal loss is a modified version of cross-entropy loss, designed to focus learning on hard-to-classify pixels by down-weighting well-classified examples [269]. This scaling mechanism reduces the impact of easy examples during training, enabling the model to prioritize learning from challenging examples more efficiently.

(8) $${ℒ}_{FL}(y,p)=-\alpha {\left(1-p_{i}\right)}^{\gamma }\;\text{log}\left(p_{i}\right)$$

Here, α is a balancing factor that adjusts the contribution of minority and majority classes in the data. γ is a focusing parameter that controls the emphasis on hard-to-classify examples. Higher values of γ reduce the impact of easy examples, allowing the model to concentrate on difficult cases. Focal loss is particularly useful in medical image segmentation, where small tumors are often underrepresented in training data [270].

#### 2) COMBINED LOSS FUNCTIONS FOR ENHANCED SEGMENTATION

To improve the accuracy of liver tumor segmentation, mitigate class imbalance in CT images, and enhance model generalization, hybrid loss functions—which combine multiple loss types—are widely employed in recent research [271]. By integrating complementary loss functions, models can leverage the strengths of each while compensating for their individual limitations.

One of the most common combinations in liver tumor segmentation is Dice loss with BCE. While Dice loss captures the overall segmentation quality by focusing on region-level overlap, BCE ensures pixel-wise classification precision, leading to a more stable and effective optimization process [159]. Several studies have combined these two into a single BCE + Dice hybrid loss, resulting in improved segmentation accuracy and training stability [100], [125], [159].

To further address the prevalent imbalance between foreground and background samples, [173] proposed integrating cross-entropy loss, Dice loss, and Focal Tversky loss. Another study adopted a two-stage loss strategy—employing BCE during early training for faster convergence, followed by Tversky loss to focus on hard negative samples, thereby reducing misclassification of background regions [272].

In another approach, a dual supervision scheme was introduced by combining Dice loss with Euclidean loss, ensuring both spatial consistency and pixel-wise accuracy in liver tumor segmentation [141]. Additionally, shape-aware losses such as boundary loss, first proposed by Kervadec et al. [273], have been effectively combined with Dice loss to improve sensitivity to tumor boundaries and fine details [195]. Similarly, Han et al. [124] proposed a composite loss function—Dice+CE+contour loss—to enhance tumor edge detection and segmentation precision.

Beyond standard hybrid loss formulations, deep supervision has emerged as an effective strategy for further refining segmentation performance. Unlike conventional methods that apply a loss function only at the final layer, deep supervision introduces auxiliary loss terms at intermediate layers, encouraging the model to learn meaningful hierarchical representations [274]. For instance, a cascaded segmentation network, CDS-Net, applied deep supervision to guide liver lesion segmentation within a single deep network architecture [275].

Another powerful technique is adversarial training, which introduces a discriminator to evaluate the realism of predicted segmentation masks. This additional supervision encourages the segmentation network to produce more realistic and coherent results [276]:

(9) $${ℒ}_{\text{total}}(y,p)=\lambda \cdot {ℒ}_{\text{pixel-wise}}(y,p)-{ℒ}_{\text{adv}}$$

where λ balances pixel-wise loss $\left(L_{\text{pixel-wise}}\right)$ and adversarial loss $\left(L_{\text{adv}}\right)$. Adversarial loss typically uses BCE to push predictions closer to ground truth distributions [277].

#### 3) MULTI-OBJECTIVE LOSS FUNCTIONS

Deep learning models for liver tumor segmentation have increasingly been extended to multi-task learning frameworks, where segmentation is performed alongside additional tasks such as lesion classification or boundary detection. These methods leverage shared feature representations to reduce false positives and improve overall efficiency.

Cascaded networks typically segment the liver first, followed by tumor segmentation, applying separate loss functions at each step [138], [218], [278]. However, cascade frameworks are not end-to-end; they require longer training times and introduce the risk of error propagation from liver segmentation to tumor segmentation [111].

To address this limitation, researchers have explored end-to-end joint segmentation models, where a single network simultaneously segments both the liver and liver tumors [111], [160], [197]. One such approach employed a dual U-Net architecture, with two parallel branches handling liver and tumor segmentation independently within the same network. This study found that similarity-based loss functions outperformed WCE loss [135].

Several studies have further expanded this approach by integrating segmentation and classification tasks within a unified model. For example, Y-Net [110] combined weighted cross-entropy for segmentation with categorical cross-entropy for classification, improving both segmentation accuracy and lesion categorization. Similarly, [167] used a joint loss function to simultaneously perform liver tumor segmentation and classification, while [147] extended this by incorporating detection, segmentation, and classification into a single pipeline. This model leveraged a weighted loss function combining cross-entropy loss and Dice loss for pixel-wise segmentation, BCE for patient-level classification, and L2 consistency loss to align lesion- and patient-level predictions.

Another promising direction involves boundary-aware segmentation models, which explicitly integrate edge detection into the segmentation task. Given the challenges posed by blurry tumor boundaries, a study by [279] introduced a three-channel model wherein separate channels were designed to learn liver segmentation, tumor segmentation, and edge prediction. This method improved segmentation accuracy, particularly near the boundaries.

Traditional models often treat segmentation and classification as independent tasks, leading to redundant computation due to separate feature extraction processes. This separation can reduce classification accuracy since segmentation-derived features are not fully utilized, and may lead to suboptimal feature representations, where classification models do not benefit from segmentation-guided learning. In contrast, multi-task learning frameworks provide a more unified approach by integrating segmentation and classification, allowing for shared feature representations that improve overall model performance and enhance feature learning [156].

### V. EVALUATION

#### A. SEGMENTATION ANNOTATIONS

Accurate annotations are essential for evaluating deep learning-based liver tumor segmentation models, yet obtaining high-quality ground truth is both costly and challenging. Expert-annotated datasets enable model training, with annotation methods ranging from semi-automatic techniques, where experts refine software-generated borders, to manual segmentation, in which lesions are traced using tools like ITK-SNAP [280], a widely used open-source annotation software. While manual annotation is commonly used and generally reliable, it is time-consuming and requires expert-level training [237], [281].

Annotation noise—resulting from data uncertainties and inter-observer variability—can significantly impact segmentation performance [282]. Variability among annotators, or even within the same expert over time, introduces inconsistencies. Metrics such as the Dice similarity coefficient (DSC) and Cohen’s Kappa [283] are commonly used to quantify inter-annotator agreement, providing a quantitative measure of annotation reliability. Due to the high cost of annotation, many datasets rely on a single annotator; however, multi-expert annotations, such as those in the LiTS17 dataset, often implement consensus mechanisms to resolve discrepancies [32].

Strategies for ground truth consolidation include bitwise operations [284], majority voting [285], shape averaging [286], [287], and statistical methods such as STAPLE [288] and SIMPLE [289], which weigh annotations based on annotator expertise. For example, the LiTS17 dataset was manually segmented by a radiologist with over three years of oncologic imaging experience, reviewed by three independent readers, and finalized by the most senior expert [33]. Such multi-expert validation enhances dataset reliability and ultimately improves the performance of segmentation models.

### B. EVALUATION METRICS

**FIGURE 11.**
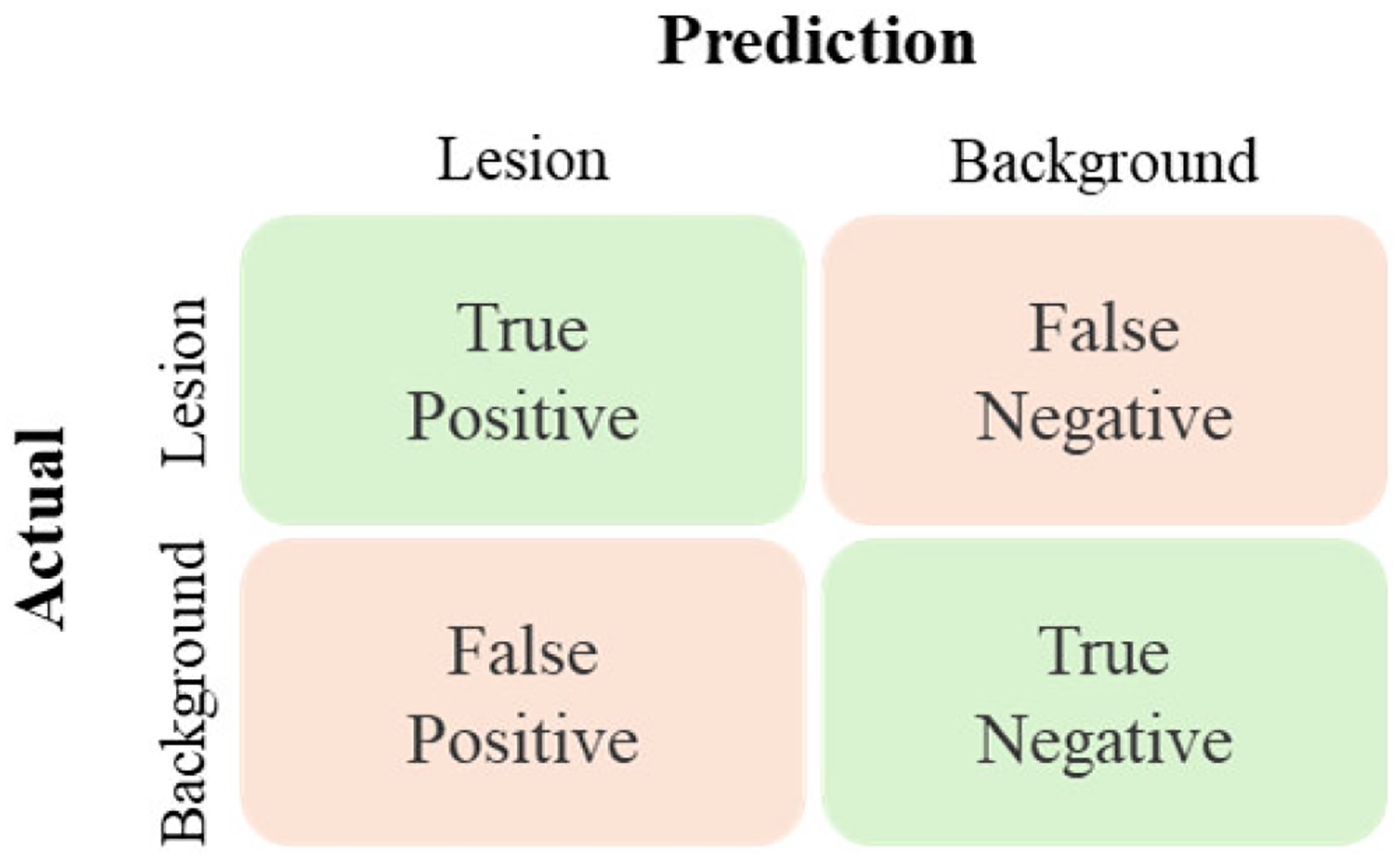
A confusion matrix for binary classification task.

Several metrics are widely used to evaluate the performance of medical image segmentation algorithms, particularly in liver tumor segmentation research [290]. These metrics quantitatively assess the agreement between predicted segmentations and ground truth annotations. Liver tumor segmentation can be framed as a classification problem, where the positive class represents the tumor lesion and the negative class represents the background, as illustrated in Figure 11.

For this classification task, a confusion matrix [291] is used, where TP, FN, FP, and TN represent true positives, false negatives, false positives, and true negatives, respectively. In addition to confusion matrix-based metrics, distance-based evaluation metrics are also employed to capture the spatial accuracy of the segmentation results. Table 6 provides a summary of key evaluation metrics commonly reported in the literature.

## VI. DISCUSSION

### A. CHALLENGES AND LIMITATIONS

While CT remains the most widely used modality for liver tumor segmentation due to its availability, high spatial resolution, and quantitative HU values, it has notable disadvantages compared with other modalities. MRI offers superior soft-tissue contrast and multiparametric imaging sequences, which improve delineation of small lesions, but it is more expensive, slower, and less accessible in routine practice. Notably, 4D MRI has been shown to yield fewer artifacts and more accurate motion assessment than 4D CT, thereby reducing uncertainty in target delineation for radiotherapy [292]. Ultrasound, particularly contrast-enhanced ultrasound (CEUS), offers portability, cost-effectiveness, real-time imaging, and higher temporal and in-plane spatial resolution than CT or MRI, with the added benefit of being radiation-free and associated with lower risk of adverse reactions. Nevertheless, CEUS is constrained by operator dependence, limited penetration in certain patients, lack of multiplanar capability, and reduced reproducibility [293]. Recent studies suggest that combining CT with CEUS or MRI can leverage the complementary strengths of these modalities—CT for structural and quantitative information, CEUS/MRI for functional or contrast sensitivity— to improve segmentation accuracy [45]. However, multi-modal approaches introduce additional challenges, including registration, increased computational cost, and harmonization across imaging protocols [45].

In liver tumor segmentation, data diversity and quality are critical for developing effective DL models [33]. A major limitation in this field is the scarcity of benchmark datasets with expert pixel-level annotations, which are essential for accurate tumor identification [45]. While traditional image augmentation techniques attempt to address this limitation by generating new data, they typically provide only minor modifications to existing samples, offering limited enhancement in data diversity [76]. Consequently, augmented data often retains the same underlying distribution, providing minimal variation and limited additional insight for the model.

Generative approaches, such as GAN-based synthesis, offer a more advanced solution. However, they are computationally intensive, require large amounts of real data for training, may be sensitive to inconsistent tumor masks and depend heavily on precise pixel-wise tumor masks [57], [65]. Similarly, heuristic methods struggle to accurately replicate complex tumor characteristics—such as shape, location, and texture—that are essential for capturing the detailed pathology of liver tumors [235]. As a result, achieving high-quality, diverse data for liver tumor synthesis remains a major challenge, limiting further improvements in segmentation accuracy.

The lack of diverse, annotated datasets can result in poor model generalizability, increased risk of overfitting, and domain shift issues when synthetic data is used for training [235]. Table 2 illustrates the scarcity of publicly available datasets with comprehensive annotations. These challenges highlight the importance of developing effective strategies to preserve the utility of synthetic data in enhancing model performance. While supervised learning continues to dominate training paradigms in DL-based segmentation (as shown in Figure 9) [294], alternative approaches— such as semi-supervised, self-supervised, weakly supervised, and unsupervised learning—remain underexplored and may provide promising avenues to address data limitations [236], [294].

The continued success of DL in medical image analysis depends significantly on appropriate learning paradigms (e.g., supervised vs. unsupervised) and the evolution of network architectures [295]. Architectural advancements—from basic CNNs to specialized models like U-Net—have played a pivotal role in improving segmentation performance [296]. Attention mechanisms and transformer-based architectures further enhanced global context modeling and introduced valuable inductive biases, boosting segmentation accuracy for complex medical images [173]. However, larger models capable of analyzing high-dimensional medical images often demand significant computational resources, posing accessibility barriers for researchers without high-end hardware [297]. This trade-off between performance and resource efficiency emphasizes the importance of optimizing both model architecture and computational cost.

Additionally, a recurring limitation in the literature is the lack of transparency regarding code, hyperparameter settings, and implementation details, which hampers reproducibility [298]. Promoting open sharing of models, code, and training settings can significantly contribute to community-wide progress and amplify scientific impact.

Beyond algorithmic performance, liver tumor segmentation carries important clinical implications. Accurate delineation of tumors enables precise estimation of tumor burden, which informs surgical planning and eligibility for liver transplantation [113]. In radiation oncology, reliable segmentation ensures accurate targeting of lesions while minimizing exposure to surrounding healthy tissue [12]. Segmentation results are also essential for monitoring treatment response under RECIST criteria and can support prognostic modeling by quantifying tumor morphology and heterogeneity [14]. Despite these benefits, clinical adoption remains limited, highlighting the need for models that are reproducible, stable, and generalizable across centers, as well as seamlessly integrated into existing clinical workflows. Privacy concerns surrounding patient data storage and inter-institutional sharing require robust solutions and regulatory frameworks that ensure security and compliance. Furthermore, developing standardized protocols for secure data exchange between medical institutions will be essential for multi-center validation and deployment. Another critical direction is the simplification of annotation processes, which remain a major bottleneck in training and validating segmentation models. Tools that streamline annotation and support radiologists more efficiently will facilitate smoother integration into clinical practice and encourage widespread adoption.

In summary, overcoming the challenges and limitations of deep learning techniques for liver segmentation is essential for their effective adoption in clinical settings. Key areas of focus should include managing data scarcity by utilizing more accessible datasets, enhancing the robustness of DL approaches, improving interpretability, refining uncertainty estimation, and considering ethical implications. These efforts will be essential for advancing and responsibly implementing deep learning models in liver segmentation.

### B. PROSPECTS FOR FURTHER INVESTIGATION

Deep learning has revolutionized medical image segmentation, particularly in the challenging domain of liver tumor analysis [299]. While numerous studies have demonstrated its effectiveness [296], several promising avenues remain underexplored.

First, leveraging state-of-the-art image generation techniques could significantly enhance segmentation performance [64]. The success of deep learning models largely depends on the availability of large, diverse, and accurately annotated datasets [76]. However, publicly available liver tumor datasets often suffer from limitations in terms of size, heterogeneity, and representativeness [33]. This underscores the importance of exploring advanced generative methods, which have the potential to substantially improve segmentation outcomes [64], [65]. In particular, diffusion probabilistic models represent a compelling direction for generating realistic synthetic data, thereby augmenting training datasets and improving model generalizability [300].

Second, integrating expert knowledge with deep features holds great promise for advancing segmentation accuracy [69]. Radiologists possess valuable domain-specific insights, enabling precise manual segmentation. However, this process is both time-consuming and subject to inter-observer variability [235], [288]. A hybrid approach—one that combines deep learning-derived features with handcrafted features reflecting anatomical priors (e.g., shape, texture, appearance)—could improve not only segmentation accuracy but also model interpretability [301]. Although some research has begun to explore this integration [302], further studies are needed to fully realize its potential.

Third, the automation of annotation using information from radiologists’ reports is an emerging area of interest. These reports—whether in free-text form or structured formats like the liver imaging reporting and data system (LI-RADS)—contain rich semantic information that could guide model training [303]. Previous work has investigated using Couinaud segment annotations and RECIST markers as weak supervision sources for segmentation [74], [155]. However, automatically converting narrative reports into accurate annotations using NLP remains a significant challenge [304].

Finally, leveraging existing medical images and annotations across multiple organs to improve the generalizability of models for liver tumor segmentation has recently emerged as a promising direction that warrants greater attention and has the potential to transform the field [305]. development of foundation models such as SAM and CLIP offers exciting opportunities for interactive, general-purpose medical image segmentation guided by natural language supervision [200], [203], [205]. When combined with large language models (LLMs), these systems could bridge the gap between image and text understanding, enabling more intuitive and flexible interactions with medical data.

In summary, future research in liver tumor segmentation should prioritize the development of more diverse and representative datasets [52], [76], the integration of expert-driven and deep features [301], the use of advanced generative models such as diffusion methods [68], the automation of annotation via NLP techniques [304], and the application of foundation models and large language models to bridge visual and textual data [200]. These advancements will play a crucial role in enhancing both the accuracy and clinical applicability of deep learning-based segmentation systems.

## VII. CONCLUSION

Medical image analysis heavily relies on segmentation—a fundamental task for identifying anatomical structures and pathological regions of interest. In this study, we conducted a comprehensive survey of DL architectures applied to liver tumor segmentation in CT images. Our investigation spans from early DL models to recent advances, highlighting the architectural evolution over time.

CNNs have emerged as the dominant architecture for both liver and liver tumor segmentation in 3D medical images. A common strategy adopted by researchers involves a two-step process: initially segmenting the liver, followed by tumor segmentation within the liver region defined as the ROI. While recent DL architectures have significantly improved tumor segmentation accuracy, further refinement is needed for practical clinical deployment.

Although automatic liver segmentation techniques have achieved performance levels comparable to human experts, liver tumor segmentation continues to pose major challenges. One of the primary obstacles is the limited availability of publicly accessible datasets with comprehensive tumor annotations. Broader access to such datasets would support the development and evaluation of more robust 3D DL architectures tailored for volumetric medical imaging. Moreover, insights derived from models trained on these datasets could inform the design of computer-aided detection (CAD) systems, enabling automated tumor identification with minimal human intervention.

## Figures and Tables

**TABLE 1.** Abbreviations frequently used in the text.

Abbreviation	Description
ASPP	Atrous Spatial Pyramid Pooling
BCE	Binary Cross Entropy
CE	Cross Entropy
CNN	Convolutional Neural Network
CT	Computed Tomography
DL	Deep Learning
FCN	Fully Convolutional Network
GAN	Generative Adversarial Network
IoU	Intersection over Union
LiTS	Liver Tumor Segmentation (Challenge)
MRI	Magnetic Resonance Imaging
RECIST	Response Evaluation Criteria in Solid Tumors
ROI	Region of Interest
SAM	Segment Anything Model

**TABLE 2.** Overview of public liver segmentation datasets.

Dataset	Year	Liver	Tumor	Vessels	Modality	CT Liver Dataset Size (Train/Test)	Provider
SLiver07	2007	✔	✘	✘	CE-CT	30 (20/10)	German Cancer Research Center
LTSC08	2008	✘	✔	✘	CT	30 (20/10)	Siemens
3D-IRCADb-01, 02	2010	✔	✔	✔	CE-CT	20 (15/5), 2 (N/A)	IRCAD
MIDAS	2010	✘	✔	✘	CT	4 (N/A)	IMAR
BTCV	2015	✔	✘	✘	CT	50 (30/20)	Vanderbilt University
CLEF2015	2015	✔	✘	✘	CT	60 (50/10)	BOǦAZIÇI University
VISCERAL	2016	✔	✘	✘	CT/CE-CT/MRI/CE-MRI	60 (40/20)	University of Geneva
TCGA-LIHC	2016	✔	✔	✘	CT/MR/PT	75 (N/A)	TCIA
LiTS	2017	✔	✔	✔	CT	201 (131/70)	Technical University of Munich
MSD	2018	✔	✔	✔	CE-CT	443 (303/140)	MICCAI
CHAOS	2019	✔	✘	✘	CT/MRI	40 (20/20)	DOKUZ Eylul University
Barts CRL	2024	✘	✔	✘	CE-CT	220	Barts Health NHS Trust

**TABLE 3.** Comparison of representative synthetic data generation methods, highlighting their key advantages and limitations in medical image analysis.

Generation method	Representative studies	Advantages	Disadvantages
Basic image manipulations	[44]	Simple and computationally inexpensive Reduce overfitting by introducing variation Do not require additional annotations Effective in small datasets to improve robustness	Cannot generate truly novel samples Limited in capturing complex tumor variations May not reflect clinical diversity of liver tumors Excessive augmentation may distort anatomy
Deep learning-based	[53]–[55], [61], [62], [65]–[67], [72]	Generate entirely new and diverse samples Synthesize realistic lesion appearances Enhance cross-institution generalization Flexible control over tumor properties	Require pixel-wise annotations Sensitive to annotation variability GANs may produce unrealistic artifacts Training instability (GANs) Computationally intensive, data hungry Limited clinical validation
Heuristic-based	[74]–[76]	Reduce manual annotation costs Controlled design of tumor features Enable pre-training with low annotations Improve segmentation with synthetic tumors Integrate clinical knowledge for realism	Early methods lacked spatial awareness May not capture tumor heterogeneity Limited generalization across organs Risk of bias from oversimplified rules Require expert validation for realism

**TABLE 4.** Summary of key representative studies on liver lesion segmentation.

Study	Method	Dataset	Performance	Advantages	Disadvantages	Year
[17]	Deep CNNs	Private	(80.06 ± 1.63) %	Outperformed ML-based segmentation methods; robust to inhomogeneous tumors with clear boundaries	small dataset; limited for fuzzy tumors	2015
[90]	Hierarchical CNN	LiTS	DSC:65.7 %	Robust to class imbalance	Limited accuracy in small lesions	2017
[91]	FCN and level-set	LiTS	DSC:71.8 %	Improved tumor boundary accuracy vs pure DL	Limited accuracy in fuzzy boundaries	2018
[92]	Global CNN + Patient-specific CNN	Private	VOE: (17.0 ± 11.6)%, ASSD: 2.0 mm	Improves over single CNNs; fully automatic with baseline mask	Requires baseline delineation; limited for rare/necrotic tumors	2018
[96]	FCN (VGG-19) + NMF refinement	LiTS, 3D-IRCADb	VOE: 0.13–1.04; ASD: 0.76–0.96 mm; Sens: 0.93–0.97; Spec: 0.96–0.99	Smooth boundary refinement	Weak on small low-contrast lesions	2017
[98]	Cascaded FCNs + 3D CRF	3D-IRCADb	DSC(liver): 94.73 %	High liver accuracy; good sensitivity	Limited dataset diversity; long training	2025
[100]	FCN (2D+3D hybrid)	LiTS, 3D-IRCADb	DSC(LiTS): 73.0%; DSC(3D-IRCADb): 94.1 %	Lightweight model; cross-dataset robustness; efficient	Lower accuracy than H-DenseUNet; limited by small datasets	2019
[101]	FCN (VGG-16) + 3D CRF	LiTS	DSC: 0.59	Reduces false positives via detector; leverages multi-slice context	Requires extra detector/CRF; modest lesion Dice	2017
[102]	Cascaded FCNs + 3D Dense CRF	3D-IRCADb	DSC: 82.3 %	Better spatial consistency; surpasses single U-Net; reasonably fast	Small cohort (15 tumors); 2D slice limitation; contrast sensitive	2016
[103]	Cascaded FCNs + 3D CRF	3D-IRCADb, MRI (private)	DSC(3D-IRCADb): (61 ± 25)%	Multi-center evaluation; CT+MRI generalization	Low lesion Dice; costly CRF tuning	2017
[104]	Dual CNN (AlexNet) + FCN	Private	Mean loc. error: 3.9 vox (~7.8 mm)	Robust 3D localization; multi-organ potential; simple fusion	Localization only (no segmentation); needs downstream segmenter	2016
[107]	U-Net 3+	Private	Higher Dice than U-Net++	Improved feature fusion; fewer parameters; reduced over-segmentation	No liver-tumor benchmark; organ-only validation	2020
[110]	U-Net + SE-ResNet encoder (joint)	Private + LiTS pretrain	DSC: 0.76, Acc: 0.86	Joint seg+cls improves both tasks; transfer learning helpful	Very small 2D dataset; limited to 3 lesion types	2020
[111]	Two sequential U-Nets (liver, tumor)	LiTS, private	Qualitative only (no Dice)	Joint liver-tumor pipeline; promising clinical robustness	No quantitative metrics; small single-center data	2019
[113]	Modified U-Net	3D-IRCADb	DSC: 75.37 %	Solid accuracy on small data; straightforward architecture	Limited external validation; weak on small lesions	2024
[114]	DenseNet-based cascaded FCN	LiTS	DSC: 62.5 %	Feature reuse via dense links; ROI cascade aids localization	HU-window sensitivity; 2D slice limitation	2017
[115]	Modified U-Net	LiTS, 3D-IRCADb	DSC(LiTS): 89.38%; DSC(3D-IRCADb): 69.80 %	Strong LiTS performance; efficient on downscaled inputs	Lower on 3D-IRCADb; resizing loses detail; limited data size	2022
[117]	Modified SegNet (VGG-16)	3D-IRCADb-01	Acc: 99.9 %	Very high pixel accuracy; memory-efficient; handles imbalance	Overestimates tumors; small dataset	2020
[120]	Hybrid Cascaded CompNet (2D+3D)	LiTS	DSC: 68.1 %	Improves small-lesion detection; hybrid efficiency	Threshold sensitivity; heavier than pure 2D; moderate Dice	2018
[123]	Three-stage Curriculum U-Net	LiTS	DPC: 82.2 %	Better small-lesion learning; faster inference	Multi-stage training; single-dataset validation	2020
[124]	2.5D Cascaded FCNs + 3D CRFs	LiTS, 3D-IRCADb	DSC(LiTS): 74.5%; DSC(3D-IRCADb): 68 %	Sharper boundaries; efficient vs full 3D; reduced false positives	Multi-stage pipeline; boundary-complexity sensitive	2021
[125]	Light 2.5D nnU-Net	LiTS	DSC: (81.4 ± 2.5)%	Near-3D nnU-Net accuracy with far fewer params; faster training	Slight Dice drop vs 3D nnU-Net; 2.5D context	2021
[127]	Lightweight U-Net	LiTS	DSC: 58.7%	Strong parameter efficiency; competitive for size	Lower accuracy than SOTA; weak on small lesions	2018
[128]	3D-DenseUNet	LiTS	DPC: 69.6 % GDSC: 80.7 %	Parameter-efficient depth; relatively low memory	Below hybrid SOTA; struggles with tiny lesions	2021
[131]	Deep CNN (2.5D)	LiTS	DSC: 0.67	LiTS Challenge winner; efficient trade-off of context/memory	Modest lesion Dice; heavy training; small-lesion gaps	2017
[132]	FC-ResNet	LiTS, EM, PROMISE12	DSC(LiTS): 0.711	End-to-end normalization; multi-modality strength	2D limitation; large model; moderate lesion Dice	2017
[133]	Cascaded Deep Residual Net	LiTS	DSC: 64.0 %	Multi-scale fusion; strong challenge placement	Modest Dice; long training; 2D limitation	2017
[134]	FED-Net (ResNet-50 + attention fusion)	LiTS	DPC: 65.0 %, GDSC:76.6 %	Attention/DUC recover detail; lightweight vs 3D	Below H-DenseUNet; limited 3D context	2019
[135]	Cascade U-ResNets + loss ensembles	LiTS	DSC: 70.1 %	Ensemble improves accuracy; efficient 2.5D design	Multi-model complexity; still below H-DenseUNet	2020
[152]	RIS-UNet (Residual–Inception–SE blocks)	LiTS, 3D-IRCADb	DSC: 82.6 %	Enhances multi-scale feature fusion via parallel convolutions + residual links + SE attention	Slightly heavy architecture; higher training cost	2024
[138]	3D MCG + FRN + ACM	LiTS, 3D-IRCADb	DPC(3D-IRCADb): 0.67, GDPC(3D-IRCADb): 0.764	Coarse-to-fine pipeline; better small/multiple lesion recall	Irregular boundaries; merges adjacent lesions; computationally heavy	2019
[140]	UNet++	LiTS	LiTS (liver) IoU: 82.9 % (~, DPC(liver): 0.90	Improved fine-detail fusion; supports pruning; consistent gains	Higher complexity; pruning may reduce accuracy; mainly 2D	2018
[141]	BS U-Net (dense+inception+dilated + bottleneck sup.)	LiTS	DPC: 56.9 %, GDSC: 75.1 %	Better anatomy preservation; fewer parameters than U-Net	Lower lesion Dice than H-DenseUNet; two-network training cost	2020
[142]	H-DenseUNet (2D+3D hybrid dense U-Net)	LiTS, 3D-IRCADb	DSC(LiTS): 72.2 %; DSC(3D-IRCADb): 81.2 %	Strong cross-dataset generalization; hybrid intra/inter-slice fusion	Heavy training/inference; cascaded workflow	2018
[146]	3D AH-Net (ResNet-50 + anisotropic blocks)	LiTS	GDSC: 83.4%, DPC: 63.4%	Effective 2D → 3D transfer; anisotropy-aware; better small-lesion focus	Below H-DenseUNet; two-step training; complex kernels	2018
[148]	VA Mask-RCNN (volumetric attention)	LiTS	DPC: 74.1 %	Improves small-lesion sensitivity; reduces FPs; uses 2.5D context	Still below top hybrids; higher GPU use	2019
[156]	modified UNet++ and DenseNet	Private	DSC: 71.9 %	Sharper edges via ASPP+fusion; clinical features aid classification	Small single-center set; manual cropping; moderate Dice	2022
[157]	Cascade 3D U-Net + attention modules	LiTS, 3D-IRCADb	DPC(LiTS): 66.5 %, GDSC(LiTS): 81.2%; DPC(3D-IRCADb): 71.9 %, GDSC(3D-IRCADb): 78.4%	Better boundaries via attention	Cascade complexity; tumor Dice below H-DenseUNet	2022
[158]	MDAU-Net (residual+attention)	LiTS, SLiver07	DSC(LiTS): 83.9 %	Strong edge preservation; consistent gains over U-Net variants	Multi-module complexity; heavier training; limited datasets	2023
[159]	CAFCT-Net (CNN+Transformer)	LiTS	DSC: 84.29 %	Integrates global+local context; improved boundary detail	2D slice evaluation; complex multi-module design	2024
[163]	Teacher-Student (O2L + localization branch)	LiTS	GDSC: 95.8%, DSC: 95.4%	Strong with fewer labels; better small-lesion detection	Two-stage training; small-lesion Dice still lags full supervision	2022
[166]	SFF-Net (side outputs + FFBs + 3D CRF)	LiTS	GDSC: 74.6 %, DPC: 59.2 %	Preserves spatial detail; reduced semantic gap; higher precision	Moderate case-wise Dice; heavier than vanilla U-Net; CRF dependency	2021
[168]	RA-UNet (3D residual attention U-Net)	LiTS, 3D-IRCADb	DPC(LiTS): 59.5 %, GDSC(LiTS): 79.5%; GDPC(3D-IRCADb): 83.0 %	Attention-enhanced 3D features; good external validation	Heavy 3D compute; moderate lesion Dice; multi-stage pipeline	2020
[182]	HANS-Net (Hyperbolic Conv + Adaptive Temporal Attention)	LiTS, 3D-IRCADb-01	DSC(LiTS): 89.84%, IoU: 88.09%, DSC(3D-IRCADb): 80.08 %, IoU 80.30 %	Strong cross-dataset generalization; captures hierarchical geometry (hyperbolic conv) and fine details; improves inter-slice consistency	Slightly lower tumor Dice on 3D-IRCADb; added complexity and inference time	2025
[175]	TransFusionNet (CNN+Transformer)	LiTS, 3D-IRCADb, private	IoU: 0.927 (~, DSC: 0.96	Joint tumor+vessel modeling; strong edge detail; deployable variant	Complex training; small private set; transfer reliance	2023
[176]	MFA-Net (U-Net + SCSE)	3D-IRCADb, LiTS	DSC(3D-IRCADb): 77.1%; DSC(LiTS): 67.1 %	Joint spatial+channel recalibration; improves over U-Net/++	Lower than Attention U-Net on LiTS; small datasets; 2D-only	2024
[185]	AC-Net (U-Net + attention)	LiTS, private	DSC: 90.0 %	Combines local+global context; robust CT performance; code available	Moderate compute/memory; limited modality scope	2024
[187]	CC-DenseUNet (DenseNet + CCA)	LiTS, 3D-IRCADb, private	DSC(LiTS): 74.1%; DSC(3D-IRCADb): 66.3%; DSC(private): 74.5 %	Consistent multi-dataset results; better gradient flow; transfer learning helps	Heavier GPU; slower than lightweight U-Nets	2023
[191]	T-MPEDNet (Transformer-aware Progressive Encoder-Decoder)	LiTS, 3D-IRCADb	DSC(LiTS): 89.1 %; DSC(3D-IRCADb): 83.3 %	Strong small-lesion performance; integrates global+local cues; refined boundaries	High complexity/memory; residual under-segmentation	2025
[193]	TD-Net (U-shaped CNN+Transformer skip)	LiTS, 3D-IRCADb	DPC(LiTS): 0.709, GDSC(LiTS): 0.832; DPC(3D-IRCADb): 0.682	Global context in skips; direction-guided boundaries; competitive vs CNN baselines	2D-only processing; higher GPU cost; below some 3D models	2023
[194]	DHT-Net (3D Transformer CNN hybrid)	LiTS, 3D-IRCADb	DPC(LiTS): 76.8%, GDSC(LiTS): 85%	Outperforms 2D/2.5D/3D baselines; sharper edges; robust across sets	Heavy computation/memory; needs larger dataset	2023
[197]	SWTR-UNet (ResNet + Swin bottleneck)	LiTS	DSC:(79 ± 25) %	Captures global+local context; cross-modality applicability (CT, MRI)	Very large model; slower inference; weaker on sub-cm lesions	2023
[198]	Improved SwinUNet	LiTS, 3D-IRCADb	DSC(LiTS): 76.14%; DSC(3D-IRCADb): 71.38 %	Captures global+local context; enhances salient features	Higher training/inference cost; complex multi-module design; still 2D-based	2025
[199]	Modified Tokenized MLP + Attention U-Net	LiTS	DSC: 72.1 %	Lightweight; fast inference; spatial-shift MLP improves feature interaction; attentions boost boundary segmentation	Slightly below top Dice; weak on tiny tumors	2025
[222]	ASLseg (SSL U-Net + SAM fine-tune + Adaptation Net)	LiTS	DSC: (76.16 ± 0.28) %	Effective use of unlabeled data; improved pseudo-labels; strong SSL baseline	Three-stage complexity; pseudo-label quality dependent; single-dataset study	2024
[228]	TPM-SAM (twin encoder + prompt gen + multi-layer decoder)	LiTS	DSC: 75.19%	Better boundary detail; depth+space correlation; beats several SAM baselines	Higher complexity; CT-only validation; transformer overhead	2024
[230]	RefSAM3D (3D SAM + cross-modal ref prompt)	LiTS	DSC: 80.10 %	3D spatial context; supports visual/text prompts; strong generalization	High GPU/memory; complex adapters; occasional under-segmentation	2024
[231]	DEAP-3DSAM (3D SAM + Dual Attention Prompter)	LiTS	DSC: 54.16 %	Fully automatic prompting; improved decoder features	Higher complexity/GPU; modest liver performance; weak on dispersed tiny tumors	2024

**TABLE 5.** Summary of deep learning-based segmentation models.

Category	Architectural categories	Advantages	Disadvantages
CNN/FCN	[90], [91], [101]	Strong local feature extraction Computationally efficient Widely validated as baseline	Limited global context Loss of fine boundaries Weak on large/complex tumors Sensitive to domain shift
U-Net and its variants	[51], [115], [121]–[123]	Preserves fine details via skip connections Strong performance on small datasets Widely adopted in medical imaging	Limited global context modeling High memory/computation in 3D variants Risk of overfitting on small datasets
Residual/Dense networks	[140]–[142]	Capture deeper hierarchical features Promote feature reuse (dense connections) Better convergence and stability	Architecturally complex High computational/memory demands Risk of overfitting with small data
Multi-scale feature fusion	[155], [157]	Combine local details with global context Robust to tumor size/shape variability Improve boundary delineation	Architecturally complex Longer training/inference time Risk of redundant features
Attention-based	[34], [176]	Focus on relevant tumor regions Improve boundary precision Enhance robustness in heterogeneous livers	Additional computational overhead May still miss long-range context Sensitive to noisy annotations Risk of overfitting with limited data
Self-attention and Transformers	[193]–[195]	Capture long-range dependencies Handle heterogeneous tumor patterns Scalable to large datasets	High computational requirements Dependence on large annotated cohorts Limited effectiveness on small datasets
Foundation models	[222], [223], [232]	Pretrained on large-scale data Strong cross-domain generalization Enable multi-tasking (e.g., segmentation + classification) Support zero/few-shot learning	Require extensive resources for fine-tuning Some rely on user input (e.g., SAM-based models) Limited clinical validation for liver tumors

**TABLE 6.** Overview of popular evaluation metrics used in segmentation tasks.

Metric	Equation	
Precision (PR)	$PR=\frac{TP}{TP+FP}$	(10)
Recall (RE) / Sensitivity (SE)	$RE=\frac{TP}{TP+FN}$	(11)
Specificity (SP)	$SP=\frac{TN}{TN+FP}$	(12)
Accuracy (AC)	$AC=\frac{TP+TN}{TP+TN+FP+FN}$	(13)
F1 Score (F1)	$F1=2\cdot \frac{PR\cdot RE}{PR+RE}$	(14)
Balanced Accuracy (BA)	$BA=\frac{SE+SP}{2}$	(15)
IoU / Jaccard Index (JI)	$\text{IoU}=\frac{\left|A\cap B\right|}{\left|A\cup B\right|}=\frac{TP}{TP+FP+FN}$	(16)
Where A is ground-truth and B is generated prediction.		
Global Dice Similarity Coefficient (GDSC)	$\text{GDSC}=\frac{2|A\cap B|}{\left|A|+|B\right|}=\frac{2TP}{2TP+FP+FN}$	(17)
Dice per Case (DPC)	$\text{DPC}=\frac{1}{N}\overset{N}{\underset{i=1}{\sum }}\frac{2\left|A_{i}\cap B_{i}\right|}{\left|A_{i}\right|+\left|B_{i}\right|}=\frac{1}{N}\overset{N}{\underset{i=1}{\sum }}\frac{2TP_{i}}{2TP_{i}+FP_{i}+FN_{i}}$	(18)
Volumetric Overlap Error (VOE)	$\text{VOE}=1-\frac{\left|A\cap B\right|}{\left|A\cup B\right|}=\frac{FP+FN}{TP+FP+FN}$	(19)
Relative Volumetric Difference (RVD)	$\text{RVD}(A,B)=\frac{\left|B|-|A\right|}{\left|A\right|}$	(20)
Average Symmetric Surface Distance (ASD)	$\text{ASD}(A,B)=\frac{1}{\left|S(A)|+|S(B)\right|}\left(\underset{s_{A}\in S(A)}{\sum }d\left(s_{A},S(B)\right)+\underset{s_{B}\in S(B)}{\sum }d\left(s_{B},S(A)\right)\right)$	(21)
$s_{A}$ denotes the distance of a single surface voxel from the set of surface voxels S(A).		
Root-Mean-Square Surface Distance (RMSD)	$\text{RMSD}(A,B)=\sqrt{\frac{1}{\left|S(A)|+|S(B)\right|}\left(\underset{s_{A}\in S(A)}{\sum }d^{2}\left(s_{A},S(B)\right)+\underset{s_{B}\in S(B)}{\sum }d^{2}\left(s_{B},S(A)\right)\right)}$	(22)
Maximum Symmetric Surface Distance (MSD) / Hausdorff Distance (HD)	$\text{MSD}(A,B)=\text{max}\left\{\underset{s_{A}\in S(A)}{\text{max}}d\left(s_{A},S(B)\right),\underset{s_{B}\in S(B)}{\text{max}}d\left(s_{B},S(A)\right)\right\}$	(23)

## References

[1] Siegel RL, Kratzer TB, Giaquinto AN, Sung H, and Jemal A, “Cancer statistics,” CA, Cancer J. Clinicians, vol. 75, no. 1, pp. 10–45, Jan. 2025. 10.3322/caac.21871 PMC11745215 39817679

[2] Chou R, Cuevas C, Fu R, Devine B, Wasson N, Ginsburg A, Zakher B, Pappas M, Graham E, and Sullivan SD, “The diagnostic value of serum Glypican-3 in the diagnosis of hepatocellular carcinoma: A systematic review and meta-analysis,” Int. J. Health Med. Res, vol. 2, no. 10, pp. 697–711, Sep. 2015. 10.7326/M14-2509 25984845

[3] Fass L, “Imaging and cancer: A review,” Mol. Oncol, vol. 2, no. 2, pp. 115–152, 2008. 19383333 10.1016/j.molonc.2008.04.001 PMC5527766

[4] Fox SH, Tanenbaum L, Ackelsberg SM, He H, Hsieh J, and Hu H, “Future directions in CT technology,” Neuroimaging Clinics North Amer, vol. 8, no. 3, pp. 497–513, 1998. 9673309

[5] Yeh BM, FitzGerald PF, Edic PM, Lambert JW, Colborn RE, Marino ME, Evans PM, Roberts JC, Wang ZJ, Wong MJ, and Bonitatibus PJ, “Opportunities for new CT contrast agents to maximize the diagnostic potential of emerging spectral CT technologies,” Adv. Drug Del. Rev, vol. 113, pp. 201–222, Apr. 2017. 10.1016/j.addr.2016.09.001 PMC5344792 27620496

[6] Stoker J, “Magnetic resonance imaging and the acute abdomen,” Brit. J. Surgery, vol. 95, no. 10, pp. 1193–1194, Sep. 2008. 10.1002/bjs.6378 18763236

[7] Rashidian N, Alseidi A, and Kirks RC, “Cancers metastatic to the liver,” Surgical Clinics North Amer, vol. 100, no. 3, pp. 551–563, Jun. 2020. 10.1016/j.suc.2020.02.005 32402300

[8] Schwartz LH, Litière S, de Vries E, Ford R, Gwyther S, Mandrekar S, Shankar L, Bogaerts J, Chen A, Dancey J, Hayes W, Hodi FS, Hoekstra OS, Huang EP, Lin N, Liu Y, Therasse P, Wolchok JD, and Seymour L, “RECIST 1.1—Update and clarification: From the RECIST committee,” Eur. J. Cancer, vol. 62, pp. 132–137, Jul. 2016. 27189322 10.1016/j.ejca.2016.03.081 PMC5737828

[9] Radtke A, Nadalin S, Sotiropoulos GC, Molmenti EP, Schroeder T, Valentin-Gamazo C, Lang H, Bockhorn M, Peitgen HO, Broelsch CE, and Malagó M, “Computer-assisted operative planning in adult living donor liver transplantation: A new way to resolve the dilemma of the middle hepatic vein,” World J. Surgery, vol. 31, no. 1, pp. 175–185, Jan. 2007. 10.1007/s00268-005-0718-1 17180479

[10] Meinzer H-P, Thorn M, and Cárdenas CE, “Computerized planning of liver surgery—An overview,” Comput. Graph, vol. 26, no. 4, pp. 569–576, Aug. 2002.

[11] Zhou YJ, Tang Y, Liu SJ, Zeng PH, Qu L, Jing QC, and Yin WJ, “Radiation-induced liver disease: Beyond DNA damage,” Cell Cycle, vol. 22, no. 5, pp. 506–526, Mar. 2023. 36214587 10.1080/15384101.2022.2131163 PMC9928481

[12] Wang K and Tepper JE, “Radiation therapy-associated toxicity: Etiology, management, and prevention,” CA, Cancer J. Clinicians, vol. 71, no. 5, pp. 437–454, 2021. 10.3322/caac.21689 34255347

[13] Zhang W, Wang X, Jiang R, Hou J, Mu X, Li G, and Sun B, “Effect of tumor size on cancer-specific survival in small hepatocellular carcinoma,” Mayo Clinic Proc, vol. 90, no. 9, pp. 1187–1195, Sep. 2015. 10.1016/j.mayocp.2015.06.018 26231292

[14] Shehta A, Elsabbagh AM, Medhat M, Farouk A, Monier A, Said R, Salah T, Elshobari M, Fouad A, and Elghawalby AN, “Impact of tumor size on the outcomes of hepatic resection for hepatocellular carcinoma: A retrospective study,” BMC Surgery, vol. 24, no. 1, p. 7, Jan. 2024. 38172802 10.1186/s12893-023-02296-w PMC10765776

[15] Chen Z, Zheng H, Zeng W, Liu M, and Chen Y, “Prognostic analysis on different tumor sizes for 14634 hepatocellular carcinoma patients,” Eur. J. Cancer Care, vol. 2023, pp. 1–13, May 2023.

[16] Li M-J, Xie S, Teng Y-X, Ma L, Li L-Q, Xiang B-D, and Zhong J-H, “Comparison of survival rates as predicted by total tumor volume or tumor burden score in patients with hepatocellular carcinoma concurrent with fatty liver disease and hepatitis B virus,” Expert Rev. Gastroenterology Hepatology, vol. 17, no. 5, pp. 499–507, May 2023. 10.1080/17474124.2023.2196403 36975382

[17] Li W, Jia F, and Hu Q, “Automatic segmentation of liver tumor in CT images with deep convolutional neural networks,” J. Comput. Commun, vol. 3, no. 11, pp. 146–151, 2015.

[18] Baâzaoui A, Barhoumi W, Ahmed A, and Zagrouba E, “Semi-automated segmentation of single and multiple tumors in liver CT images using entropy-based fuzzy region growing,” IRBM, vol. 38, no. 2, pp. 98–108, Apr. 2017.

[19] Zhang Y, Yang J, Liu Y, Tian J, Wang S, Zhong C, Shi Z, Zhang Y, and He Z, “Decoupled pyramid correlation network for liver tumor segmentation from CT images,” Med. Phys, vol. 49, no. 11, pp. 7207–7221, Nov. 2022. 35620834 10.1002/mp.15723

[20] Li J and Niu Y, “Dual encoding DDS-UNet liver tumour segmentation based on multi-scale deep and shallow feature fusion,” IET Image Process., vol. 18, no. 5, pp. 1189–1199, Apr. 2024.

[21] Ma J, He Y, Li F, Han L, You C, and Wang B, “Segment anything in medical images,” Nature Commun, vol. 15, no. 1, p. 654, Jan. 2024. 38253604 10.1038/s41467-024-44824-z PMC10803759

[22] Masood S, Sharif M, Masood A, Yasmin M, and Raza M, “A survey on medical image segmentation,” Current Med. Imag. Rev, vol. 11, no. 1, pp. 3–14, Apr. 2015.

[23] Withey DJ and Koles ZJ, “A review of medical image segmentation: Methods and available software,” Int. J. Bioelectromagnetism, vol. 10, no. 3, pp. 125–148, 2008.

[24] Wang R, Lei T, Cui R, Zhang B, Meng H, and Nandi AK, “Medical image segmentation using deep learning: A survey,” IET Image Process., vol. 16, no. 5, pp. 1243–1267, Apr. 2022.

[25] Kumar SS, Moni RS, and Rajeesh J, “Automatic liver and lesion segmentation: A primary step in diagnosis of liver diseases,” Signal, Image Video Process., vol. 7, no. 1, pp. 163–172, Jan. 2013.

[26] Bi L, Peng Y, Feng DD, and Kim J, “Semi-Mamba: Improving medical image segmentation via semi-automatic mamba network,” in Advances in Computer Graphics. Cham, Switzerland: Springer, pp. 166–178.

[27] Ciresan D, Giusti A, Gambardella L, and Schmidhuber J, “Deep neural networks segment neuronal membranes in electron microscopy images,” in Proc. Adv. Neural Inf. Process. Syst, vol. 25, 2012, pp. 1–7.

[28] Ahn SH, Yeo AU, Kim KH, Kim C, Goh Y, Cho S, Lee SB, Lim YK, Kim H, Shin D, Kim T, Kim TH, Youn SH, Oh ES, and Jeong JH, “Comparative clinical evaluation of atlas and deep-learning-based auto-segmentation of organ structures in liver cancer,” Radiat. Oncol, vol. 14, no. 1, Dec. 2019, Art. no. 213. 10.1186/s13014-019-1392-z PMC6880380 31775825

[29] Shelhamer E, Long J, and Darrell T, “Fully convolutional networks for semantic segmentation,” in Proc. IEEE Conf. Comput. Vis. Pattern Recognit. (CVPR), Jun. 2014, pp. 3431–3440. 10.1109/TPAMI.2016.2572683 27244717

[30] Wang S, Li C, Wang R, Liu Z, Wang M, Tan H, Wu Y, Liu X, Sun H, Yang R, Liu X, Chen J, Zhou H, Ben Ayed I, and Zheng H, “Annotation-efficient deep learning for automatic medical image segmentation,” Nature Commun, vol. 12, no. 1, p. 5915, Oct. 2021. 34625565 10.1038/s41467-021-26216-9 PMC8501087

[31] Buslaev A, Iglovikov VI, Khvedchenya E, Parinov A, Druzhinin M, and Kalinin AA, “Albumentations: Fast and flexible image augmentations,” Information, vol. 11, no. 2, p. 125, Feb. 2020.

[32] Mirikharaji Z, Abhishek K, Bissoto A, Barata C, Avila S, Valle E, Celebi ME, and Hamarneh G, “A survey on deep learning for skin lesion segmentation,” Med. Image Anal, vol. 88, Aug. 2023, Art. no. 102863. 10.1016/j.media.2023.102863 37343323

[33] Bilic P , “The liver tumor segmentation benchmark (LiTS),” Med. Image Anal, vol. 84, Feb. 2022, Art. no. 102680. 10.1016/j.media.2022.102680 PMC10631490 36481607

[34] Hettihewa K, Kobchaisawat T, Tanpowpong N, and Chalidabhongse TH, “MANet: A multi-attention network for automatic liver tumor segmentation in computed tomography (CT) imaging,” Sci. Rep, vol. 13, no. 1, Nov. 2023, Art. no. 20098. 10.1038/s41598-023-46580-4 PMC10654423 37973987

[35] Heimann T , “Comparison and evaluation of methods for liver segmentation from CT datasets,” IEEE Trans. Med. Imag, vol. 28, no. 8, pp. 1251–1265, Aug. 2009. 10.1109/TMI.2009.2013851 19211338

[36] Landman B, Xu Z, Igelsias J, Styner M, Langerak T, and Klein A, “MICCAI multi-atlas labeling beyond the cranial vault-workshop and challenge,” in Proc. MICCAI, vol. 5, 2015, p. 12.

[37] Jimenez-del-Toro O , “Cloud-based evaluation of anatomical structure segmentation and landmark detection algorithms: VISCERAL anatomy benchmarks,” IEEE Trans. Med. Imag, vol. 35, no. 11, pp. 2459–2475, Nov. 2016. 10.1109/TMI.2016.2578680 27305669

[38] Kavur AE , “CHAOS challenge–combined (CT-MR) healthy abdominal organ segmentation,” Med. Image Anal, vol. 69, Apr. 2021, Art. no. 101950. 10.1016/j.media.2020.101950 33421920

[39] Deng X and Du G, “3D segmentation in the clinic: A grand challenge II-liver tumor segmentation,” in Proc. MICCAI Workshop, 2008, pp. 406–423.

[40] Soler L, Hostettler A, Agnus V, Charnoz A, Fasquel J-B, Moreau J, Osswald A-B, Bouhadjar M, and Marescaux J. (2010). 3D Image Reconstruction for Comparison of Algorithm Database. [Online]. Available: https://www.ircad.fr/research/data-sets/liver-segmentation-3d-ircadb-01

[41] Erickson BJ, Kirk S, Lee Y, Bathe O, Kearns M, Gerdes C, Rieger-Christ K, and Lemmerman J, “The Cancer genome atlas liver hepatocellular carcinoma collection (TCGA-LIHC)(version 5),” SEER Program, Nat. Cancer Inst (NIH), Bethesda, MD, USA, Tech. Rep., 2016. [Online]. Available: 10.7937/K9/TCIA.2016.IMMQW8UQ

[42] Simpson AL , “A large annotated medical image dataset for the development and evaluation of segmentation algorithms,” 2019, arXiv:1902.09063.

[43] Bashir U, Wang C, Smillie R, Rayabat Khan AK, Ahmed HT, Ordidge K, Power N, Gerlinger M, Slabaugh G, and Zhang Q, “Barts CRL dataset,” Zenodo, Oct. 2024, doi: 10.5281/zenodo.14004841.

[44] Shorten C and Khoshgoftaar TM, “A survey on image data augmentation for deep learning,” J. Big Data, vol. 6, no. 1, pp. 1–48, Dec. 2019.

[45] Wu C, Chen Q, Wang H, Guan Y, Mian Z, Huang C, Ruan C, Song Q, Jiang H, Pan J, and Li X, “A review of deep learning approaches for multimodal image segmentation of liver cancer,” J. Appl. Clin. Med. Phys, vol. 25, no. 12, p. 14540, Dec. 2024. 10.1002/acm2.14540 PMC11633801 39374312

[46] Sizikova E, Badal A, Delfino JG, Lago M, Nelson B, Saharkhiz N, Sahiner B, Zamzmi G, and Badano A, “Synthetic data in radiological imaging: Current state and future outlook,” BJR|Artif. Intell, vol. 1, no. 1, p. 007, Mar. 2024. 10.1093/bjrai/ubae007 PMC13045695 42064396

[47] Arulappan A and Thankaraj ABR, “Liver tumor segmentation using a new asymmetrical dilated convolutional semantic segmentation network in CT images,” Int. J. Imag. Syst. Technol, vol. 32, no. 3, pp. 815–830, May 2022.

[48] Ouhmich F, Agnus V, Noblet V, Heitz F, and Pessaux P, “Liver tissue segmentation in multiphase CT scans using cascaded convolutional neural networks,” Int. J. Comput. Assist. Radiol. Surgery, vol. 14, no. 8, pp. 1–10, Aug. 2019. 10.1007/s11548-019-01989-z 31041697

[49] Valle E, Fornaciali M, Menegola A, Tavares J, Vasques Bittencourt F, Li LT, and Avila S, “Data, depth, and design: Learning reliable models for skin lesion analysis,” Neurocomputing, vol. 383, pp. 303–313, Mar. 2020.

[50] Wang G, Li W, Aertsen M, Deprest J, Ourselin S, and Vercauteren T, “Aleatoric uncertainty estimation with test-time augmentation for medical image segmentation with convolutional neural networks,” Neurocomputing, vol. 338, pp. 34–45, Apr. 2019. 10.1016/j.neucom.2019.01.103 PMC6783308 31595105

[51] Pattwakkar VN, Kamath S, Nanjundappa MK, and Kadavigere R, “Automatic liver tumor segmentation on multiphase computed tomography volume using SegNet deep neural network and k-means clustering,” Int. J. Imag. Syst. Technol, vol. 33, no. 2, pp. 729–745, Mar. 2023.

[52] Goyal M and Mahmoud QH, “A systematic review of synthetic data generation techniques using generative AI,” Electronics, vol. 13, no. 17, p. 3509, Sep. 2024.

[53] Dong Y, Deng Z, Pang T, Su H, and Zhu J, “Adversarial distributional training for robust deep learning,” 2020, arXiv:2002.05999.

[54] Moosavi-Dezfooli S-M, Fawzi A, and Frossard P, “DeepFool: A simple and accurate method to fool deep neural networks,” in Proc. IEEE Conf. Comput. Vis. Pattern Recognit. (CVPR), Jun. 2016, pp. 2574–2582.

[55] Chen L, Song H, Wang C, Cui Y, Yang J, Hu X, and Zhang L, “Liver tumor segmentation in CT volumes using an adversarial densely connected network,” BMC Bioinf, vol. 20, no. S16, p. 587, Dec. 2019, doi: 10.1186/s12859-019-3069-x. PMC6886252 31787071

[56] Goodfellow I, Pouget-Abadie J, Mirza M, Xu B, Warde-Farley D, Ozair S, Courville A, and Bengio Y, “Generative adversarial networks,” Commun. ACM, vol. 63, no. 11, pp. 139–144, 2020.

[57] Yi X, Walia E, and Babyn P, “Generative adversarial network in medical imaging: A review,” Med. Image Anal, vol. 58, Dec. 2019, Art. no. 101552. 10.1016/j.media.2019.101552 31521965

[58] Yang D, Xu D, Zhou SK, Georgescu B, Chen M, Grbic S, Metaxas D, and Comaniciu D, “Automatic liver segmentation using an adversarial image-to-image network,” 2017, arXiv:1707.08037.

[59] Chen Y, Li S, Yang S, and Luo W, “Liver segmentation in ct images with adversarial learning,” in Intelligent Computing Theories and Application. Cham, Switzerland: Springer, 2022, pp. 470–480.

[60] Zheng H, Lin L, Hu H, Zhang Q, Chen Q, Iwamoto Y, Han X, Chen Y-W, Tong R, and Wu J, “Semi-supervised segmentation of liver using adversarial learning with deep atlas prior,” in Proc. Med. Image Comput. Comput. Assist. Intervent, 2019, pp. 148–156.

[61] Liao J, Wang H, Gu H, and Cai Y, “Liver tumor segmentation method combining multi-axis attention and conditional generative adversarial networks,” PLoS ONE, vol. 19, no. 12, Dec. 2024, Art. no. e0312105. 10.1371/journal.pone.0312105 PMC11614272 39625955

[62] Biswas A, Maity SP, Banik R, Bhattacharya P, and Debbarma J, “GAN-driven liver tumor segmentation: Enhancing accuracy in biomedical imaging,” Social Netw. Comput. Sci, vol. 5, no. 5, p. 652, Jun. 2024, doi: 10.1007/s42979-024-02991-2.

[63] Frid-Adar M, Klang E, Amitai MM, Goldberger J, and Greenspan H, “Synthetic data augmentation using GAN for improved liver lesion classification,” in Proc. IEEE 15th Int. Symp. Biomed. Imag. (ISBI), Apr. 2018, pp. 289–293.

[64] Frid-Adar M, Diamant I, Klang E, Amitai M, Goldberger J, and Greenspan H, “GAN-based synthetic medical image augmentation for increased CNN performance in liver lesion classification,” Neurocomputing, vol. 321, pp. 321–331, Dec. 2018. [Online]. Available: https://www.sciencedirect.com/science/article/pii/S0925231218310749

[65] Jin Q, Cui H, Sun C, Meng Z, and Su R, “Free-form tumor synthesis in computed tomography images via richer generative adversarial network,” Knowl.-Based Syst, vol. 218, Apr. 2021, Art. no. 106753. [Online]. Available: https://www.sciencedirect.com/science/article/pii/S0950705121000162

[66] Liu Y, Yang F, and Yang Y, “Free-form lesion synthesis using a partial convolution generative adversarial network for enhanced deep learning liver tumor segmentation,” 2022, arXiv:2206.09065. 10.1002/acm2.13927 PMC10113707 36800255

[67] Zheng Z, Wang M, Fan C, Wang C, He X, and He X, “Light&fast generative adversarial network for high-fidelity CT image synthesis of liver tumor,” Comput. Methods Programs Biomed, vol. 254, Sep. 2024, Art. no. 108252. [Online]. Available: https://www.sciencedirect.com/science/article/pii/S0169260724002475 10.1016/j.cmpb.2024.108252 38843572

[68] Al Nomaan Nafi A, Hossain MA, Rifat RH, Uz Zaman MM, Ahsan MM, and Raman S, “Diffusion-based approaches in medical image generation and analysis,” 2024, arXiv:2412.16860.

[69] Kazerouni A, Aghdam EK, Heidari M, Azad R, Fayyaz M, Hacihaliloglu I, and Merhof D, “Diffusion models in medical imaging: A comprehensive survey,” Med. Image Anal, vol. 88, Aug. 2023, Art. no. 102846. [Online]. Available: https://www.sciencedirect.com/science/article/pii/S1361841523001068 10.1016/j.media.2023.102846 37295311

[70] Li X, Ren Y, Jin X, Lan C, Wang X, Zeng W, Wang X, and Chen Z, “Diffusion models for image restoration and enhancement: A comprehensive survey,” 2023, arXiv:2308.09388.

[71] Chen Q, Chen X, Song H, Xiong Z, Yuille A, Wei C, and Zhou Z, “Towards generalizable tumor synthesis,” 2024, arXiv:2402.19470.

[72] Liu Z, Ma C, She W, and Xie M, “Biomedical image segmentation using denoising diffusion probabilistic models: A comprehensive review and analysis,” Appl. Sci, vol. 14, no. 2, p. 632, Jan. 2024.

[73] Lyu F, Ye M, Ma AJ, Yip TC, Wong GL, and Yuen PC, “Learning from synthetic CT images via test-time training for liver tumor segmentation,” IEEE Trans. Med. Imag, vol. 41, no. 9, pp. 2510–2520, Sep. 2022. 10.1109/TMI.2022.3166230 35404812

[74] Lyu F, Ma AJ, Yip TC, Wong GL, and Yuen PC, “Weakly supervised liver tumor segmentation using Couinaud segment annotation,” IEEE Trans. Med. Imag, vol. 41, no. 5, pp. 1138–1149, May 2022. 10.1109/TMI.2021.3132905 34871168

[75] Hu Q, Xiao J, Chen Y, Sun S, Chen J-N, Yuille A, and Zhou Z, “Synthetic tumors make AI segment tumors better,” 2022, arXiv:2210.14845.

[76] Hu Q, Chen Y, Xiao J, Sun S, Chen J, Yuille A, and Zhou Z, “Label-free liver tumor segmentation,” in Proc. CVPR, Jun. 2023, pp. 7422–7432.

[77] Nazir N, Sarwar A, and Saini BS, “Recent developments in denoising medical images using deep learning: An overview of models, techniques, and challenges,” Micron, vol. 180, May 2024, Art. no. 103615. 10.1016/j.micron.2024.103615 38471391

[78] Boas FE and Fleischmann D, “CT artifacts: Causes and reduction techniques,” Imag. Med, vol. 4, no. 2, pp. 229–240, Apr. 2012.

[79] Sadia RT, Chen J, and Zhang J, “CT image denoising methods for image quality improvement and radiation dose reduction,” J. Appl. Clin. Med. Phys, vol. 25, no. 2, p. 14270, Feb. 2024. 10.1002/acm2.14270 PMC10860577 38240466

[80] Yugander P and Reddy GR, “Liver tumor segmentation in noisy CT images using distance regularized level set evolution based on fuzzy C-means clustering,” in Proc. 2nd IEEE Int. Conf. Recent Trends Electron., Inf. Commun. Technol. (RTEICT), May 2017, pp. 1530–1534.

[81] Huang Q, Ding H, Wang X, and Wang G, “Robust extraction for low-contrast liver tumors using modified adaptive likelihood estimation,” Int. J. Comput. Assist. Radiol. Surgery, vol. 13, no. 10, pp. 1565–1578, Oct. 2018. 10.1007/s11548-018-1820-9 29987741

[82] Lei Y, Niu C, Zhang J, Wang G, and Shan H, “CT image denoising and deblurring with deep learning: Current status and perspectives,” IEEE Trans. Radiat. Plasma Med. Sci, vol. 8, no. 2, pp. 153–172, Feb. 2024.

[83] Chen H, Zhang Y, Zhang W, Liao P, Li K, Zhou J, and Wang G, “Low-dose CT denoising with convolutional neural network,” in Proc. IEEE 14th Int. Symp. Biomed. Imag. (ISBI), Feb. 2017, pp. 143–146.

[84] Huang Z, Zhang J, Zhang Y, and Shan H, “DU-GAN: Generative adversarial networks with dual-domain U-Net-based discriminators for low-dose CT denoising,” IEEE Trans. Instrum. Meas, vol. 71, pp. 1–12, 2022.

[85] Missert A, Leng S, Yu L, and McCollough C, “Noise subtraction for low-dose ct images using a deep convolutional neural network,” in Proc. 5th Int. Conf. Image Formation X-Ray Comput. Tomogr, Jun. 2023, pp. 115–123.

[86] Zhang Z, Yu L, Liang X, Zhao W, and Xing L, “TransCT: Dual-path transformer for low dose computed tomography,” in Proc. Int. Conf. Med. Image Comput. Comput.-Assist. Intervent, 2022, pp. 55–64.

[87] Sattari MA, Zonouri SA, Salimi A, Izadi S, Rezaei AR, Ghezelbash Z, Hayati M, Seifi M, and Ekhteraei M, “Liver margin segmentation in abdominal CT images using U-Net and detectron2: Annotated dataset for deep learning models,” Sci. Rep, vol. 15, no. 1, p. 8721, Mar. 2025. 40082561 10.1038/s41598-025-92423-9 PMC11906767

[88] Anh LQ, Loc PX, and Ha LM, “Impact of image denoising techniques on CNN-based liver vessel segmentation using synthesis low-dose contrast enhanced CT images,” REV J. Electron. Commun, vol. 12, nos. 3–4, pp. 71–80, Feb. 2023.

[89] Rayed ME, Islam SMS, Niha SI, Jim JR, Kabir MM, and Mridha MF, “Deep learning for medical image segmentation: State-of-the-art advancements and challenges,” Informat. Med. Unlocked, vol. 47, Jan. 2024, Art. no. 101504.

[90] Yuan Y, “Hierarchical convolutional-deconvolutional neural networks for automatic liver and tumor segmentation,” 2017, arXiv:1710.04540.

[91] Zhang Y, Jiang B, Wu J, Ji D, Liu Y, Chen Y, Wu EX, and Tang X, “Deep learning initialized and gradient enhanced level-set based segmentation for liver tumor from CT images,” IEEE Access, vol. 8, pp. 76056–76068, 2020.

[92] Vivanti R, Joskowicz L, Lev-Cohain N, Ephrat A, and Sosna J, “Patient-specific and global convolutional neural networks for robust automatic liver tumor delineation in follow-up CT studies,” Med. Biol. Eng. Comput, vol. 56, no. 9, pp. 1699–1713, Sep. 2018. 29524116 10.1007/s11517-018-1803-6

[93] Aghamohammadi A, Ranjbarzadeh R, Naiemi F, Mogharrebi M, Dorosti S, and Bendechache M, “TPCNN: Two-path convolutional neural network for tumor and liver segmentation in CT images using a novel encoding approach,” Expert Syst. Appl, vol. 183, Nov. 2021, Art. no. 115406.

[94] Takahashi S, Sakaguchi Y, Kouno N, Takasawa K, Ishizu K, Akagi Y, Aoyama R, Teraya N, Bolatkan A, Shinkai N, Machino H, Kobayashi K, Asada K, Komatsu M, Kaneko S, Sugiyama M, and Hamamoto R, “Comparison of vision transformers and convolutional neural networks in medical image analysis: A systematic review,” J. Med. Syst, vol. 48, no. 1, p. 84, Sep. 2024. 39264388 10.1007/s10916-024-02105-8 PMC11393140

[95] Kang K and Wang X, “Fully convolutional neural networks for crowd segmentation,” 2014, arXiv:1411.4464.

[96] Zheng S, Fang B, Li L, Gao M, Wang Y, and Peng K, “Automatic liver lesion segmentation in CT combining fully convolutional networks and non-negative matrix factorization,” in Proc. BIVPCS/POCUS@MICCAI, 2017, pp. 44–51.

[97] Becker M and Magnenat-Thalmann N, Deformable Models in Medical Image Segmentation. Cham, Switzerland: Springer, 2014.

[98] Sun C, Guo S, Zhang H, Li J, Chen M, Ma S, Jin L, Liu X, Li X, and Qian X, “Automatic segmentation of liver tumors from multiphase contrast-enhanced CT images based on FCNs,” Artif. Intell. Med, vol. 83, pp. 58–66, Nov. 2017. 28347562 10.1016/j.artmed.2017.03.008

[99] Zhang Y, Wu J, Jiang B, Ji D, Chen Y, Wu E, and Tang X, “Deep learning and unsupervised fuzzy C-means based level-set segmentation for liver tumor,” in Proc. IEEE 17th Int. Symp. Biomed. Imag. (ISBI), Oct. 2020, pp. 1193–1196.

[100] Zhang J, Xie Y, Zhang P, Chen H, Xia Y, and Shen C, “Light-weight hybrid convolutional network for liver tumor segmentation,” in Proc. Int. Joint Conf. Artif. Intell, 2019, pp. 4271–4277.

[101] Bellver M, Maninis K-K, Pont-Tuset J, Giro-i-Nieto X, Torres J, and Van Gool L, “Detection-aided liver lesion segmentation using deep learning,” 2017, arXiv:1711.11069.

[102] Christ PF, Elshaer MEA, Ettlinger F, Tatavarty S, Bickel M, Bilic P, Rempfler M, Armbruster M, Hofmann F, D’Anastasi M, Sommer WH, Ahmadi S, and Menze B, “Automatic liver and lesion segmentation in CT using cascaded fully convolutional neural networks and 3D conditional random fields,” in Proc. Int. Conf. Med. Image Comput. Comput.-Assisted Intervent, 2016, pp. 415–423.

[103] Christ PF, Ettlinger F, Grün F, Ezzeldin M Elshaera A, Lipkova J, Schlecht S, Ahmaddy F, Tatavarty S, Bickel M, Bilic P, Rempfler M, Hofmann F, Anastasi MD, Ahmadi S-A, Kaissis G, Holch J, Sommer W, Braren R, Heinemann V, and Menze B, “Automatic liver and tumor segmentation of CT and MRI volumes using cascaded fully convolutional neural networks,” 2017, arXiv:1702.05970.

[104] Ben-Cohen A, Diamant I, Klang E, Amitai MM, and Greenspan H, “Fully convolutional network for liver segmentation and lesions detection,” in Proc. LABELS/DLMIA@MICCAI, 2016, pp. 77–85.

[105] Liu X, Song L, Liu S, and Zhang Y, “A review of deep-learning-based medical image segmentation methods,” Sustainability, vol. 13, no. 3, p. 1224, Jan. 2021.

[106] Ronneberger O, Fischer P, and Brox T, “U-Net: Convolutional networks for biomedical image segmentation,” 2015, arXiv:1505.04597.

[107] Huang H, Lin L, Tong R, Hu H, Zhang Q, Iwamoto Y, Han X, Chen Y, and Wu J, “UNet 3+: A full-scale connected UNet for medical image segmentation,” in Proc. ICASSP, Feb. 2020, pp. 1055–1059.

[108] Giannini V, Defeudis A, Rosati S, Cappello G, Vassallo L, Mazzetti S, Panić J, Regge D, and Balestra G, “Deep learning to segment liver metastases on CT images: Impact on a radiomics method to predict response to chemotherapy,” in Proc. IEEE Int. Symp. Med. Meas. Appl. (MeMeA), Jun. 2020, pp. 1–5.

[109] Gowda Y and Manjunath RV, “Automatic liver tumor classification using UNet70 a deep learning model,” J. Liver Transplantation, vol. 18, May 2025, Art. no. 100260.

[110] Heker M and Greenspan H, “Joint liver lesion segmentation and classification via transfer learning,” 2020, arXiv:2004.12352.

[111] Gruber N, Antholzer S, Jaschke W, Kremser C, and Haltmeier M, “A joint deep learning approach for automated liver and tumor segmentation,” in Proc. 13th Int. Conf. Sampling Theory Appl. (SampTA), Jul. 2019, pp. 1–5.

[112] Chlebus G, Meine H, Hendrik Moltz J, and Schenk A, “Neural network-based automatic liver tumor segmentation with random forest-based candidate filtering,” 2017, arXiv:1706.00842.

[113] Manjunath RV and Gowda NY, “Automated segmentation of liver tumors from computed tomographic scans,” J. Liver Transplantation, vol. 15, Aug. 2024, Art. no. 100232.

[114] Chaitanya Kaluva K, Khened M, Kori A, and Krishnamurthi G, “2D-densely connected convolution neural networks for automatic liver and tumor segmentation,” 2018, arXiv:1802.02182.

[115] Manjunath RV and Kwadiki K, “Modified U-NET on CT images for automatic segmentation of liver and its tumor,” Biomed. Eng. Adv, vol. 4, Dec. 2022, Art. no. 100043.

[116] Isobe S and Arai S, “Deep convolutional encoder–decoder network with model uncertainty for semantic segmentation,” in Proc. IEEE Int. Conf. Innov. Intell. Syst. Appl. (INISTA), Jul. 2017, pp. 365–370.

[117] Almotairi S, Kareem G, Aouf M, Almutairi B, and Salem MA-M, “Liver tumor segmentation in CT scans using modified SegNet,” Sensors, vol. 20, no. 5, p. 1516, Mar. 2020. 32164153 10.3390/s20051516 PMC7085510

[118] Wardhana G, Naghibi H, Sirmacek B, and Abayazid M, “Toward reliable automatic liver and tumor segmentation using convolutional neural network based on 2.5D models,” Int. J. Comput. Assist. Radiol. Surgery, vol. 16, no. 1, pp. 41–51, Jan. 2021. 10.1007/s11548-020-02292-y PMC7822806 33219906

[119] Dey R and Hong Y, “CompNet: Complementary segmentation network for brain MRI extraction,” 2018, arXiv:1804.00521.

[120] Dey R and Hong Y, “Hybrid cascaded neural network for liver lesion segmentation,” in Proc. IEEE 17th Int. Symp. Biomed. Imag. (ISBI), Apr. 2020, pp. 1173–1177.

[121] Milletari F, Navab N, and Ahmadi S-A, “V-net: Fully convolutional neural networks for volumetric medical image segmentation,” in Proc. 4th Int. Conf. 3D Vis. (3DV), Oct. 2016, pp. 565–571.

[122] Çiçek Ö, Abdulkadir A, Lienkamp SS, Brox T, and Ronneberger O, “3D U-Net: Learning dense volumetric segmentation from sparse annotation,” in Proc. Int. Conf. Med. Image Comput. Comput.-Assisted Intervent, 2016, pp. 424–432.

[123] Li H, Liu X, Boumaraf S, Liu W, Gong X, and Ma X, “A new three-stage curriculum learning approach for deep network based liver tumor segmentation,” in Proc. Int. Joint Conf. Neural Netw. (IJCNN), Jul. 2020, pp. 1–6.

[124] Han Y, Li X, Wang B, and Wang L, “Boundary loss-based 2.5D fully convolutional neural networks approach for segmentation: A case study of the liver and tumor on computed tomography,” Algorithms, vol. 14, no. 5, p. 144, Apr. 2021.

[125] Zhao Z, Ma Z, Liu Y, Zeng Z, and Chow PK, “Multi-slice dense-sparse learning for efficient liver and tumor segmentation,” in Proc. 43rd Annu. Int. Conf. IEEE Eng. Med. Biol. Soc. (EMBC), Nov. 2021, pp. 3582–3585. 10.1109/EMBC46164.2021.9629698 34892013

[126] Zhang C, Hua Q, Chu Y, and Wang P, “Liver tumor segmentation using 2.5D UV-Net with multi-scale convolution,” Comput. Biol. Med, vol. 133, Jun. 2021, Art. no. 104424. 10.1016/j.compbiomed.2021.104424 33984683

[127] Pandey RK, Vasan A, and Ramakrishnan AG, “Segmentation of liver lesions with reduced complexity deep models,” 2018, arXiv:1805.09233.

[128] Alalwan N, Abozeid A, ElHabshy AA, and Alzahrani A, “Efficient 3D deep learning model for medical image semantic segmentation,” Alexandria Eng. J, vol. 60, no. 1, pp. 1231–1239, Feb. 2021.

[129] He K, Zhang X, Ren S, and Sun J, “Deep residual learning for image recognition,” in Proc. IEEE Conf. Comput. Vis. Pattern Recognit. (CVPR), Jun. 2015, pp. 770–778.

[130] Selvaraj A and Nithiyaraj E, “CEDRNN: A convolutional encoder–decoder residual neural network for liver tumour segmentation,” Neural Process. Lett, vol. 55, no. 2, pp. 1605–1624, Apr. 2023.

[131] Han X, “Automatic liver lesion segmentation using a deep convolutional neural network method,” 2017, arXiv:1704.07239.

[132] Drozdzal M, Chartrand G, Vorontsov E, Shakeri M, Di Jorio L, Tang A, Romero A, Bengio Y, Pal C, and Kadoury S, “Learning normalized inputs for iterative estimation in medical image segmentation,” Med. Image Anal, vol. 44, pp. 1–13, Feb. 2018. 29169029 10.1016/j.media.2017.11.005

[133] Bi L, Kim J, Kumar A, and Feng D, “Automatic liver lesion detection using cascaded deep residual networks,” 2017, arXiv:1704.02703.

[134] Chen X, Zhang R, and Yan P, “Feature fusion encoder decoder network for automatic liver lesion segmentation,” in Proc. IEEE 16th Int. Symp. Biomed. Imag. (ISBI), Apr. 2019, pp. 430–433.

[135] Xi X-F, Wang L, Sheng VS, Cui Z, Fu B, and Hu F, “Cascade U-ResNets for simultaneous liver and lesion segmentation,” IEEE Access, vol. 8, pp. 68944–68952, 2020.

[136] Chen Y, Zheng C, Hu F, Zhou T, Feng L, Xu G, Yi Z, and Zhang X, “Efficient two-step liver and tumour segmentation on abdominal CT via deep learning and a conditional random field,” Comput. Biol. Med, vol. 150, Nov. 2022, Art. no. 106076. 10.1016/j.compbiomed.2022.106076 36137320

[137] Anil BC and Dayananda P, “Automatic liver tumor segmentation based on multi-level deep convolutional networks and fractal residual network,” IETE J. Res, vol. 69, no. 4, pp. 1925–1933, May 2023.

[138] Bai Z, Jiang H, Li S, and Yao Y-D, “Liver tumor segmentation based on multi-scale candidate generation and fractal residual network,” IEEE Access, vol. 7, pp. 82122–82133, 2019.

[139] Huang G, Liu Z, Van Der Maaten L, and Weinberger KQ, “Densely connected convolutional networks,” in Proc. IEEE Conf. Comput. Vis. Pattern Recognit. (CVPR), Jul. 2017, pp. 2261–2269.

[140] Zhou Z, Siddiquee MMR, Tajbakhsh N, and Liang J, “UNet++: A nested U-Net architecture for medical image segmentation,” in Proc.4th Int. Workshop Deep Learn. Med. Image Anal. Multimodal Learn. Clin. Decis. Support, vol. 11045, 2018, pp. 3–11. 10.1007/978-3-030-00889-5_1 PMC7329239 32613207

[141] Song LI, Geoffrey KF, and Kaijian HE, “Bottleneck feature supervised U-Net for pixel-wise liver and tumor segmentation,” Expert Syst. Appl, vol. 145, May 2020, Art. no. 113131.

[142] Li X, Chen H, Qi X, Dou Q, Fu C-W, and Heng P-A, “H-DenseUNet: Hybrid densely connected UNet for liver and tumor segmentation from CT volumes,” IEEE Trans. Med. Imag, vol. 37, no. 12, pp. 2663–2674, Dec. 2018. 10.1109/TMI.2018.2845918 29994201

[143] Chen L-C, Zhu Y, Papandreou G, Schroff F, and Adam H, “Encoder–decoder with atrous separable convolution for semantic image segmentation,” in Proc. Eur. Conf. Comput. Vis. (ECCV), 2018, pp. 833–851.

[144] Salpea N, Tzouveli P, and Kollias D, “Medical image segmentation: A review of modern architectures,” in Proc. Eur. Conf. Comput. Vis Cham, Switzerland: Springer, pp. 691–708.

[145] Fan M, Liu H, Zhu Z, Jiao C, and Gou S, “Weakly supervised liver tumor segmentation based on anchor box and adversarial complementary learning,” in Intelligence Science IV. Cham, Switzerland: Springer, pp. 68–75.

[146] Liu S, Xu D, Zhou SK, Pauly O, Grbic S, Mertelmeier T, Wicklein J, Jerebko A, Cai W, and Comaniciu D, “3D anisotropic hybrid network: Transferring convolutional features from 2D images to 3D anisotropic volumes,” in Proc. MICCAI, Sep. 2018, pp. 851–858.

[147] Yan K, Yin X, Xia Y, Wang F, Wang S, Gao Y, Yao J, Li C, Bai X, Zhou J, Zhang L, Lü L, and Shi Y, “Liver tumor screening and diagnosis in CT with pixel-lesion-patient network,” in Proc. Int. Conf. Med. Image Comput. Comput.-Assisted Intervent, 2023, pp. 72–82.

[148] Wang X, Han S, Chen Y, Gao D, and Vasconcelos N, “Volumetric attention for 3D medical image segmentation and detection,” 2020, arXiv:2004.01997.

[149] Li Y, Zou B, and Liu Q, “A deep attention network via high-resolution representation for liver and liver tumor segmentation,” Biocybernetics Biomed. Eng, vol. 41, no. 4, pp. 1518–1532, Oct. 2021.

[150] Zhang Y, Tian J, Zhong C, Zhang Y, Shi Z, and He Z, “DARN: Deep attentive refinement network for liver tumor segmentation from 3D CT volume,” in Proc. 25th Int. Conf. Pattern Recognit. (ICPR), 2021, pp. 7796–7803.

[151] Ilesanmi AE, Ilesanmi T, Idowu OP, Torigian DA, and Udupa JK, “Organ segmentation from computed tomography images using the 3D convolutional neural network: A systematic review,” Int. J. Multimedia Inf. Retr, vol. 11, no. 3, pp. 315–331, Sep. 2022.

[152] Wan Y, Zhang L, and Wang M, “RIS-UNet: A multi-level hierarchical framework for liver tumor segmentation in CT images,” Entropy, vol. 27, no. 7, p. 735, Jul. 2025. 40724451 10.3390/e27070735 PMC12295949

[153] Yu F and Koltun V, “Multi-scale context aggregation by dilated convolutions,” 2015, arXiv:1511.07122.

[154] Chen L-C, Papandreou G, Kokkinos I, Murphy K, and Yuille AL, “DeepLab: Semantic image segmentation with deep convolutional nets, Atrous convolution, and fully connected CRFs,” IEEE Trans. Pattern Anal. Mach. Intell, vol. 40, no. 4, pp. 834–848, Apr. 2018. 28463186 10.1109/TPAMI.2017.2699184

[155] Zhang Y, Peng C, Peng L, Xu Y, Lin L, Tong R, Peng Z, Mao X, Hu H, Chen Y-W, and Li J, “DeepRecS: From RECIST diameters to precise liver tumor segmentation,” IEEE J. Biomed. Health Informat, vol. 26, no. 2, pp. 614–625, Feb. 2022. 10.1109/JBHI.2021.3091900 34161249

[156] Peng Q, Yan Y, Qian L, Suo S, Guo Y, Xu J, and Wang Y, “Liver tumor segmentation and classification using FLAS-UNet++and an improved DenseNet,” Technol. Health Care, vol. 30, no. 6, pp. 1475–1487, Nov. 2022. 35661035 10.3233/THC-213655

[157] Wu Y, Shen H, Tan Y, and Shi Y, “Automatic liver tumor segmentation used the cascade multi-scale attention architecture method based on 3D U-Net,” Int. J. Comput. Assist. Radiol. Surgery, vol. 17, no. 10, pp. 1915–1922, Jun. 2022. 10.1007/s11548-022-02653-9 35672595

[158] Ma J, Xia M, Ma Z, and Jiu Z, “MDAU-Net: A liver and liver tumor segmentation method combining an attention mechanism and multi-scale features,” Appl. Sci, vol. 13, no. 18, p. 10443, Sep. 2023.

[159] Kang M, Ting C-M, Fung Ting F, and Phan R, “CAFCT-Net: A CNN-transformer hybrid network with contextual and attentional feature fusion for liver tumor segmentation,” 2024, arXiv:2401.16886.

[160] Wang H, Wang Z-M, Cui X-T, and Li L, “TDS-U-Net: Automatic liver and tumor separate segmentation of CT volumes using attention gates1,” J. Intell. Fuzzy Syst, vol. 44, no. 6, pp. 8817–8825, Jun. 2023.

[161] Mitrea D, Timu V, Mocan C-M, Nedevschi S, Florian A-V, Socaciu M, and Badea R, “Liver tumor segmentation from computed tomography images through convolutional neural networks,” in Proc. 9th Int. Conf. Syst. Informat. (ICSAI), Dec. 2023, pp. 1–6.

[162] Xie Y, Yang B, Guan Q, Zhang J, Wu Q, and Xia Y, “Attention mechanisms in medical image segmentation: A survey,” 2023, arXiv:2305.17937.

[163] Sun L, Wu J, Ding X, Huang Y, Chen Z, Wang G, and Yu Y, “A teacher–student framework for liver and tumor segmentation under mixed supervision from abdominal CT scans,” Neural Comput. Appl, vol. 34, no. 19, pp. 16547–16561, Oct. 2022.

[164] Vaswani A, Shazeer N, Parmar N, Uszkoreit J, Jones L, Gomez AN, Kaiser Ł, and Polosukhin I, “Attention is all you need,” in Proc. Adv. Neural Inf. Process. Syst, vol. 30, 2022, pp. 5998–6008.

[165] Guo M-H, Xu T-X, Liu J, Liu Z-N, Jiang P-T, Mu T, Zhang S-H, Martin RR, Cheng M-M, and Hu S, “Attention mechanisms in computer vision: A survey,” Comput. Vis. Media, vol. 8, no. 3, pp. 331–368, 2022.

[166] Liu T, Liu J, Ma Y, He J, Han J, Ding X, and Chen C-T, “Spatial feature fusion convolutional network for liver and liver tumor segmentation from CT images,” Med. Phys, vol. 48, no. 1, pp. 264–272, Jan. 2021. 33159809 10.1002/mp.14585

[167] Saumiya S and Franklin SW, “Unified automated deep learning framework for segmentation and classification of liver tumors,” J. Supercomput, vol. 80, no. 2, pp. 2347–2380, Jan. 2024.

[168] Jin Q, Meng Z, Sun C, Cui H, and Su R, “RA-UNet: A hybrid deep attention-aware network to extract liver and tumor in CT scans,” Frontiers Bioengineering Biotechnol, vol. 8, Dec. 2020, Art. no. 605132. 10.3389/fbioe.2020.605132 PMC7785874 33425871

[169] Bibi A and Khan MS, “Attention convolutional U-Net for automatic liver tumor segmentation,” in Proc. Int. Conf. Frontiers Inf. Technol. (FIT), Dec. 2021, pp. 102–107.

[170] Li Z, Zhang H, Li Z, and Ren Z, “Residual-attention UNet++: A nested residual-attention U-Net for medical image segmentation,” Appl. Sci, vol. 12, no. 14, p. 7149, Jul. 2022.

[171] Chen P-H, Huang C-H, Hung S-K, Chen L-C, Hsieh H-L, Chiou W-Y, Lee M-S, Lin H-Y, and Liu W-M, “Attention-LSTM fused U-Net architecture for organ segmentation in CT images,” in Proc. Int. Symp. Comput., Consum. Control (IS3C), Nov. 2020, pp. 304–307.

[172] Oktay O, Schlemper J, Le Folgoc L, Lee M, Heinrich M, Misawa K, Mori K, McDonagh S, Hammerla NY, Kainz B, Glocker B, and Rueckert D, “Attention U-Net: Learning where to look for the pancreas,” 2018, arXiv:1804.03999.

[173] Wang Y, Wang T, Li H, and Wang H, “ACF-TransUNet: Attention-based coarse-fine transformer U-Net for automatic liver tumor segmentation in CT images,” in Proc. 4th Int. Conf. Big Data Artif. Intell. Softw. Eng. (ICBASE), Aug. 2023, pp. 84–88.

[174] Hu J, Shen L, Albanie S, Sun G, and Wu E, “Squeeze-and-excitation networks,” IEEE Trans. Pattern Anal. Mach. Intell, vol. 42, no. 8, pp. 2011–2023, Aug. 2020. 31034408 10.1109/TPAMI.2019.2913372

[175] Wang X, Zhang X, Wang G, Zhang Y, Shi X, Dai H, Liu M, Wang Z, and Meng X, “TransFusionNet: Semantic and spatial features fusion framework for liver tumor and vessel segmentation under jetsonTX2,” IEEE J. Biomed. Health Informat, vol. 27, no. 3, pp. 1173–1184, Mar. 2023. 10.1109/JBHI.2022.3207233 36112546

[176] Yuan Y, Wang B, Zhang C, Xu J, Liu X, and Zhu L, “MFA-Net: Multi-scale feature fusion attention network for liver tumor segmentation,” 2024, arXiv:2405.04064.

[177] Kushnure DT and Talbar SN, “MS-UNet: A multi-scale UNet with feature recalibration approach for automatic liver and tumor segmentation in CT images,” Computerized Med. Imag. Graph, vol. 89, Apr. 2021, Art. no. 101885. 10.1016/j.compmedimag.2021.101885 33684731

[178] Kushnure DT and Talbar SN, “HFRU-Net: High-level feature fusion and recalibration UNet for automatic liver and tumor segmentation in CT images,” Comput. Methods Programs Biomed, vol. 213, Jan. 2022, Art. no. 106501. 10.1016/j.cmpb.2021.106501 34752959

[179] Zhao P, Zhang J, Fang W, and Deng S, “SCAU-Net: Spatial-channel attention U-Net for gland segmentation,” Frontiers Bioengineering Biotechnol, vol. 8, Jul. 2020, Art. no. 670. 10.3389/fbioe.2020.00670 PMC7347985 32719781

[180] Pang S, Du A, Orgun MA, Wang Y, and Yu Z, “Tumor attention networks: Better feature selection, better tumor segmentation,” Neural Netw, vol. 140, pp. 203–222, Aug. 2021. 33780873 10.1016/j.neunet.2021.03.006

[181] Woo S, Park J, Lee J-Y, and So Kweon I, “CBAM: Convolutional block attention module,” 2018, arXiv:1807.06521.

[182] Abian AI, Debnath RK, Rahman MA, Raiaan MAK, Islam MR, Karim A, Mohamed RE, and Azam S, “HANS-Net: Hyperbolic convolution and adaptive temporal attention for accurate and generalizable liver and tumor segmentation in CT imaging,” 2025, arXiv:2507.11325.

[183] Zhao X, Wu Z, Tan S, Fan D-J, Li Z, Wan X, and Li G, “Semi-supervised spatial temporal attention network for video polyp segmentation,” in Proc. Int. Conf. Med. Image Comput. Comput.-Assist. Intervent, 2020, pp. 456–466.

[184] Wan P, Chen F, Liu C, Kong W, and Zhang D, “Hierarchical temporal attention network for thyroid nodule recognition using dynamic CEUS imaging,” IEEE Trans. Med. Imag, vol. 40, no. 6, pp. 1646–1660, Jun. 2021. 10.1109/TMI.2021.3063421 33651687

[185] Shao J, Luan S, Ding Y, Xue X, Zhu B, and Wei W, “Attention connect network for liver tumor segmentation from CT and MRI images,” Technol. Cancer Res. Treatment, vol. 23, Jan. 2024, Art. no. 15330338231219366. 10.1177/15330338231219366 PMC10771068 38179668

[186] Huang Z, Wang X, Huang L, Huang C, Wei Y, and Liu W, “CCNet: Cris-cross attention for semantic segmentation,” in Proc. IEEE/CVF Int. Conf. Comput. Vis. (ICCV), Oct. 2019, pp. 603–612.

[187] Qiang L, Song H, Zhang W, Fan J, Ai D, Lin Y, and Yang J, “CC-DenseUNet: Densely connected U-Net with criss-cross attention for liver and tumor segmentation in CT volumes,” in Proc. IEEE Int. Conf. Bioinf. Biomed. (BIBM), Feb. 2021, pp. 966–971.

[188] Xiao H, Li L, Liu Q, Zhu X, and Zhang Q, “Transformers in medical image segmentation: A review,” Biomed. Signal Process. Control, vol. 84, Jul. 2023, Art. no. 104791.

[189] Lyu P, Liu W, Lin T, Zhang J, Liu Y, Wang C, and Zhu J, “Semi-supervised segmentation of abdominal organs and liver tumor: Uncertainty rectified curriculum labeling meets X-fuse,” Mach. Learn., Sci. Technol, vol. 5, no. 2, Jun. 2024, Art. no. 025047.

[190] Liu H, Yang J, Jiang C, He S, Fu Y, Zhang S, Hu X, Fang J, and Ji W, “S2DA-Net: Spatial and spectral-learning double-branch aggregation network for liver tumor segmentation in CT images,” Comput. Biol. Med, vol. 174, May 2024, Art. no. 108400. 10.1016/j.compbiomed.2024.108400 38613888

[191] Raghaw CS, Sanjotra JS, Rehman MZU, Bansal S, Dar SS, and Kumar N, “T-MPEDNet: Unveiling the synergy of transformer-aware multiscale progressive encoder–decoder network with feature recalibration for tumor and liver segmentation,” Biomed. Signal Process. Control, vol. 110, Dec. 2025, Art. no. 108225.

[192] Dosovitskiy A, Beyer L, Kolesnikov A, Weissenborn D, Zhai X, Unterthiner T, Dehghani M, Minderer M, Heigold G, Gelly S, Uszkoreit J, and Houlsby N, “An image is worth 16x16 words: Transformers for image recognition at scale,” 2020, arXiv:2010.11929.

[193] Di S, Zhao Y-Q, Liao M, Zhang F, and Li X, “TD-Net: A hybrid end-to-end network for automatic liver tumor segmentation from CT images,” IEEE J. Biomed. Health Informat, vol. 27, no. 3, pp. 1163–1172, Mar. 2023. 10.1109/JBHI.2022.3181974 35696476

[194] Li R, Xu L, Xie K, Song J, Ma X, Chang L, and Yan Q, “DHT-Net: Dynamic hierarchical transformer network for liver and tumor segmentation,” IEEE J. Biomed. Health Informat, vol. 27, no. 7, pp. 3443–3454, Jul. 2023. 10.1109/JBHI.2023.3268218 37079414

[195] Ni Y, Chen G, Feng Z, Cui H, Metaxas D, Zhang S, and Zhu W, “DA-tran: Multiphase liver tumor segmentation with a domain-adaptive transformer network,” Pattern Recognit., vol. 149, May 2024, Art. no. 110233.

[196] Liu Z, Lin Y, Cao Y, Hu H, Wei Y, Zhang Z, Lin S, and Guo B, “Swin transformer: Hierarchical vision transformer using shifted windows,” in Proc. IEEE/CVF Int. Conf. Comput. Vis. (ICCV), Oct. 2021, pp. 9992–10002.

[197] Hille G, Agrawal S, Tummala P, Wybranski C, Pech M, Surov A, and Saalfeld S, “Joint liver and hepatic lesion segmentation in MRI using a hybrid CNN with transformer layers,” Comput. Methods Programs Biomed, vol. 240, Oct. 2023, Art. no. 107647. 10.1016/j.cmpb.2023.107647 37329803

[198] Jiang L, Hu J, and Huang T, “Improved SwinUNet with fusion transformer and large kernel convolutional attention for liver and tumor segmentation in CT images,” Sci. Rep, vol. 15, no. 1, p. 14286, Apr. 2025. 40274913 10.1038/s41598-025-98938-5 PMC12022277

[199] Yang B, Zhang J, Lyu Y, and Zhang J, “Automatic computed tomography image segmentation method for liver tumor based on a modified tokenized multilayer perceptron and attention mechanism,” Quant. Imag. Med. Surgery, vol. 15, no. 3, pp. 2385–2404, Mar. 2025. 10.21037/qims-24-2132 PMC11948385 40160629

[200] Zhou T, Xia W, Zhang F, Chang B, Wang W, Yuan Y, Konukoglu E, and Cremers D, “Image segmentation in foundation model era: A survey,” 2024, arXiv:2408.12957.

[201] Kojima T, Shane Gu S, Reid M, Matsuo Y, and Iwasawa Y, “Large language models are zero-shot reasoners,” 2022, arXiv:2205.11916.

[202] Zhang J, Huang J, Jin S, and Lu S, “Vision-language models for vision tasks: A survey,” IEEE Trans. Pattern Anal. Mach. Intell, vol. 46, no. 8, pp. 5625–5644, Aug. 2024. 38408000 10.1109/TPAMI.2024.3369699

[203] Radford A, Kim JW, Hallacy C, Ramesh A, Goh G, Agarwal S, Sastry G, Askell A, Mishkin P, Clark J, Krueger G, and Sutskever I, “Learning transferable visual models from natural language supervision,” in Proc. Int. Conf. Mach. Learn, 2022, pp. 1–23.

[204] Jia C, Yang Y, Xia Y, Chen Y-T, Parekh Z, Pham H, Le QV, Sung Y, Li Z, and Duerig T, “Scaling up visual and vision-language representation learning with noisy text supervision,” 2021, arXiv:2102.05918.

[205] Kirillov AM, Mintun E, Ravi N, Mao H, Rolland C, Gustafson L, Xiao T, Whitehead S, Berg AC, Lo W, Dollár P, and Girshick R, “Segment anything,” in Proc. IEEE/CVF Int. Conf. Comput. Vis. (ICCV), Jun. 2023, pp. 3992–4003.

[206] Wu Y, Wang Z, Yang X, Kang H, He A, and Li T, “Trans-SAM: Transfer segment anything model to medical image segmentation with parameter-efficient fine-tuning,” Knowl.-Based Syst, vol. 310, Feb. 2025, Art. no. 112909.

[207] Mazurowski MA, Dong H, Gu H, Yang J, Konz N, and Zhang Y, “Segment anything model for medical image analysis: An experimental study,” Med. Image Anal, vol. 89, Oct. 2023, Art. no. 102918. 10.1016/j.media.2023.102918 PMC10528428 37595404

[208] Cheng D, Qin Z, Jiang Z, Zhang S, Lao Q, and Li K, “SAM on medical images: A comprehensive study on three prompt modes,” 2023, arXiv:2305.00035.

[209] Zhang Y, Shen Z, and Jiao R, “Segment anything model for medical image segmentation: Current applications and future directions,” Comput. Biol. Med, vol. 171, Mar. 2024, Art. no. 108238. 10.1016/j.compbiomed.2024.108238 38422961

[210] Hu C, Xia T, Ju S, and Li X, “When SAM meets medical images: An investigation of segment anything model (SAM) on multi-phase liver tumor segmentation,” 2023, arXiv:2304.08506.

[211] Mattjie C, De Moura LV, Ravazio R, Kupssinskü L, Parraga O, Delucis MM, and Barros RC, “Zero-shot performance of the segment anything model (SAM) in 2D medical imaging: A comprehensive evaluation and practical guidelines,” in Proc. IEEE 23rd Int. Conf. Bioinf. Bioengineering (BIBE), Dec. 2023, pp. 108–112.

[212] Xie B, Tang H, Duan B, Cai D, Yan Y, and Agam G, “MaskSAM: Towards auto-prompt SAM with mask classification for volumetric medical image segmentation,” 2024, arXiv:2403.14103.

[213] Hu M, Li Y, and Yang X, “SkinSAM: Empowering skin cancer segmentation with segment anything model,” 2023, arXiv:2304.13973.

[214] Li J, Hu M, and Yang X, “Polyp-SAM: Transfer SAM for polyp segmentation,” Proc. SPIE, vol. 12927, p. 117, Apr. 2024.

[215] Hu X, Xu X, and Shi Y, “How to efficiently adapt large segmentation model(SAM) to medical images,” 2023, arXiv:2306.13731.

[216] Feng W, Zhu L, and Yu L, “Cheap lunch for medical image segmentation by fine-tuning SAM on few exemplars,” in Proc. Int. MICCAI Brainlesion Workshop, 2023, pp. 13–22.

[217] Hin Lee H, Gu Y, Zhao T, Xu Y, Yang J, Usuyama N, Wong C, Wei M, Landman BA, Huo Y, Santamaria-Pang A, and Poon H, “Foundation models for biomedical image segmentation: A survey,” 2024, arXiv:2401.07654.

[218] Cui C, Deng R, Liu Q, Yao T, Bao S, Remedios LW, Landman BA, Tang Y, and Huo Y, “All-in-SAM: From weak annotation to pixel-wise nuclei segmentation with prompt-based finetuning,” J. Phys., Conf. Ser, vol. 2722, no. 1, Mar. 2024, Art. no. 012012. 10.1088/1742-6596/2722/1/012012 PMC11925546 40115612

[219] Gu Y, Wu Q, Tang H, Mai X, Shu H, Li B, and Chen Y, “LeSAM: Adapt segment anything model for medical lesion segmentation,” IEEE J. Biomed. Health Informat, vol. 28, no. 10, pp. 6031–6041, Oct. 2024. 10.1109/JBHI.2024.3406871 38809720

[220] Paranjape JN, Sikder S, Vedula SS, and Patel VM, “S-SAM: SVD-based fine-tuning of segment anything model for medical image segmentation,” in Proc. Int. Conf. Med. Image Comput. Comput.-Assisted Intervent, 2024, pp. 720–730.

[221] Zhang K and Liu D, “Customized segment anything model for medical image segmentation,” 2023, arXiv:2304.13785.

[222] Chen S, Lin L, Cheng P, and Tang X, “ASLseg: Adapting SAM in the loop for semi-supervised liver tumor segmentation,” 2023, arXiv:2312.07969.

[223] Zhu J, Hamdi A, Qi Y, Jin Y, and Wu J, “Medical SAM 2: Segment medical images as video via segment anything model 2,” 2024, arXiv:2408.00874.

[224] Shen C, Li W, Shi Y, and Wang X, “Interactive 3D medical image segmentation with SAM 2,” 2024, arXiv:2408.02635.

[225] Sengupta S, Chakrabarty S, and Soni R, “Is SAM 2 better than SAM in medical image segmentation?” 2024, arXiv:2408.04212.

[226] Chen C, Miao J, Wu D, Zhong A, Yan Z, Kim S, Hu J, Liu Z, Sun L, Li X, Liu T, Heng P-A, and Li Q, “MA-SAM: Modality-agnostic SAM adaptation for 3D medical image segmentation,” Med. Image Anal, vol. 98, Dec. 2024, Art. no. 103310. 10.1016/j.media.2024.103310 PMC11381141 39182302

[227] Lin H, Zou J, Deng S, Wong KP, Aviles-Rivero AI, Fan Y, Lee AP-W, Hu X, and Qin J, “Volumetric medical image segmentation via fully 3D adaptation of segment anything model,” Biocybernetics Biomed. Eng, vol. 45, no. 1, pp. 1–10, Jan. 2025.

[228] Kuang Y, Ma X, Zhao J, Wang G, Zeng Y, and Liu S, “A novel 3D medical image segmentation model using improved SAM,” in Proc. IEEE Int. Conf. Syst, Mar. 2024, pp. 2528–2534.

[229] Gong S, Zhong Y, Ma W, Li J, Wang Z, Zhang J, Heng P-A, and Dou Q, “3DSAM-adapter: Holistic adaptation of SAM from 2D to 3D for promptable tumor segmentation,” Med. Image Anal, vol. 98, Dec. 2024, Art. no. 103324. 10.1016/j.media.2024.103324 39213939

[230] Gao X and Lu K, “RefSAM3D: Adapting SAM with cross-modal reference for 3D medical image segmentation,” 2024, arXiv:2412.05605.

[231] Chen F, Tang J, Wang P, Wang T, Li S, and Deng T, “DEAP-3DSAM: Decoder enhanced and auto prompt SAM for 3D medical image segmentation,” in Proc. IEEE Int. Conf. Bioinf. Biomed. (BIBM), Dec. 2024, pp. 1852–1859.

[232] Wu J, Ji W, Liu Y, Fu H, Xu M, Xu Y, and Jin Y, “Medical SAM adapter: Adapting segment anything model for medical image segmentation,” 2023, arXiv:2304.12620. 10.1016/j.media.2025.103547 40121809

[233] Häkkinen I, Melekhov I, Englesson E, Azizpour H, and Kannala J, “Medical image segmentation with SAM-generated annotations,” 2024, arXiv:2409.20253.

[234] Zhang Y, Yuan C, and Qi Y, “SemiSAM: Enhancing semi-supervised medical image segmentation via SAM-assisted consistency regularization,” in Proc. IEEE Int. Conf. Bioinf. Biomed. (BIBM), Dec. 2023, pp. 3982–3986.

[235] Tajbakhsh N, Jeyaseelan L, Li Q, Chiang JN, Wu Z, and Ding X, “Embracing imperfect datasets: A review of deep learning solutions for medical image segmentation,” Med. Image Anal, vol. 63, Jul. 2020, Art. no. 101693. 10.1016/j.media.2020.101693 32289663

[236] Cheplygina V, de Bruijne M, and Pluim JPW, “Not-so-supervised: A survey of semi-supervised, multi-instance, and transfer learning in medical image analysis,” Med. Image Anal, vol. 54, pp. 280–296, May 2019. 30959445 10.1016/j.media.2019.03.009

[237] Al-Kababji A, Bensaali F, Dakua SP, and Himeur Y, “Automated liver tissues delineation techniques: A systematic survey on machine learning current trends and future orientations,” Eng. Appl. Artif. Intell, vol. 117, Jan. 2023, Art. no. 105532.

[238] Ghobadi V, Ismail LI, Wan Hasan WZ, Ahmad H, Ramli HR, Norsahperi NMH, Tharek A, and Hanapiah FA, “Challenges and solutions of deep learning-based automated liver segmentation: A systematic review,” Comput. Biol. Med, vol. 185, Feb. 2025, Art. no. 109459. 10.1016/j.compbiomed.2024.109459 39642700

[239] Defeudis A, Panić J, Guzzinati W, Pusceddu L, Vassallo L, Regge D, and Giannini V, “A deep learning model to segment liver metastases on CT images acquired at different time-points during chemotherapy,” in Proc. EEE Int. Symp. Med. Meas. Appl. (MeMeA), pp. 1–6, Jun. 2022.

[240] Zhang X, Xie W, Huang C, Wang Y, Zhang Y, Chen X, and Tian Q, “Self-supervised tumor segmentation through layer decomposition,” 2021, arXiv:2109.03230.

[241] Shaban A, Bansal S, Liu Z, Essa I, and Boots B, “One-shot learning for semantic segmentation,” 2017, arXiv:1709.03410.

[242] Lang C, Cheng G, Tu B, and Han J, “Learning what not to segment: A new perspective on few-shot segmentation,” in Proc. IEEE/CVF Conf. Comput. Vis. Pattern Recognit. (CVPR), Jun. 2022, pp. 8047–8057.

[243] Kim Y, Kang D, Mok Y, Kwon S, and Paik J, “A review on few-shot learning for medical image segmentation,” in Proc. Int. Conf. Electron, 2023, pp. 1–3.

[244] Adadi A, “A survey on data-efficient algorithms in big data era,” J. Big Data, vol. 8, no. 1, p. 24, Jan. 2021.

[245] Ren P, Xiao Y, Chang X, Huang P-Y, Li Z, Gupta BB, Chen X, and Wang X, “A survey of deep active learning,” ACM Comput. Surveys (CSUR), vol. 55, no. 7, pp. 1–38, Aug. 2023.

[246] Chapelle O, Schölkopf B, and Zien A, Introduction to Semi-supervised Learning. Cambridge, MA, USA: MIT Press, 2006.

[247] Liu X, Zhang F, Hou Z, Mian L, Wang Z, Zhang J, and Tang J, “Self-supervised learning: Generative or contrastive,” IEEE Trans. Knowl. Data Eng, vol. 35, no. 1, pp. 857–876, Jan. 2023.

[248] Zhou Z-H, “A brief introduction to weakly supervised learning,” Nat. Sci. Rev, vol. 5, no. 1, pp. 44–53, Jan. 2018.

[249] Burmeister J-M, Fernandez Rosas M, Hagemann J, Kordt J, Blum J, Shabo S, Bergner B, and Lippert C, “Less is more: A comparison of active learning strategies for 3D medical image segmentation,” 2022, arXiv:2207.00845.

[250] Zheng Z and Yang Y, “Rectifying pseudo label learning via uncertainty estimation for domain adaptive semantic segmentation,” Int. J. Comput. Vis, vol. 129, no. 4, pp. 1106–1120, Apr. 2021.

[251] Zhuang J, Luo L, Chen Z, and Wu L, “Iterative semi-supervised learning for abdominal organs and tumor segmentation,” 2023, arXiv:2310.01159.

[252] Zhang X, Xie W, Huang C, Zhang Y, Chen X, Tian Q, and Wang Y, “Self-supervised tumor segmentation with Sim2Real adaptation,” IEEE J. Biomed. Health Informat, vol. 27, no. 9, pp. 4373–4384, Sep. 2023. 10.1109/JBHI.2023.3240844 37022235

[253] Feng X, Yang J, Laine AF, and Angelini ED, “Discriminative localization in CNNs for weakly-supervised segmentation of pulmonary nodules,” in Proc. MICCAI, vol. 10435, 2017, pp. 568–576. 10.1007/978-3-319-66179-7_65 PMC5753796 29308456

[254] Wang H, Yi F, Wang J, Yi Z, and Zhang H, “RECISTSup: Weakly-supervised lesion volume segmentation using RECIST measurement,” IEEE Trans. Med. Imag, vol. 41, no. 7, pp. 1849–1861, Jul. 2022. 10.1109/TMI.2022.3149168 35120001

[255] Liu Y, Halek S, Li L, Tomaszewski MR, Wang S, Baumgartner R, Yuan J, Goldmacher G, and Chen A, “Universal 3D CT lesion segmentation using SAM with RECIST annotation,” Med. Imag, vol. 12926, p. 4, Apr. 2024.

[256] Chu T, Li X, Vo HV, Summers RM, and Sizikova E, “Improving weakly supervised lesion segmentation using multi-task learning,” in Proc. 24th Int. Conf. Artif. Intell. Statist, Mar. 2021, pp. 60–73. [Online]. Available: https://proceedings.mlr.press/v143/chu21a.html

[257] Cai J, Tang Y, Lu L, Harrison AP, Yan K, Xiao J, Yang L, and Summers RM, “Accurate weakly-supervised deep lesion segmentation using large-scale clinical annotations: Slice-propagated 3D mask generation from 2D RECIST,” 2018, arXiv:1807.01172.

[258] Tan CH, Hu Y, and Saeed SU, “SPARS: Self-play adversarial reinforcement learning for segmentation of liver tumours,” in Proc. Annu. Conf. Med. Image Understand. Anal, 2025, pp. 59–72.

[259] Sailer AM, Douwes DC, Cappendijk VC, Bakers FC, Wagemans BA, Wildberger JE, Kessels AG, and Beets-Tan RG, “RECIST measurements in cancer treatment: Is there a role for physician assistants?—A pilot study,” Cancer Imag, vol. 14, no. 1, p. 12, Dec. 2014. 10.1186/1470-7330-14-12 PMC4331818 25608556

[260] Fasel JHD and Schenk A, “Concepts for liver segment classification: Neither old ones nor new ones, but a comprehensive one,” J. Clin. Imag. Sci, vol. 3, p. 48, Oct. 2013. 10.4103/2156-7514.120803 PMC3823389 24228216

[261] Chen Y, Yue X, Zhong C, and Wang G, “Functional region annotation of liver CT image based on vascular tree,” BioMed Res. Int, vol. 2016, pp. 1–13, Jan. 2016. 10.1155/2016/5428737 PMC5116550 27891516

[262] Ma J, Chen J, Ng M, Huang R, Li Y, Li C, Yang X, and Martel AL, “Loss Odyssey in medical image segmentation,” Med. Image Anal, vol. 71, Jul. 2021, Art. no. 102035. 10.1016/j.media.2021.102035 33813286

[263] Gul S, Khan MS, Bibi A, Khandakar A, Ayari MA, and Chowdhury MEH, “Deep learning techniques for liver and liver tumor segmentation: A review,” Comput. Biol. Med, vol. 147, Aug. 2022, Art. no. 105620. 10.1016/j.compbiomed.2022.105620 35667155

[264] de Boer P-T, Kroese DP, Mannor S, and Rubinstein RY, “A tutorial on the cross-entropy method,” Ann. Operations Res, vol. 134, no. 1, pp. 19–67, Feb. 2005.

[265] Shelhamer E, Long J, and Darrell T, “Fully convolutional networks for semantic segmentation,” IEEE Trans. Pattern Anal. Mach. Intell, vol. 39, no. 4, pp. 640–651, Apr. 2017. 27244717 10.1109/TPAMI.2016.2572683

[266] Jaccard P, “Étude comparative de la distribution florale dans une portion des Alpes et des Jura,” Bulletin de la Societe Vaudoise des Sci. Naturelles, vol. 37, no. 3, pp. 547–604, Jan. 1901.

[267] Tversky A, “Features of similarity,” Psychol. Rev, vol. 84, no. 4, p. 327, 1977.

[268] Salehi SSM, Erdoğmuş D, and Gholipour A, “Tversky loss function for image segmentation using 3D fully convolutional deep networks,” in Proc. MLMI@MICCAI, 2017, pp. 379–387.

[269] Lin T-Y, Goyal P, Girshick R, He K, and Dollár P, “Focal loss for dense object detection,” in Proc. IEEE Int. Conf. Comput. Vis. (ICCV), Oct. 2017, pp. 2999–3007. 10.1109/TPAMI.2018.2858826 30040631

[270] Abraham N and Khan NM, “A novel focal Tversky loss function with improved attention U-Net for lesion segmentation,” in Proc. IEEE 16th Int. Symp. Biomed. Imag. (ISBI), Apr. 2019, pp. 683–687.

[271] El Jurdi R, Petitjean C, Honeine P, Cheplygina V, and Abdallah F, “High-level prior-based loss functions for medical image segmentation: A survey,” 2020, arXiv:2011.08018.

[272] Chen Z, Tong J, Jiang N, and Pan Z, “AD-UNet: A multi-attention mechanism densely connected U-Net for liver tumor segmentation,” Available SSRN, 2023. [Online]. Available: 10.2139/ssrn.4385949

[273] Kervadec H, Bouchtiba J, Desrosiers C, Granger E, Dolz J, and Ben Ayed I, “Boundary loss for highly unbalanced segmentation,” Med. Image Anal, vol. 67, Jan. 2021, Art. no. 101851. 10.1016/j.media.2020.101851 33080507

[274] Li R, Wang X, Huang G, Yang W, Zhang K, Gu X, Tran SN, Garg S, Alty J, and Bai Q, “A comprehensive review on deep supervision: Theories and applications,” 2022, arXiv:2207.02376.

[275] Peng K, Fang B, and Zhou M, “Cascaded deeply supervised convolutional networks for liver lesion segmentation,” Int. J. Pattern Recognit. Artif. Intell, vol. 35, no. 10, Aug. 2021, Art. no. 2152014.

[276] Xue Y, Xu T, Zhang H, Long LR, and Huang X, “SegAN: Adversarial network with multi-scale L1 loss for medical image segmentation,” Neuroinformatics, vol. 16, nos. 3–4, pp. 383–392, Oct. 2018. 29725916 10.1007/s12021-018-9377-x PMC13344194

[277] Luc P, Couprie C, Chintala S, and Verbeek J, “Semantic segmentation using adversarial networks,” 2016, arXiv:1611.08408.

[278] Tran S-T, Cheng C-H, and Liu D-G, “A multiple layer U-Net, UN-Net, for liver and liver tumor segmentation in CT,” IEEE Access, vol. 9, pp. 3752–3764, 2021.

[279] Tang Y, Tang Y, Zhu Y, Xiao J, and Summers RM, “E^2^Net: An edge enhanced network for accurate liver and tumor segmentation on CT scans,” in Proc. Int. Conf. Med. Image Comput. Comput.-Assisted Intervent (MICCAI). Cham, Switzerland: Springer, Oct. 2021, pp. 512–522.

[280] Yushkevich PA, Piven J, Hazlett HC, Smith RG, Ho S, Gee JC, and Gerig G, “User-guided 3D active contour segmentation of anatomical structures: Significantly improved efficiency and reliability,” NeuroImage, vol. 31, no. 3, pp. 1116–1128, Jul. 2006. 16545965 10.1016/j.neuroimage.2006.01.015

[281] Yashaswini GN, Manjunath RV, Shubha B, Prabha P, Aishwarya N, and Manu HM, “Deep learning technique for automatic liver and liver tumor segmentation in CT images,” J. Liver Transplantation, vol. 17, Feb. 2025, Art. no. 100251.

[282] Smyth P, Fayyad UM, Burl MC, Perona P, and Baldi P, “Inferring ground truth from subjective labelling of Venus images,” in Proc. Neural Inf. Process. Syst, vol. 7, 1994, pp. 1085–1092.

[283] McHugh ML, “Interrater reliability: The Kappa statistic,” Biochemia Medica, vol. 22, no. 3, pp. 276–282, 2012. 23092060 PMC3900052

[284] Garnavi R, Aldeen M, Celebi ME, Varigos G, and Finch S, “Border detection in dermoscopy images using hybrid thresholding on optimized color channels,” Computerized Med. Imag. Graph, vol. 35, no. 2, pp. 105–115, Mar. 2011. 10.1016/j.compmedimag.2010.08.001 20832992

[285] Iyatomi H, Oka H, Celebi ME, Hashimoto M, Hagiwara M, Tanaka M, and Ogawa K, “An improved internet-based melanoma screening system with dermatologist-like tumor area extraction algorithm,” Computerized Med. Imag. Graph, vol. 32, no. 7, pp. 566–579, Oct. 2008. 10.1016/j.compmedimag.2008.06.005 18703311

[286] Rohlfing T and Maurer CR, “Shape-based averaging,” IEEE Trans. Image Process, vol. 16, no. 1, pp. 153–161, Jan. 2007. 17283774 10.1109/tip.2006.884936

[287] Chalana V and Kim Y, “A methodology for evaluation of boundary detection algorithms on medical images,” IEEE Trans. Med. Imag, vol. 16, no. 5, pp. 642–652, May 1997. 10.1109/42.640755 9368120

[288] Warfield SK, Zou KH, and Wells WM, “Simultaneous truth and performance level estimation (STAPLE): An algorithm for the validation of image segmentation,” IEEE Trans. Med. Imag, vol. 23, no. 7, pp. 903–921, Jul. 2004. 10.1109/TMI.2004.828354 PMC1283110 15250643

[289] Langerak TR, van der Heide UA, Kotte ANTJ, Viergever MA, van Vulpen M, and Pluim JPW, “Label fusion in atlas-based segmentation using a selective and iterative method for performance level estimation (SIMPLE),” IEEE Trans. Med. Imag, vol. 29, no. 12, pp. 2000–2008, Dec. 2010. 10.1109/TMI.2010.2057442 20667809

[290] Taha AA and Hanbury A, “Metrics for evaluating 3D medical image segmentation: Analysis, selection, and tool,” BMC Med. Imag, vol. 15, no. 1, pp. 1–28, Dec. 2015. 10.1186/s12880-015-0068-x PMC4533825 26263899

[291] Miller GA and Nicely PE, “An analysis of perceptual confusions among some English consonants,” J. Acoust. Soc. Amer, vol. 27, no. 2, pp. 338–352, Mar. 1955.

[292] Chen Y, Gong G, Wang Y, Liu C, Su Y, Wang L, Yang B, and Yin Y, “Comparative evaluation of 4-Dimensional computed tomography and 4-Dimensional magnetic resonance imaging to delineate the target of primary liver cancer,” Technol. Cancer Res. Treatment, vol. 20, Jan. 2021, Art. no. 15330338211045499. 10.1177/15330338211045499 PMC8504652 34617855

[293] Pang EHT, Chan A, Ho SG, and Harris AC, “Contrast-enhanced ultrasound of the liver: Optimizing technique and clinical applications,” Amer. J. Roentgenology, vol. 210, no. 2, pp. 320–332, Feb. 2018. 10.2214/AJR.17.17843 29220210

[294] Shen D, Wu G, and Suk H, “Deep learning in medical image analysis,” Annu. Rev. Biomed. Eng, vol. 19, no. 1, pp. 221–248, 2017. 28301734 10.1146/annurev-bioeng-071516-044442 PMC5479722

[295] Lundervold AS and Lundervold A, “An overview of deep learning in medical imaging focusing on MRI,” Zeitschrift für Medizinische Physik, vol. 29, no. 2, pp. 102–127, May 2019. 30553609 10.1016/j.zemedi.2018.11.002

[296] Kumar SS and Kumar RSV, “Literature survey on deep learning methods for liver segmentation from CT images: A comprehensive review,” Multimedia Tools Appl, vol. 83, no. 28, pp. 71833–71862, Feb. 2024.

[297] Dinsdale NK, Bluemke E, Sundaresan V, Jenkinson M, Smith SM, and Namburete AIL, “Challenges for machine learning in clinical translation of big data imaging studies,” Neuron, vol. 110, no. 23, pp. 3866–3881, Dec. 2022. 36220099 10.1016/j.neuron.2022.09.012

[298] Pineau J, Vincent-Lamarre P, Sinha K, Larivière V, Beygelzimer A, d’Alché–Buc F, Fox EB, and Larochelle H, “Improving reproducibility in machine learning research (A report from the NeurIPS 2019 reproducibility program),” J. Mach. Learn. Res, pp. 1–20, 2022.

[299] Litjens G, Kooi T, Bejnordi BE, Setio AAA, Ciompi F, Ghafoorian M, Van Der Laak JA, Van Ginneken B, and Sánchez CI, “A survey on deep learning in medical image analysis,” Med. Image Anal, vol. 42, pp. 60–88, Dec. 2017. 28778026 10.1016/j.media.2017.07.005

[300] Ho J, Jain A, and Abbeel P, “Denoising diffusion probabilistic models,” in Proc. Adv. Neural Inf. Process. Syst, vol. 33, 2020, pp. 6840–6851.

[301] Xie X, Niu J, Liu X, Chen Z, Tang S, and Yu S, “A survey on incorporating domain knowledge into deep learning for medical image analysis,” Med. Image Anal, vol. 69, Apr. 2021, Art. no. 101985. 10.1016/j.media.2021.101985 33588117

[302] Diao Z, Jiang H, and Zhou Y, “Leverage prior texture information in deep learning-based liver tumor segmentation: A plug-and-play texture-based auto pseudo label module,” Computerized Med. Imag. Graph, vol. 106, Jun. 2023, Art. no. 102217. 10.1016/j.compmedimag.2023.102217 36958076

[303] Chernyak V, Fowler KJ, Kamaya A, Kielar AZ, Elsayes KM, Bashir MR, Kono Y, Do RK, Mitchell DG, Singal AG, Tang A, and Sirlin CB, “Liver imaging reporting and data system (LI-RADS) version 2018: Imaging of hepatocellular carcinoma in at-risk patients,” Radiology, vol. 289, no. 3, pp. 816–830, Dec. 2018. 30251931 10.1148/radiol.2018181494 PMC6677371

[304] Bobba PS, Sailer A, Pruneski JA, Beck S, Mozayan A, Mozayan S, Arango J, Cohan A, and Chheang S, “Natural language processing in radiology: Clinical applications and future directions,” Clin. Imag, vol. 97, pp. 55–61, May 2023. 10.1016/j.clinimag.2023.02.014 36889116

[305] Ma J, Yang H, Chou Y, Yoon J, Allison T, Komandur R, McDunn J, Tasneem A, Do RK, Schwartz LH, and Zhao B, “Generalizability of lesion detection and segmentation when ScaleNAS is trained on a large multi-organ dataset and validated in the liver,” Med. Phys, vol. 52, no. 2, pp. 1005–1018, Feb. 2025. 39576046 10.1002/mp.17504

